# Metal-organic frameworks for precise inclusion of single-stranded DNA and transfection in immune cells

**DOI:** 10.1038/s41467-018-03650-w

**Published:** 2018-04-03

**Authors:** Shuang Peng, Binglin Bie, Yangzesheng Sun, Min Liu, Hengjiang Cong, Wentao Zhou, Yucong Xia, Heng Tang, Hexiang Deng, Xiang Zhou

**Affiliations:** 1 Key Laboratory of Biomedical Polymers-Ministry of Education, College of Chemistry and Molecular Sciences, Wuhan University, 430072 Wuhan, China; 2 UC Berkeley-Wuhan University Joint Innovative Center, The Institute of Advanced Studies, Wuhan University, 430072 Wuhan, China

## Abstract

Effective transfection of genetic molecules such as DNA usually relies on vectors that can reversibly uptake and release these molecules, and protect them from digestion by nuclease. Non-viral vectors meeting these requirements are rare due to the lack of specific interactions with DNA. Here, we design a series of four isoreticular metal-organic frameworks (Ni-IRMOF-74-II to -V) with progressively tuned pore size from 2.2 to 4.2 nm to precisely include single-stranded DNA (ssDNA, 11–53 nt), and to achieve reversible interaction between MOFs and ssDNA. The entire nucleic acid chain is completely confined inside the pores providing excellent protection, and the geometric distribution of the confined ssDNA is visualized by X-ray diffraction. Two MOFs in this series exhibit excellent transfection efficiency in mammalian immune cells, 92% in the primary mouse immune cells (CD4+ T cell) and 30% in human immune cells (THP-1 cell), unrivaled by the commercialized agents (Lipo and Neofect).

## Abstract (web summary)

Non-viral vectors are important for transfection but can be limited in the uptake, protection and release of ssDNA. Here, the authors report on the design of metal-organic-framework vectors with precisely controlled pore geometry and demonstrate the vector in the transfection of immune cells.

## Introduction

Various non-viral vectors have been developed so far and they largely promoted the research on gene therapy and gene editing^1–4^. The release of DNA cargo using these vectors usually involves decomposition of their structure or requires outside stimuli^1–4^. Porous metal-organic frameworks (MOFs) are well-known for their ability to bind and release small gas and organic molecules in a precise manner^5–15^. However, few studies have exploited this precision in incorporating large biological molecules and releasing them on demand^16–22^. Thus far, proteins and DNA bound MOFs have been investigated and found to be either deficient in accommodating these molecules because of pore size limitation or exhibit such a strong binding as to impede their release without destroying the MOF host^23,24^.

Here in, we show that single-stranded DNA (ssDNA) of different length (11, 22, 33, and 53 nucleotides) can be selectively bound into a series of MOFs featuring pore sizes from 2.2 to 4.2 nm with two members exhibiting optimal binding strength to allow precise release of ssDNA into a wide range of cell types including primary mouse and human immune cells, with high transfection efficiency. Such performance is unrivaled by the commercialized non-viral vectors including Neofect and Lipo. We further show that these MOFs are also effective at delivering DNAzyme (an ssDNA of 33 nucleotides) to MCF-7 human breast cancer cells, and inhibit the expression of the EGR-1 gene. The fact that the MOF is architecturally stable and the flexibility with which porosity and pore size can be optimized ensures the high loading of ssDNA and its protection against degradation in physiological fluid and extracellular environment until it reaches the cell. The release of the ssDNA cargo is induced by an existing DNA target in the cell containing the complementary sequence (cDNA) instead of using outside stimuli (such as light and heat) that are required for other vector materials. The interior environment of Ni-IRMOF-74 series provides a specific interaction between pores and ssDNA, leading to a response to the binding with target sequence instead of a straightforward delivery process. These are the key factors at play in the process of uptake, protection, and release of ssDNA and the reasons for the observed effective transfection. The results of this study point to MOFs as viable non-viral vectors in intracellular ssDNA delivery, with potential extension to other gene therapy. And this work presents a unique method of regulation on the interaction through tuning pore sizes in a precise manner.

## Results

### Structure design of MOF vector

**Fig. 1:**
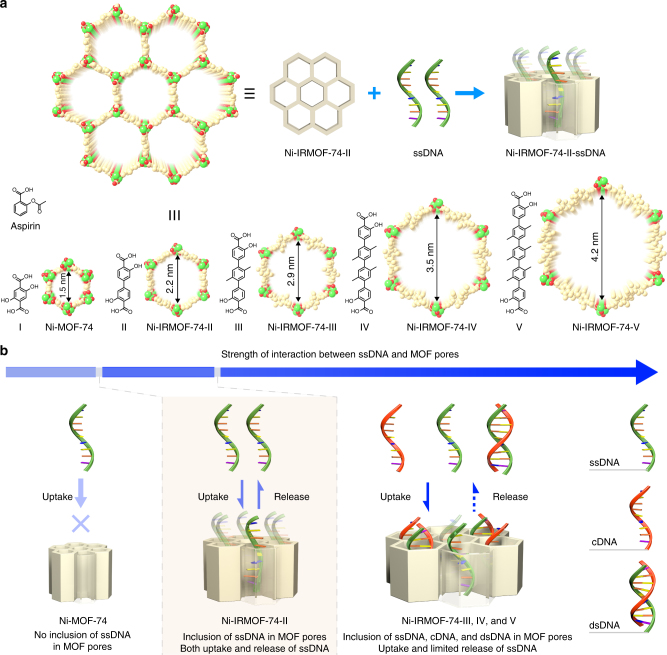
Fine-tuning of interactions between ssDNA and MOFs. **a** Illustration of ssDNA inclusion in MOFs composed of bio-compatible organic linkers and with precisely controlled pore sizes. Ni, C, and O atoms were labeled with green, gold, and red color, respectively. **b** Gradual increase of interaction between ssDNA and MOFs as the pore size of MOF extended progressively. Relatively mild interactions guarantee the uptake and protection of ssDNA in the MOF pores, and also provide reversibility for their release

In this study, the precise control of pore geometry and strength of interaction with guest molecules were achieved by the design and synthesis of a series of MOFs with the same topology but progressively increasing pore sizes (Fig. 1 and Supplementary Figure 1). These MOFs were constructed based on MOF-74, a robust framework with hexagonal topology (**etb** net) and one dimensional (1D) pores. The gradual pore size expansion was implemented by inserting multiple phenylene units into the original 2,5-dioxidoterephthalate linker to prepare organic linkers of different length (linker-II, -III, -IV, and -V) (Fig. 1a). The terminal units of these linkers are salicylic acid (Supplementary Figure 2 and 3), a basic functional building block of aspirin, offering excellent biocompatibility^25,26^. These linkers were coordinated with a divalent metal (Ni^2+^) through multiple oxygen atoms (Fig. 1a) to construct a series of four isoreticular MOFs, termed Ni-IRMOF-74-II, -III, -IV, and -V, respectively. These MOFs exhibit excellent crystallinity as evidenced by their sharp peaks in the powder X-ray diffraction (PXRD) patterns that match well with the simulated patterns based on the parent MOF and their analogs of Mg^27,28^ (Supplementary Figure 4, 6, 8, 10, and 12). Using the Rietveld method, the atomic structure of Ni-IRMOFs in this series was further validated and refined against experimental PXRD patterns collected with both synchrotron and laboratory based X-ray sources (Supplementary Figure 5, 7, 9, 11 and Supplementary Table 1–5). Based on the crystal structures, the pore sizes of these MOFs are fine-tuned from 2.2 to 4.2 nm (the diagonal dimension of pore size) for Ni-IRMOF-74-II to -V, respectively, with an increment of about 0.7 nm (Fig. 1a), thus offering precise pore environment to reveal the host–guest interactions (Fig. 1b).

**Fig. 2:**
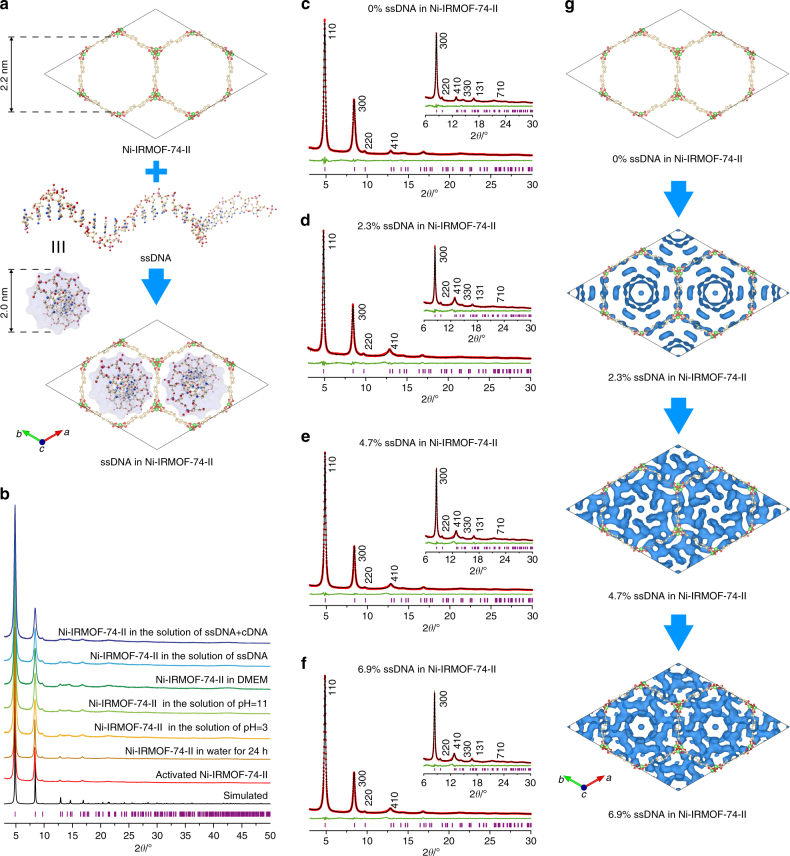
Visualization of precise ssDNA inclusion in the pore of Ni-IRMOF-74-II. **a** Excellent match between the size of ssDNA and that of Ni-IRMOF-74-II pores leads to inclusion of ssDNA with atomic accuracy. **b** PXRD patterns of Ni-IRMOF-74-II samples tested in various aqueous solutions. Wavelength of X-ray sources, *λ* = 1.5406 Å. **c–f** PXRD patterns of MOF samples loaded with different amounts of ssDNA in weight percent (0%, 2.3%, 4.7%, and 6.9%, respectively). Wavelength of X-ray sources, *λ* = 1.5406 Å. The experimental (red), refined (black), and difference (green) patterns are displayed. The Bragg positions are marked as pink bars. **g** Electron distribution map of ssDNA in Ni-IRMOF-74. Carbon (gold), nitrogen (blue), oxygen (red), and phosphorous (pink)

The permanent porosity of the Ni-IRMOF-74 series was further confirmed by N_2_ gas adsorption at 77 K. Ni-MOF-74 and Ni-IRMOF-74-II exhibit a Type I isotherm, while Ni-IRMOF-74-III, -IV, and -V exhibit a Type IV isotherm, typical for mesoporous materials (Supplementary Figure 21–30 and Supplementary Figure 64–71). Through quenched solid density function theory (QSDFT), the calculated pore sizes of Ni-IRMOFs were found to be 1.1, 1.8, 2.4, 3.0, and 3.6 nm (Supplementary Figure 21–30 and Supplementary Table 8 and 10) for Ni-MOF-74 and Ni-IRMOF-74-II to -V, respectively. These values are in good agreement with the gradual increase of the diagonal diameters in their crystal structures and matched well with the pore size distribution of reported Mg-IRMOF-74^27^. The surface area calculated by Brunauer-Emmett-Teller (BET) models are 1930, 2040, 1920, and 1900 m^2^/g for Ni-IRMOF-74-II to -V, respectively, while the pore volume of these MOFs varied from 0.33 to 1.39 cm^3^/g (Supplementary Table 8). The thermal stability of the Ni-IRMOF-74 series was demonstrated by thermogravimetric analysis (TGA) in air where no structure disposition was observed until 300 °C (Supplementary Figure 31–35). These MOFs exhibit excellent chemical stability in both aqueous solution and over a wide range of pH values (pH = 3 and pH = 11) (Fig. 2b and Supplementary Figure. 60). Unaltered sharp peaks were retained in the PXRD patterns of this Ni-IRMOF-74 series after 24-h immersion in water, basic and acidic aqueous solutions, and cell culture medium, which is in stark contrast to what is observed in the previously reported IRMOF-74 structures linked by Mg (Fig. 2b and Supplementary Figure 61).

The crystals obtained in the bulk synthesis of the Ni-IRMOF-74 series are needle shaped with a length of 0.5–1.5 μm and a cross-section in the range of 100–300 nm as revealed by scanning electron microscope (SEM) images (Supplementary Figure 40–44). In addition to the pore size requirement for the inclusion of biomolecules, the intracellular application of these materials also requires that the particle dimension falls within the nano-size regime. In order to achieve this, we further modified the synthetic conditions to obtain Ni-IRMOF-74-II and -III nanocrystals of particle size around 100 nm as confirmed by both dynamic light scattering (DLS) and SEM (Supplementary Figure 19, 20, 48, and 49 and Supplementary Table 7). The reduction of particle sizes is also reflected in the broadening of the diffraction peaks observed in their PXRD patterns (Supplementary Figure 17 and 18). The excellent chemical and thermal stability coupled with the pore size and nanoparticle morphology that can be precisely controlled make these Ni-IRMOF-74 series ready for inclusion of DNA and intracellular delivery.

### Inclusion of ssDNA into MOFs

**Fig. 3:**
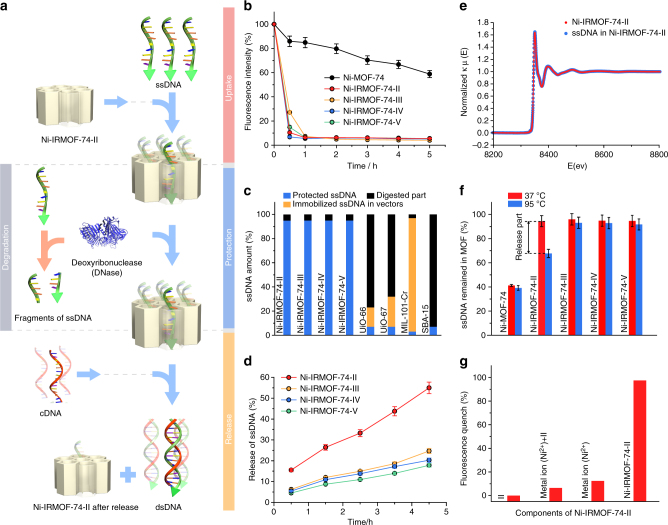
Uptake, protection, and release of ssDNA using MOFs as vectors. **a** Illustration of three critical processes in ssDNA transfection. **b** Uptake of ssDNA in Ni-IRMOF-74 samples (*n* = 3 technical replicates; bars represent mean ± s.d.). **c** Protection of ssDNA using various porous materials in FBS with 10% serum. Survived portion, residual left inside the vectors and digested portion of ssDNA are labeled in blue, yellow, and red, respectively. **d** Release of ssDNA from Ni-IRMOF-74 samples in the presence of cDNA (*n* = 3 technical replicates; bars represent mean ± s.d.). **e** X-ray absorption spectra of Ni-IRMOF-74-II before and after loading with ssDNA, blue and red, respectively. **f** Experiments that rule out the possibility of H-bond between ssDNA and MOFs. Ni-IRMOF-74 samples loaded with ssDNA were heated at 37 °C and 95 °C, red and blue, respectively (*n* = 3 technical replicates; bars represent mean ± s.d.). **g** Fluorescence quench of FAM-labeled ssDNA in contact with IRMOF-74-II and its components

Short single-stranded nucleic acid (ssDNA and ssRNA with less than 100 nucleotides) represents an important class of genetic materials that are widely applied in gene editing and gene modulation^29–32^. In this study, four ssDNA of different lengths (11, 22, 33, 53 nucleotides, Supplementary Figure 82 and Supplementary Table 11) were used in the inclusion test. The visualization and quantification of ssDNA uptake into the pores of the MOF samples was achieved by labeling the 5′ end of each ssDNA with 6-carboxyfluorescein (FAM), a motif that gives green fluorescence at a wavelength of 520 nm. When the labeled ssDNA entered the pores, FAM motifs were brought so close to the walls of the framework that electrons can be transferred from FAM to the MOFs which resulted in fluorescence quenching (Fig. 2a and Fig. 3a). Such phenomenon was not observed in the control experiment using the FAM molecule without linking to ssDNA (Supplementary Figure 58). The significant fluorescence quench of the FAM-labeled ssDNA indicates the existence of sufficient interaction to hold ssDNA inside the pores of these MOFs, and this allows for accurate quantification of ssDNA uptake as reflected by the exact fluorescence decay of the ssDNA solution (Fig. 3b). The presence of ssDNA inside the pores of Ni-IRMOF-74-II was further corroborated by the change in peak intensity in PXRD patterns of ssDNA-loaded Ni-IRMOF-74-II samples in comparison to MOFs with empty pores (Fig. 2c–f). In order to track the position and provide extra contrast in the electron density of ssDNA inside the MOF pores, Ni(II) cations were added to the solution to label the backbone of the DNA structure^33^. The electron density map of ssDNA confined in the pores was generated from 396 (41 independent) reflections, using difference Fourier analysis of the measured intensity profile of the ssDNA-loaded Ni-IRMOF-74-II samples subtracted by the calculated intensity profile of solvent containing Ni-IRMOF-74-II as background (Fig. 2g and Supplementary Figure 62 and 63). Although the exact atomic position of ssDNA was not achieved due to the disorder of these molecules and solvents, the electron density map provided enough resolution to reveal the orientation and distribution of confined ssDNA in the MOF pores (Fig. 2a,c–f), except for the electron density at the symmetry point of the pore center, which might be slightly induced by termination effect in the Fourier synthesis^34^. The ssDNA with helical morphology was well-accommodated inside the 1D pores of Ni-IRMOF-74-II as shown in the electron density maps curling along the walls of this MOF (Fig. 2g and Supplementary Figure 63). Large portion of the electron density was found close to the wall of the MOFs, while absence of electron density at the open metal site. This indicates that ssDNA interacts preferably with the organic linkers rather than metals in the MOF backbone. PXRD patterns of MOFs with different ssDNA uptakes were collected (2.3%, 4.7%, and 6.9% in weight, respectively, Fig. 2c–g and Supplementary Figure 62). As the ssDNA uptake increased, progressive increase in electron density near the MOF wall was observed due to higher occupancy. When the uptake approached saturation at 6.9% by weight, the electron density stretches closer to the walls as a combined result of the close packing of ssDNA in the MOF pores and their stronger interaction with the framework.

### Uptake, protection, and release of ssDNA

Here, we use an ssDNA of 33 nucleotides^35–37^, part of which is complementary to a certain sequence in the EGR-1 mRNA to illustrate three critical and consecutive steps in vitro, uptake, protection, and release, that mimics the intracellular delivery process (Fig. 3a). The three processes in vitro are carried out separately to assess the efficiency for each process. During the uptake process, all four members in this MOFs series (Ni-IRMOF-74-I, III, IV, and V) can include ssDNA into their pores and completely take them up from their solution within 1 h, as confirmed by negligible fluorescence remaining in the rest of the solution (Fig. 3b). In the control experiment using Ni-MOF-74, UiO-66, and UiO-67 with micropores^19^ (Supplementary Figure 14, 15, and 78), the entering of ssDNA is blocked due to pore size limitation. Only a small amount of ssDNA was absorbed on the exterior surface of these MOFs (Fig. 3b and Supplementary Figure 78). To further confirm whether the entire ssDNA was properly contained inside the pores of Ni-IRMOF-74 series rather than being partially entangled, an inclusion test was performed using ssDNA with both ends labeled. One end is labeled with FAM and the other end is labeled with 6-carboxytetramethylrhodamine (TAMRA) which gives a distinct red fluorescence at the wavelength of 580 nm (Supplementary Figure 58). When Ni-IRMOF-74-II samples were immersed into the solution of these doubly labeled ssDNA, concurrent decay of fluorescence at both wavelengths was observed in the solution, indicating the proper accommodation of the entire molecule into the MOF pores (Supplementary Figure 59).

The protection of ssDNA is assessed by immersing ssDNA loaded MOFs into the solution of 10% fetal bovine serum (FBS) that mimics the physiological environment (Fig. 3a). Without protection, DNA will be degraded into shorter pieces in the presence of nuclease in the FBS solution. All four members in the Ni-IRMOF-74 series (II–V) offer superior protection of ssDNA against degradation as evidenced by the survival of 95% of the DNA after 24-h immersion in FBS solution (Fig. 3c, Table 1 and Supplementary Figure 89). Control experiments using UiO-66, UiO-67, and mesoporous silica (SBA-15) (Supplementary Figure 16) as vectors were also performed, where they exhibited limited protection as the amount of survival ssDNA are lower than 7%. When MIL-101-Cr (Supplementary Figure 13) was tested, 94% residual preserved inside pores; however, as shown in the release test later, the included DNA did not exit the pores due to the strong host–guest interaction.

**Table 1:** Summary of the in vitro tests and transfection efficiency in cells using different vector materials

Non-viral vectors	Saturated concentration of ssDNA (wt%)^a^	Survived portion in serum (%)	Release efficiency (%)	Efficiency in RAW264.7 (%)	Efficiency in THP-1 (%)	Efficiency in CD4+ T (%)	Efficiency in B cell (%)
Ni-IRMOF-74-II	6.9	95	55	81	2	61	68
Ni-IRMOF-74-III	6.9	95	25	80	30	92	89
UiO-66	1.5	7	0	NA	NA	NA	NA
UiO-67	1.3	7	0	14	7	NA	NA
MIL-101	5.8	3	0	12	7	NA	NA
SBA-15	2.6	7	33	NA	NA	NA	NA
Lipo	0.02	88	0	30	6	58	73
Neofect	0.1	100	24	40	11	50	75

*NA* not applicable

^a^Weight percentage is used here to provide fair comparison due to the lack of specific concentration in the commercial Lipo and Neofect agents

### Release of ssDNA

The release of the included ssDNA was carried out by immersing MOFs in the solution of cDNA, quantitatively analyzed based on the recovery of fluorescence in the solution originated from the labeled ssDNA (Fig. 3a, d). The release of ssDNA from MOF pores is a dynamic equilibrium process evidenced by the gradual recovery of fluorescence as the time increases. After 4.5 h of immersion, Ni-IRMOF-74-II released 55.0% of the loaded ssDNA into the solution, highest among the Ni-IRMOF-74 series, while Ni-IRMOF-74-III to -V, UiO-66, UiO-67, MIL-101-Cr offer less release, 24.7%, 17.8%, 20.2%, 0%, 10.8%, and 9.9%, respectively (Fig. 3d and Supplementary Figure 88). The high performance of Ni-IRMOF-74-II in uptake, protection, and release tests demonstrated that mild interactions between ssDNA and pores are ideal for the design of host for intracellular delivery process (Table 1 and Supplementary Table 13).

### Investigation of the host–guest interactions

To figure out the type of interactions between ssDNA and Ni-IRMOF-74, five possible types of interactions were investigated including coordination, electrostatic forces, hydrogen bonds, π–π interactions, and van der Waals interactions. First, X-ray absorption spectra (XAS) and X-ray photoelectron spectroscopy (XPS) were tested on Ni-IRMOF-74-II before and after the uptake of ssDNA. The unaltered spectrums rule out the possible coordination between ssDNA with metal site in MOFs (Fig. 3e and Supplementary Figure 91–96). Second, the zeta potential was tested for the Ni-IRMOF-74-II series and ssDNA in aqueous solution; both are negative, thus the electrostatic interactions are unlikely to be the main force to hold ssDNA in the MOF pore (Supplementary Figure 97 and Supplementary Table 14). Third, the heating test at 95 °C was designed to explore the contribution of hydrogen bonding since hydrogen bonds could be broken at 95 °C (Fig. 3f). Almost no ssDNA release was observed in the test of Ni-IRMOF-74-III to -V showing the absence of hydrogen bond. To further rule out the effect of hydrogen bonds in Ni-IRMOF-74-II, a control experiment was carried out using the mixture of Ni salt and link (II) in equivalent amount with the corresponding MOF and under identical test condition applied for Ni-IRMOF-74-II, almost no fluorescence quenching was observed for the FAM-labeled ssDNA in 5 h (Fig. 3g and Supplementary Figure 79). If there is hydrogen bonding between ssDNA and links, there would be fluorescence quenching on both of the mixed complexes and Ni-IRMOF-74-II. But the little fluorescence quenching in mixed complexes suggests that pore restrictive effect may count for the high uptake rather than hydrogen bonding between the host and guest molecules in the case of Ni-IRMOF-74-II. Last but not least, we used TAMRA, a dye with conjugate structure, as a good indicator for the study of possible π–π interactions. The little uptake of TAMRA in Ni-IRMOF-74-II demonstrates that the impact of π–π interactions is also negligible. As the length of MOF linker increases from Ni-IRMOF-74-II to -V, the inclusion of TAMRA increases, demonstrating the strengthening of the π–π interactions as the number of phenylene units increase in the MOF linker (Supplementary Figure 58); while in the control experiments using FAM, a non-conjugate dye, there is no noticeable inclusion observed in any of these MOFs (Supplementary Figure 58). According to the experiments above, coordination, electrostatic forces, H-bonding, and π–π interactions are unlikely to be the main force to hold ssDNA in Ni-IRMOF-74-II, thus suggesting van der Waals interactions offered by suitable pore size and moderate accommodation in Ni-IRMOF-74-II are responsible for the uptake and release of ssDNA stands out of Ni-IRMOF-74 series (Supplementary Table 13). And it is in accordance with reported theoretical calculation of protein encapsulation in Mg-IRMOF-74 series, in which van der Waals interaction was proposed as the driving force for the inclusion^38^. The interactions between ssDNA and Ni-IRMOF-74 samples are strong enough to keep ssDNA inside the pore, but weaker than the H-bond to form dsDNA in the presence of cDNA, thereby allow for ssDNA release from the pores on demand.

### Intracellular delivery

Intracellular delivery of biomolecules is essential in biological research and therapeutic applications and gene editing^39–44^. One of the major obstacles for their clinical application is the lack of safe, efficient and low-cost delivery methods^39,40^, especially for stem cells and immune cells, which are the most difficult to transfect but also the most exciting target cell types in gene therapy^39,42,43^. Despite high delivery efficiency, existing viral method suffers from safety concern, high cost, and scale-up difficulty^39,40^. On the other hand, the conventional non-viral methods such as liposomes and cationic polymers and nanoparticles lack of precision for the inclusion of DNA, thus they exhibit limited transfection efficiency and relatively high cytotoxicity^39,40^ (Supplementary Figure 99–108). With the pore geometry well-matched with DNA, the pore size precisely controlled, and linkers designed based on aspirin derivatives, the MOFs reported here stand out as a new class of non-viral transfection vectors.

**Fig. 4:**
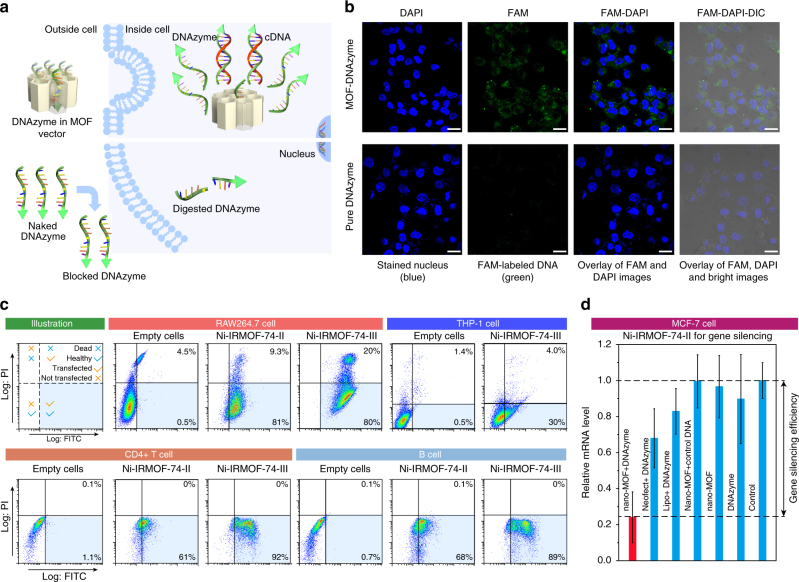
Effective intracellular delivery and gene silencing. **a** Illustration of intracellular delivery of ssDNA with and without MOF as vectors, above and below, respectively. **b** Comparison of the intracellular delivery efficiency of ssDNA with and without MOFs by confocal Laser Scanning Microscopy in MCF-7 cells. Nuclei were stained with DAPI (blue), FAM-labeled ssDNA in green, scale bar: 20 μm. **c** Transfection efficiency and toxicity of Ni-IRMOF-74-II and -III reflected in Flow cytometry in mouse immune cell (RAW264.7), human immune cell (THP-1), CD4+ T cell, and B cell. **d** Gene silencing efficiency of the DNAzyme carried by Ni-IRMOF-74-II, Lipo, and Neofect in MCF-7 cells (*n* = 3 technical replicates; bars represent mean ± s.d.)

In order to test the practical intracellular delivery of ssDNA using MOF vectors, five cells were used, including four mammalian immune cells that are extremely difficult to transfect by non-viral vectors^40^, two primary mouse immune cells (CD4+ T cells and B cells) and two macrophages (RAW264.7 from mouse and THP-1 from human). In addition, human breast cancer cell, MCF-7, was used to demonstrate gene silencing efficiency; 33-nucleotide ssDNA labeled with FAM fluorophores were used here as the cargo. Nanocrystals of four MOFs (Ni-IRMOF-74-II, -III, UiO-67, and MIL-101-Cr) were synthesized to favor endocytosis into cells (Supplementary Figure 45, 47–49)^16,45^. In good accordance to the in vitro tests, ssDNA loaded in IRMOF-74-II and -III were well-protected before entering the cells and then released in sufficient amount to pair up with cDNA in cytoplasm environment. Both Ni-IRMOF-74-II and -III demonstrate excellent transfection efficiency for RAW264.7 in comparison to empty cells, 90% and 100% of the cells were transfected in 2 h, respectively, revealed by the single channel of Flow cytometry detecting the FAM signal by FITC channel (Fig. 4c and Supplementary Figure 102). Another channel, propidium iodide (PI), was used to discriminate dead cells from live cells, thus reflecting the cytotoxicity of vectors^46^. The transfection efficiency for Ni-IRMOF-74-II and -III are 81% and 80%, respectively, much higher than that of the Neofect (40%) and Lipo (30%), thus these MOFs are applicable for the rat immune cell transfection (Fig. 4c and Supplementary Figure 102). It is worth noting that although both Lipo and Neofect give nearly 50% increase of FTIC, a large portion of the transfected cells are dead, 17% and 9% respectively, according to the increase in PI intensity. In the control experiments of the other two MOFs, UiO-67 and MIL-101-Cr, their nanocrystals show poor performance with transfection efficiency of less than 20% in both RAW264.7 and THP-1, consistent with the in vitro test (Supplementary Figure 102). In the test of another macrophages cell line, THP-1, Lipo and Neofect exhibit severe death of cells (83% and 36%) with low efficiency of transfection, 6% and 11% respectively (Supplementary Figure 102) while Ni-IRMOF-74-III achieves more than 30% transfection and gives negligible damage (4%) to cells based on the fluorescence intensity of FITC and PI (Fig. 4c and Supplementary Figure 102). Considering both the efficiency and cytotoxicity in transfection, Ni-IRMOF-74-II exhibits the best performance among all these vectors for RAW264.7, and Ni-IRMOF-74-III is the most effective one for THP-1. In order to eliminate the phagocytosis of special cell lines and extend the applicability to a wide range of cell types, transfection efficiency in the primary mouse immune cells (CD4+ T cells and B cells) was tested (Fig. 4c and Supplementary Figure 103). In CD4+ T cells, Ni-IRMOF-74-II and -III stand out with excellent transfection efficiency, 61% and 92% respectively. Both of them are better than the commercial agent, Lipo (58%) and Neofect (50%). High transfection efficiency in B cell was also observed when Ni-IRMOF-74-II and -III were used (68% and 89%, respectively). In all the immune cell tests, Ni-IRMOF-74-II and -III stand out as the most efficient vector for ssDNA delivery.

Last but not least, Ni-IRMOF-74-II was used in gene silencing to further confirm its potential for intracellular delivery and biological applications. DNAzyme (a 33-nucleotide ssDNA) was particularly designed with sequence in complementary to part of target EGR-1 mRNA, which generally exists in the cytoplasm of normal cells but over-expressed in MCF-7 human breast cancer cells^35–37^. This ssDNA was successfully delivered into MCF-7 human breast cancer cells by nanocrystals of Ni-IRMOF-74-II (Fig. 4b and Supplementary Figure 99–101), in which it was released by pairing up with target mRNA (Supplementary Figure 101), thus inhibit the expression of EGR-1 gene with nearly 76% inhibition efficiency (Fig. 4d). In contrast, Lipo and Neofect inhibit merely 17% and 32%, respectively, of EGR-1 gene expression under identical conditions.

## Discussion

In this report, we specifically extended the molecular building units of a known MOF, Ni-MOF-74, and synthesized an isoreticular series of MOFs (Ni-IRMOF-74-II, -III, -IV, and -V) with progressively larger pore sizes. The useful properties of porous materials originate from the interactions of substrates with the interior of the pore environment. The strength of these interactions and the geometry of the pores dictate the kinetics of uptake and release, and the manner in which a substrate diffuses in and out of the pores, respectively. Accordingly, we used these MOFs for the delivery of ssDNA into cells that cannot be effectively transfected by conventional transfection agents, and found that the Ni-IRMOF-74-II and -III with the lowest strength of interaction between ssDNA and MOF pores showed a remarkable potential in intracellular delivery.

## Methods

### Organic linkers

The synthesis of organic linkers was developed based on previously reported conditions with modifications to increase the yield^26^ (Supplementary Figure 2 and 3). All linkers were synthesized in gram scale (Supplementary Method 1).

### Synthesis of Ni-IRMOF-74-II

Ni(NO_3_)_2_·6H_2_O (90 mg, 0.31 mmol) and link II (26 mg, 0.095 mmol) were dissolved in 9 mL mixture of DMF/water/ethanol (1:1:1) in a 20 mL vial. The solution was heated in an isothermal oven at 120 °C and allowed to react for 24 h to yield green crystals; the details of synthesis can be found in Supplementary Method 2.

### Synthesis of Ni-IRMOF-74-III

Ni(NO_3_)_2_·6H_2_O (90 mg, 0.31 mmol) and link II (36 mg, 0.095 mmol) were dissolved in 9 mL mixture of DMF/water/ethanol (1:1:1) in a 20-mL vial. The solution was heated in an isothermal oven at 120 °C and allowed to react for 24 h to yield green crystals; the details of synthesis can be found in Supplementary Method 2.

### Synthesis of Ni-IRMOF-74-IV

Ni(NO_3_)_2_·6H_2_O (30 mg, 0.1 mmol) and link IV (14.5 mg, 0.03 mmol) were dissolved in 1.71 mL mixture of DMF/water/ethanol (5:1:1) in a 4-mL vial. The solution was heated in an isothermal oven at 120 °C and allowed to react for 24 h to yield green crystals; the details of synthesis can be found in Supplementary Method 2.

### Synthesis of Ni-IRMOF-74-V

Ni(NO_3_)_2_·6H_2_O (30 mg, 0.1 mmol) and link V (17.6 mg, 0.03 mmol) were dissolved in 1.71 mL of DMF/water/ethanol (5:1:1) in a 4-mL vial. The solution was heated in an isothermal oven at 120 °C and allowed to react for 24 h to yield green crystals; the details of synthesis can be found in Supplementary Method 2.

### Sample activation of Ni-IRMOF-74 series

The as-synthesized samples of IRMOF-74 series were washed three times with DMF before immersion in dry ethanol for 3 days; during this exchange process, dry ethanol was refreshed three times to fully replace the DMF solvent bound to open metal sites. The resulting ethanol-exchanged samples were transferred as suspension to a quartz cell followed by solvent decantion. The ethanol in the pores and bound to the open metal sites was removed by evacuating the Ni-IRMOF-74 samples (10^−2^ Torr) at room temperature for 12 h followed by heating at 130 °C for 12 h at the rate of 1 °C in both the heating and cooling process; the experimental details can be found in Supplementary Method 3.

### Uptake of ssDNA

Ten milligrams of Ni-IRMOF-74-II was transferred from glovebox and immersed separately in 10 mL ssDNA solution with 2.5%, 5%, and 10%, in weight percent for 5 h at room temperature. The exact uptake of ssDNA in each MOF sample was measured, 2.3%, 4.7%, and 6.9 % in weight percent, respectively, by tracing fluorescence quench of the FAM label on the ssDNA. The ssDNA attached to the MOF surface was removed by washing sequentially with deionized water and ethanol for three times for each solvent. The solvent was then removed by vacuum for 24 h at room temperature without decomposition of ssDNA in the pores; the experimental details can be found in Supplementary Method 4.

### Powder X-ray diffraction patterns

PXRD data were collected using both synchrotron and lab-based instrument. The X-ray diffraction data were obtained at beamline BL14B1 of the Shanghai Synchrotron Radiation Facility (SSRF) with a wavelength of 1.23823 Å. The detailed information about beamline BL14B1 can be found in refs. ^47,48^. Data collected on Rigaku Smartlab, a 9-KW lab-based diffractometer was operated at 45 kV, 200 mA using Cu K*α* (*λ* = 1.5406 Å) with a scan speed of 1°/min and a step size of 0.01° in 2*θ* at ambient temperature and pressure. Simulated PXRD patterns were calculated using software Mercury 3.0 from the refined crystal structure based on synchrotron data.

### Cells and media

MCF-7 (GDC055, CCTCC) cells and RAW264.7 (GDC143, CCTCC) cells were obtained from CCTCC. THP-1 (TIB-202, ATCC) cells were obtained from ATCC. The primary mouse immune cells (CD4+ T cells and B cells) were isolated from mouse. CD4+ T cells were isolated from mouse splenocytes through immunomagnetic negative selection using EasySep™ Mouse CD4+ T Cell Isolation Kit. Untouched B cells were isolated from mouse spleen cell suspensions using the B Cell Isolation Kit, an LS Column, and a MidiMACS™ Separator. THP-1 cells were cultured in RPMI-1640 medium (SH30809.01, HyClone, Inc.) supplemented with 1% penicillin/streptomycin (BL505A, Biosharp, Inc.) and 10% FBS (11011-8611, Every Green, Inc.). All the other cells were cultured in DMEM medium with the same supplements as for complete RPMI-1640 medium.

### Flow cytometry

Cells were seeded on a 24-well plate at the concentration of 1 × 10^5^ cells per well and cultured for 24 h. ssDNA-loaded vectors was diluted in 500 µL culture medium, mixed up, and incubated with cells for a predetermined time. Cells were washed with phosphate buffer saline (PBS) before they were harvested by trypsin-EDTA, and then rinsed again with PBS. About 200 μL PI (50 µg/mL in PBS) solution was incubated with each cell in the dark for 10 min at room temperature. After centrifugation and removal of the supernatant, cells were washed with PBS for three times. A flow cytometer (CyAn ADP, Beckman Coulter) was used to measure their fluorescence emission quantitatively. The empty cell without addition of ssDNA was used as control. The experimental details can be found in Supplementary Method 7.

### Confocal laser scanning microscopy (CLSM) for MCF-7 cells

The confocal images were collected by using a laser scanning confocal microscope (Leica TCS SP2, Germany). MCF-7 cells were incubated with FAM-labled DNAzyme (100 nM, the sequence is FAM-CCGCGGCCAGGCTAGCTACAACGACCTGGACGA) using nano-sized Ni-IRMOF-74-II as a vector. Naked FAM-DNAzyme (100 nM) was used in control experiments. The cells were incubated with DNAzyme for 3 h at 37 °C, followed by washing with PBS three times and continued to develop for another 3 h with fresh medium at 37 °C. These samples were fixed by 4% paraformaldehyde for 20 min at 4 °C, then they were stained with DAPI (10 μg/mL) for 20 min at room temperature before taking images by CLSM. The experimental details can be found in Supplementary Method 7.

### Real-time PCR (RT-PCR) analysis of mRNA expression level in MCF-7 cells

MCF-7 cells were seeded on a 6-well plate at 1 × 10^6^ cells per well and cultured for 24 h. Nano-sized Ni-IRMOF-74 (nano-MOF) loaded with DNAzyme, nano-MOF with control DNA, pure nano-MOF, and naked DNAzyme were incubated with MCF-7 cells (concentration, nano-MOF, 144 μg/mL, DNAzyme and control DNA, 300 nM, and MgCl_2_, 10 mM, respectively). After incubation for 48 h, cells were washed with PBS and extract total RNA by Trizol. mRNA was reverse transcribed by PrimeScript™ RT reagent Kit with gDNA Eraser (Takara). Quantitative RT-PCR analysis was performed using the SYBR^**®**^ Premix Ex Taq^**™**^ (Tli RNaseH Plus). The sequences of PCR primers are listed in Supplementary Table 11. Amplification was performed on CFX96^TM^ Real-Time System.

### Data availability

Crystallographic data that support the findings of this study have been deposited at the Cambridge Crystallographic Data Centre, under the deposition numbers 1562574 (Ni-IRMOF-74-II), 1562622 (Ni-IRMOF-74-III), 1562631 (Ni-IRMOF-74-IV), and 1562632 (Ni-IRMOF-74-V). All other data supporting the findings of this study are available within the Article and its Supplementary Information, or from the corresponding author upon reasonable request.

## Electronic supplementary material

Supplementary Information(PDF 5841 kb)
