# Supplementary material for: Metal-organic frameworks for precise inclusion of single-stranded DNA and transfection in immune cells
Source: Nat Commun. 2018 Apr 3;9:1293. doi: 10.1038/s41467-018-03650-w (PMC5882967; doi:10.1038/s41467-018-03650-w)
Supplement: Supplementary file 1 — Supplementary Information(PDF 5841 kb) [file 41467_2018_3650_MOESM1_ESM.pdf]

SUPPLEMENTARY INFORMATION

Metal-Organic Frameworks for Precise Inclusion of Single Stranded DNA and Transfection in Immune Cells – Peng *et al.*

This file includes:

Supplementary Text

Supplementary Figure 1 to 108

Supplementary Table 1 to 14

Supplementary References

Design of MOFs **3**

Supplementary Method 1. **4-13**

Synthesis of Organic Linkers

Supplementary Method 2. **14-47**

Synthesis and Structural Characterization of Ni-IRMOF-74 Series and MOF Nano-crystals

Supplementary Method 3. **48-60**

Characterization of ssDNA Loaded MOFs

Supplementary Method 4. **61-73**

Quantification of the Uptake and Release of ssDNA using MOF as Vectors through Fluorescence Measurement

Supplementary Method 5. **74-78**

Comparison of ssDNA Protection Performance using Various Non-viral Vectors by Gel Electrophoresis

Supplementary Method 6. **79-85**

Investigation of Interactions between ssDNA and MOF Pores

Supplementary Method 7. **86-92**

ssDNA Transfection Efficiency Revealed by Confocal Laser Scanning Microscopy and Flow Cytometry

Supplementary Method 8. **93-96**

Cytotoxicity Assessment of Ni-IRMOF-74 Series through Cell Proliferation Assays

Supplementary References **97-98**

Design of MOFs

Isoreticular extension was used in the design of these series of MOFs. **etb** topology of the original MOF-74 structure is well maintained when the size of organic linker is increased (Supplementary Figure 1). There are three considerations when we chose the topology of MOF-74 for the accommodation of DNA strands: (1) One-dimensional pores allow for the uptake and release of linear molecules with maximum contact. The pore sizes of IRMOF-74 series could be tuned without changing the underlying **etb** topology; (2) Precise control of pore size could be exercised to fine-tune the interaction between guest DNA molecules and the framework; (3) When Ni secondary building units (SBUs) were applied, the chemical stability of IRMOF-74 is significantly improved.

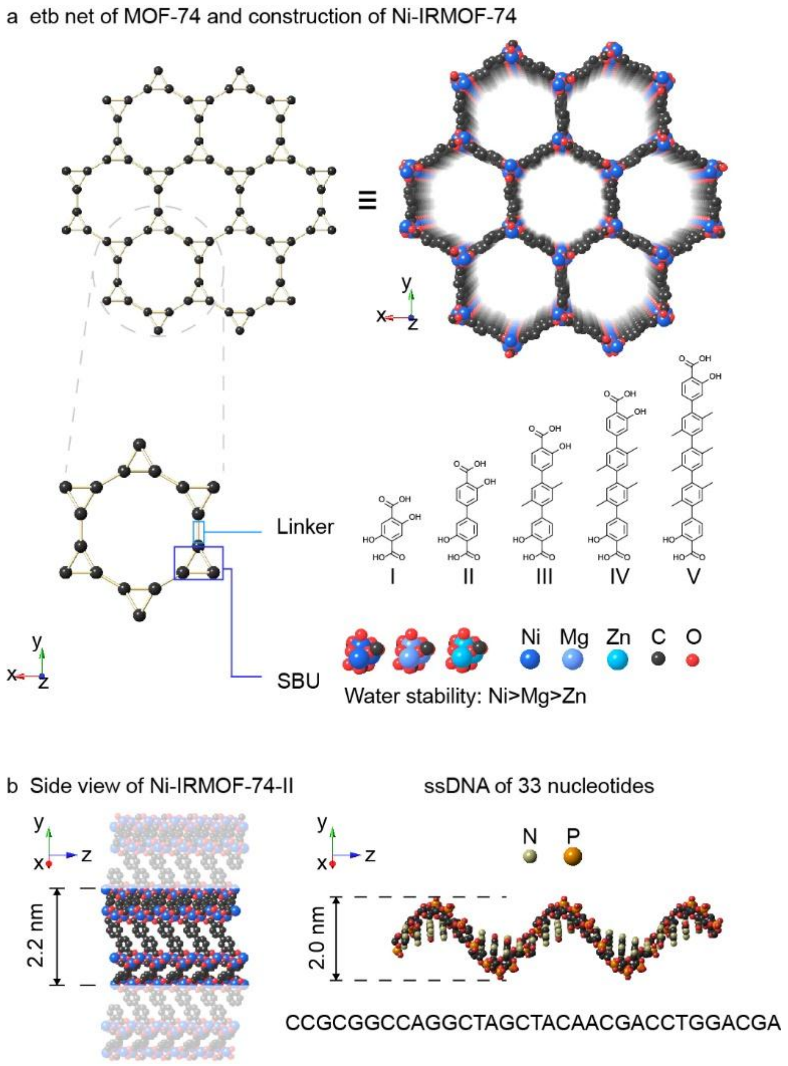

Supplementary Figure 1 Isoreticular extension of MOFs with etb topology a) Linker extension and comparison of various SBUs. b) Side view of the MOF reveals the size match between Ni-MOF-74-II and ssDNA. C, O, Ni, N and P atoms are shown in black, red, blue, grey and orange respectively.

Supplementary Method 1.

Synthesis of Organic Linkers

Chemicals

N, *N*-dimethylformamide (DMF), thionyl chloride, anhydrous sodium carbonate, methanol, ethanol, acetonitrile, *p*-dioxane, tetrafuran, sodium hydroxide, benzyl bromide, Pd/C (Pd=10%), nickel nitrate hexahydrate ($Ni(NO_3)_2$ $6H_2O$), hydrochloric acid (HCl) and sodium hydroxide (NaOH) were purchased from Sinopharm Chemical Reagent Co. Ltd (China). 4-bromo-2-hydroxylbenzoic acid (97%), Bis (pinacolato) diboron (98%) were purchased from Energy Chemical. Dichlorobis (triphenylphosphine) palladium (II) (98%) was purchased from Greenchem. 1,4-dibromo-2,5-dimethylbenzene (98%) was purchased from Bide Pharmatech. The deuterium reagent, $CDCl_3$ (D, 99.8%), $(CD_3)_2SO$ (D, 99.9%) were purchased from Sigma-Aldrich Chemical Co.(China). All chemicals were used without further purification unless otherwise mentioned.

Synthetic Strategy

The synthetic strategy to construct the linkers from II to V was developed based on previously reported method with modifications (-Br to replace -I) to reduce the cost and the modified route of linker IV avoid the use of 4,4'-diiodo-2,2',5,5'-tetramethyl-1,1'-biphenyl reducing the cost and increase the yield[1]. All linkers were based on palladium-catalyzed *Suzuki-Miyaura* cross-coupling reactions. The general strategy was to couple two equivalents of a terminal boronic ester part, whose carboxyl and hydroxyl groups has been transferred to methyl ester and benzylic ether to a dihalide intermediate. The *Suzuki-Miyaura* cross-coupling conditions, using CsF or K_2CO_3 as base with $Pd(dppf)Cl_2$ as the catalyst in a *p*-dioxane / H_2O (4:1 v/v) mixture, were found to be tolerant to the protection groups and also provide good yields. The deprotection started from the benzylic protection groups through hydrogenolysis followed by saponification to provide the target products, usually in quantitative yields over the two consecutive procedures without further purification other than filtration. The syntheses of linkers II to V are outlined in Supplementary Figure 2 and Supplementary Figure 3. Among all building blocks, the boronic ester **4** plays a key role and was scaled up to 30 grams. By coupling two equivalents of **4** with 1,4-dibromo-2,5-dimethylbenzene (**5**), the linker III was obtained after deprotection (Supplementary Figure 2). If one equivalent of the boronic ester **4** is coupled to an excess of the dihalide **5**, we shall get a monocoupled product, the building block for linker IV and V respectively. The halide of building block **8** was converted through a palladium-catalyzed coupling with pinacal diboron into the boronic ester **9** (Supplementary Figure 3). One equivalent of building block **8** and building **9** was coupled to provide precursor of linker IV. By coupling two

equivalents of boronic ester **9** with 1,4-dibromo-2,5-dimethylbenzene (**5**), the precursor of linker V was obtained through *Suzuki-Miyaura* cross-coupling. After hydrogenolysis and saponification, the linker IV and V were obtained respectively.

General Experimental Conditions

All reactions were carried out under an atmosphere of nitrogen in flame-dried flasks using anhydrous solvents, unless indicated otherwise. Thin-layer chromatography (TLC) was carried out using glass plates, pre-coated silica gel GF 254 containing fluorescent indicator (Sinopharm Chemical Reagent Co. Ltd (China)). The plates were inspected by UV light (254 nm). Column chromatography was carried out using the flash technique using silica-gel (200-300 mesh). ^{1}H and ^{13}C NMR spectra were recorded on a Bruker Advance III (400 MHz) spectrometer. The chemical shifts (δ) for ^{1}H spectra, given in ppm, are referenced to the residual proton signal of the deuterated solvent. The chemical shifts for ^{13}C spectra are referenced relative to the signal from the carbon of the deuterated solvent. Low-resolution mass spectra were collected on a Varian: 450GC,320MS (LR-EI). High-resolution mass spectra were collected on a LTQ Orbitrap Elite (Thermo-Fisher Scientific, Waltham, MA, USA) mass spectrometer (HR-ESI).

General Synthetic Procedures

Suzuki-Miyaura Cross-Couplings

A *p*-dioxane / H_2O mixture (4:1 v/v) was purged with Ar and transferred subsequently to a round-bottomed flask charged with the aryl halide (1.0 equiv), the boronic acid pinacol ester (3.0 equiv), CsF or K_2CO_3 (3.0 equiv) and Pd(dppf)Cl_2 (0.1 equiv). The resulting mixture was transferred to an oil bath pre-warmed to 90 ℃. And the reaction mixture was stirred vigorously for 24 hours. Then it was cooled down to room temperature (RT), and the products were purified noted as below.

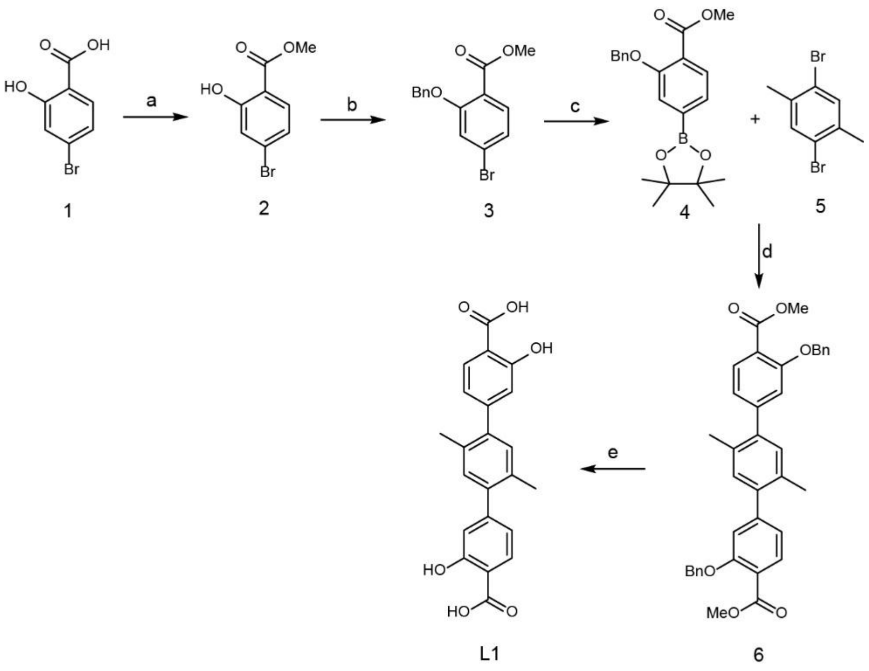

Supplementary Figure 2 Synthesis of linker III a) $SOCl_2$, MeOH, 75 °C. b) Na_2CO_3, BnBr, MeCN, 75 °C. c) $(Bpin)_2$, KOAc, Pd(dppf)Cl_2, 85 °C. d) CsF, Pd(dppf)Cl_2, *p*-dioxane/H_2O, 85 °C. e) Pd/C, H_2, THF/MeOH, 50 °C; NaOH, H_2O/THF/MeOH, 85 °C.

Compound 2

5 mL $SOCl_2$ was added dropwise to a solution of 4-bromo-2-hydroxybenzoic acid (**1**) (5 g, 23 mmol) in MeOH (39.6 g, 1.24 mol) in an ice bath. After the addition of $SOCl_2$, the ice bath was replaced by an oil bath. The reaction was then heated to 75 ℃ and stirred overnight. The reaction mixture was cooled to RT, before being extracted with EtOAc and H_2O. The aqueous phase was washed twice with EtOAc. The combined organic phases were dried (Na_2SO_4), filtrated and evaporated. The crude product was absorbed on silica-gel and subjected to column chromatography using hexane/EtOAc (10:1 v/v) as eluent. The purified product was obtained in white solid of 4.8 g (90.2% isolated yield). ^{1}H NMR (400 MHz, $CDCl_3$) δ (ppm) = 10.83 (s, 1 H), 7.67 (d, J = 8.5 Hz, 1 H), 7.17 (s, 1 H), 7.01 (d, J = 8.5 Hz, 1 H), 3.94 (s, 3 H). ^{13}C NMR (101 MHz, $CDCl_3$) δ (ppm) = 170.1, 161.9, 130.9, 129.9, 122.7, 120.8, 111.3, 52.5. LRMS (EI) calcd. For $C_8H_7BrO_3$ $[M]^+$: m/z = 230.0; Found: 230.0.

Compound 3

Na_2CO_3 (4.15 g, 39 mmol) was added to a solution of methyl 4-bromo-2-hydroxybenzoate (**2**) (3 g, 13 mmol) in MeCN (150 mL) at RT. Benzyl bromide (4.6 mL, 0.039 mmol) was added via syringe, and the resulting reaction mixture was warmed to 80 °C and stirred overnight. The reaction mixture was then cooled to RT and filtered subsequently to remove insoluble salts, which were extracted with EtOAc and H_2O. The aqueous phase was washed twice with EtOAc. The combined organic phases

were dried (Na_2SO_4), filtrated and evaporated. The crude product was absorbed on silica-gel and subjected to column chromatography using hexane/EtOAc (400:1, v/v) as eluent. The purified product was obtained in white solid of 3.14 g (75.3 % isolated yield). ^{1}H NMR (400 MHz, $CDCl_3$) δ (ppm)= 7.72 (d, J = 8.3 Hz, 1 H), 7.49 (d, J = 7.3 Hz, 2 H), 7.40 (dd, J = 8.1, 6.7 Hz, 2 H), 7.33 (t, J = 7.3 Hz, 1 H), 7.18 (d, J = 1.7 Hz, 1 H), 7.15 (dd, J = 8.3, 1.7 Hz, 1 H), 5.16 (s, 2 H), 3.89 (s, 3 H). ^{13}C NMR (101 MHz, $CDCl_3$) δ (ppm)= 165.9, 158.6, 136.0, 133.0, 128.6, 128.0, 127.6, 126.8, 123.8, 119.4, 117.2, 70.8, 52.1. HRMS (ESI) calcd. For $C_{15}H_{14}BrO_3$: m/z = 321.0 ($[M+H]^+$); Found m/z = 321.0.

Compound 4

Anhydrous DMF (100 mL) was purged with N_2 and then transferred via a cannula into a one-neck round bottomed flask charged with **3** (3 g, 9.4 mmol) and bis(pinacolato)diboron (3.57 g, 14.1 mmol). Potassium acetate (2.76 g, 28.1 mmol) and Pd(dppf)Cl_2 (0.68 g, 0.94 mmol) were then quickly added into the flask. The resulting mixture was stirred vigorously and heated at 85 °C for 5 hours. The reaction mixture was then cooled to RT and filtered subsequently to remove insoluble salts, which were extracted with EtOAc and H_2O. The aqueous solution was washed twice with EtOAc. The combined organic phases were dried (Na_2SO_4), filtrated and evaporated. The crude product was absorbed on silica-gel and subjected to column chromatography using hexane/EtOAc (25 :1 v/v) as eluent. The purified product was obtained in white solid of 3.0 g (87.2% isolated yield). ^{1}H NMR (400 MHz, $CDCl_3$) δ (ppm)= 7.84 (d, J = 7.6 Hz, 1 H), 7.57 (d, J = 7.4 Hz, 2 H), 7.52 - 7.46 (m, 2 H), 7.43 (t, J = 7.5 Hz, 2 H), 7.35 (t, J = 7.3 Hz, 1 H), 5.25 (s, 2 H), 3.93 (s, 3 H), 1.39 (s, 12 H). ^{13}C NMR (101 MHz, $CDCl_3$) δ (ppm) = 166.8, 157.3, 136.8, 130.9, 128.4, 127.7, 126.9, 126.9, 123.0, 119.2, 84.2, 70.5, 52.0, 24.8. HRMS (ESI) calcd. For $C_{21}H_{26}BO_5$: m/z = 369.1868 ($[M+H]^+$), found m/z = 369.1871.

Compound 6

A mixture of compound **4** (3 g, 8.15 mmol) and 1,4-dibromo-2,5-dimethylbenzene (0.6 g, 4.07 mmol) was dissolved in 100 mL mixed solvent of *p*-dioxane/H_2O (4:1 v/v). which was deoxygenated by three freeze-pump-thaw cycles and protected under N_2 atmosphere. After quick addition of CsF (2.44 g, 16.08 mmol) and Pd(dppf)Cl_2 (0.4 g, 0.54 mmol), the suspension was heated and stirred vigorously at 85 ℃ for 24 hours. The reaction mixture was then cooled to RT and filtered subsequently to remove insoluble salts, which were extracted with EtOAc and H_2O. The aqueous solution was washed twice with EtOAc. The combined organic phases were dried (Na_2SO_4), filtrated and evaporated. The crude product was absorbed on silica-gel and subjected to column chromatography using hexane/EtOAc (10 :1 v/v) as eluent. The purified product was

obtained in white solid of 3.85 g (80.6% isolated yield). ^{1}H NMR (400 MHz, $CDCl_3$) δ (ppm)= 7.90 (d, J = 7.7 Hz, 2 H), 7.50 (d, J = 7.3 Hz, 4 H), 7.40 (t, J = 7.5 Hz, 4 H), 7.29 (d, J = 22.2 Hz, 2 H), 7.09 (s, 2 H), 7.04 - 6.94 (m, 4 H), 5.23 (s, 4 H), 3.95 (s, 6 H), 2.15 (s, 6 H). ^{13}C NMR (101 MHz, $CDCl_3$) δ (ppm) = 166.7, 157.8, 146.9, 140.3, 136.6, 132.6, 131.7, 131.5, 128.6, 127.8, 126.8, 121.4, 119.1, 115.0, 70.6, 52.1, 19.7. HRMS (ESI) calcd. For $C_{38}H_{34}NaO_6$: m/z = 609.2 ($[M+Na]^+$); Found m/z = 609.2.

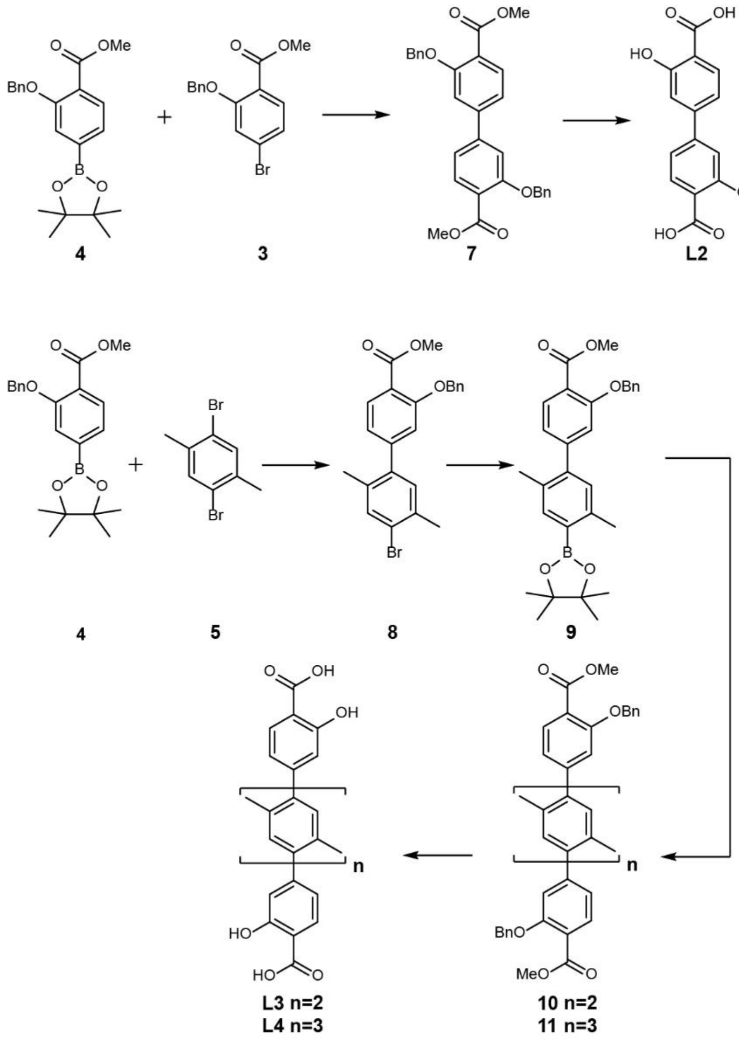

Supplementary Figure 3 Synthesis of linker II, linker III, linker IV and linker V the compound 1 to 4 is the same as Supplementary Figure 2. The method of deprotection for these linkers is the same as above.

Compound 7

A mixture of compound **3** (1 g, 3.11 mmol) and compound **4** (1.14 g, 3.11 mmol) was dissolved in 100 mL mixed solvent of *p*-dioxane/H_2O (4:1 v/v). which was deoxygenated by three freeze-pump-thaw cycles and protected under N_2 atmosphere. After quick adding of K_2CO_3 (3.0 g, 9.33 mmol) and Pd(dppf)Cl_2 (0.70 g, 0.31 mmol), the suspension was heated and stirred vigorously at 85 °C for 24 hours. The reaction mixture was then cooled to RT and filtered subsequently to remove insoluble salts, which were extracted with EtOAc and H_2O. The aqueous phase was washed twice with EtOAc. The combined organic phases were dried (Na_2SO_4), filtrated and evaporated. The crude product was absorbed on silica-gel and subjected to column chromatography using hexane/EtOAc (10 :1 v/v) as eluent. The purified product was obtained in white solid of 1.05 g (80.1% isolated yield). ^{1}H NMR (400 MHz, $CDCl_3$) δ (ppm) = 7.91 (d, J = 8.0 Hz, 2 H), 7.53 (d, J = 7.4 Hz, 4 H), 7.42 (dd, J = 10.2, 4.7 Hz, 4 H), 7.33 (dd, J = 8.4, 6.2 Hz, 2 H), 7.16 (dd, J = 8.0, 1.5 Hz, 2 H), 7.08 (d, J = 1.4 Hz, 2 H), 5.23 (s, 4H), 3.93 (s, 6 H). ^{13}C NMR (101 MHz, $CDCl_3$) δ (ppm) = 166.4, 158.4, 145.3, 136.6, 132.4, 128.6, 127.9, 126.9, 120.1, 119.4, 112.9, 70.8, 52.1. HRMS (ESI) calcd. For $C_{30}H_{27}BrO_6$: *m/z* = 483.2 ($[M+H]^+$); Found *m/z* = 483.2

Compound 8

A mixture of compound **4** (3 g, 8.15 mmol) and 1,4-dibromo-2,5-dimethylbenzene (10.7 g, 40.75mmol) was dissolved in 100 mL mixed solvent of *p*-dioxane/H_2O (4:1 v/v), which was deoxygenated by three freeze-pump-thaw cycles and protected under N_2 atmosphere. After quick adding of K_2CO_3 (2.59 g, 24.45 mmol) and Pd(dppf)Cl_2 (0.6 g, 0.81 mmol), the suspension was heated and stirred vigorously at 85 °C for 0.5 hours. The reaction mixture was then cooled to RT and filtered subsequently to remove insoluble salts, which were extracted with EtOAc and H_2O. The aqueous phase was washed twice with EtOAc. The combined organic solution was dried (Na_2SO_4), filtrated and evaporated. The crude product was absorbed on silica-gel and subjected to column chromatography using hexane/EtOAc (10 :1 v/v) as eluent. The purified product was obtained in white solid of 2.86 g (72.1% isolated yield). ^{1}H NMR (400 MHz, $CDCl_3$) δ (ppm) = 7.88 (d, J = 7.8 Hz, 1 H), 7.50 (d, J = 7.3 Hz, 2 H), 7.40 (dd, J = 15.9, 8.6 Hz, 3H), 7.31 (t, J = 7.3 Hz, 1 H), 7.04 (s, 1 H), 6.96 - 6.87 (m, 2 H), 5.21 (s, 2 H), 3.94 (s, 3 H), 2.39 (s, 3 H), 2.09 (s, 3 H). ^{13}C NMR (101 MHz, $CDCl_3$) δ (ppm) = 146.3, 139.89, 136.6, 135.1, 134.4, 133.9, 131.7, 131.4, 128.5, 127.8, 126.7, 124.1, 121.2, 119.2, 114.8, 70.5, 52.0, 22.3, 19.4. HRMS (ESI) calcd. For $C_{23}H_{22}BrO_3$: *m/z* = 425.1 ($[M+H]^+$); Found *m/z* = 425.1.

Compound 9

Anhydrous DMF (100 mL) was purged with N_2 and then transferred via a cannula into a one-neck round bottomed flask charged with **3** (3 g, 9.4 mmol) and bis(pinacolato)diboron (3.57 g, 14.1 mmol). Potassium acetate (2.76 g, 28.1 mmol) and Pd(dppf)Cl_2 (0.68 g, 0.94 mmol) were then quickly added into the flask. The resulting mixture was stirred vigorously and heated at 85 °C for 5 hours. The reaction mixture was then cooled to RT and filtered subsequently to remove insoluble salts, which were extracted with EtOAc and H_2O. The aqueous phase was washed twice with EtOAc. The combined organic phases were dried (Na_2SO_4), filtrated and evaporated. The crude product was absorbed on silica-gel and subjected to column chromatography using hexane/EtOAc (25 :1 v/v) as eluent. The purified product was obtained in white solid of 2.46 g (73.8% isolated yield). ^{1}H NMR (400 MHz, $CDCl_3$) δ (ppm) = 7.88 (d, J = 7.7 Hz, 1H), 7.67 (s, 1H), 7.49 (d, J = 7.4 Hz, 2H), 7.39 (t, J = 7.5 Hz, 2 H), 7.31 (t, J = 7.3 Hz, 1 H), 7.01 (s, 1 H), 6.97 - 6.91 (m, 2 H), 5.21 (s, 2 H), 3.94 (s, 3 H), 2.54 (s, 3 H), 2.12 (s, 3 H), 1.37 (s, 12 H), 1.27 (s, 1 H). ^{13}C NMR (101 MHz, $CDCl_3$) δ (ppm) = 166.67, 157.72, 147.38, 143.06, 142.32, 138.02, 136.68, 131.58, 131.23, 130.75, 128.52, 127.72, 126.76, 121.28, 118.91, 114.85, 83.47, 70.53, 52.01, 24.85, 21.63, 19.41. HRMS (ESI) calcd. For $C_{29}H_{34}BO_5$: m/z = 473.2 ([M+H]$^+$); Found m/z = 473.2.

Compound 10

A mixture of compound **8** (1 g, 2.35 mmol) and compound **9** (1.11 g, 2.35 mmol) was dissolved in 50 mL mixed solvent of *p*-dioxane/H_2O (4:1 v/v). which was deoxygenated by three freeze-pump-thaw cycles and protected under N_2 atmosphere. After quick addition of K_2CO_3 (2.27 g, 7.05 mmol) and Pd(dppf)Cl_2 (0.54 g, 0.24 mmol), the suspension was heated and stirred vigorously at 85 °C for 24 hours. The reaction mixture was then cooled to RT and filtered subsequently to remove insoluble salts, which were extracted with EtOAc and H_2O. The aqueous phase was washed twice with EtOAc. The combined organic phases were dried (Na_2SO_4), filtrated and evaporated. The crude product was absorbed on silica-gel and subjected to column chromatography using hexane/EtOAc (10 :1 v/v) as eluent. The purified product was obtained in white solid of 1.29 g (79.4% isolated yield) ^{1}H NMR (400 MHz, $CDCl_3$) δ (ppm) = 7.91 (d, J = 8.2 Hz, 2 H), 7.51 (m, 4 H), 7.41 (m, 4 H), 7.32 (m, 2 H), 7.10 (s, 2 H), 7.03 (dd, J = 6.3, 1.7 Hz, 6 H), 5.25 (s, 4 H), 3.95 (s, 6 H), 2.17 (s, 6 H), 2.12 (s, 6 H). ^{13}C NMR (101 MHz, $CDCl_3$) δ (ppm) = 166.7, 157.8, 147.4, 140.8, 139.6, 136.7, 133.3, 132.1, 131.6, 131.5, 130.8, 128.5, 127.8, 126.8, 121.60, 118.8, 115.0, 70.6, 52.1, 19.8, 19.4. HRMS (ESI) calcd. For $C_{46}H_{43}O_6$: m/z = 691.3 ([M+H]$^+$); Found m/z = 691.3.

Compound 11

A mixture of compound **5** (0.18 g, 0.71 mmol) and compound **9** (1.01 g, 2.12 mmol) was dissolved in 50 mL mixed solvent of *p*-dioxane/H_2O (4:1 v/v). which was deoxygenated by three freeze-pump-thaw cycles and protected under N_2 atmosphere. After quick addition of K_2CO_3 (0.67 g, 6.36 mmol) and Pd(dppf)Cl_2 (0.47 g, 0.21 mmol), the suspension was heated and stirred vigorously at 85 °C for 24 hours. The reaction mixture was then cooled to RT and filtered subsequently to remove insoluble salts, which were extracted with EtOAc and H_2O. The aqueous solution was washed twice with EtOAc. The combined organic solution was dried (Na_2SO_4), filtrated and evaporated. The crude product was absorbed on silica-gel and subjected to column chromatography using hexane/EtOAc (10 :1 v/v) as eluent. The purified product was obtained in white solid of 0.39 g (70.9% isolated yield). ^{1}H NMR (400 MHz, $CDCl_3$) δ (ppm) = 7.92 (d, J = 8.2 Hz, 2 H), 7.53 (d, J = 7.4 Hz, 4 H), 7.41 (t, J = 7.5 Hz, 4 H), 7.32 (t, J = 7.3 Hz, 2 H), 7.18 – 6.94 (m, 10H), 5.25 (s, 4 H), 3.95 (s, 6 H), 2.18 (d, J = 6.0 Hz, 6 H), 2.16 - 2.00 (m, 12 H). ^{13}C NMR (101 MHz, $CDCl_3$) δ (ppm) = 166.7, 157.8, 147.5, 141.24, 141.18, 139.98, 139.93, 139.45, 136.7, 133.38, 132.78, 132.68, 132.04, 132.03, 131.61, 131.54, 130.73, 130.67, 130.65, 128.5, 127.7, 126.8, 121.6, 118.9, 115.1, 70.6, 52.0, 19.78, 19.73, 19.44, 19.35, 19.28. HRMS (ESI) calcd. For $C_{29}H_{34}BO_5$: *m/z* = 795.4 ($[M+H]^+$); Found *m/z* = 795.4.

Linker II

Compound **7** (2 g, 4.15 mmol) was dissolved in 70 mL THF and 30 mL MeOH, added with 10% by weight Pd/C (0.98 g, 0.90 mmol). The reaction septum and the heterogenous solution was purged with H_2 that was delivered by a balloon atttached to an 8” stainless steel needle. A balloon inflated with H_2 was attached to the reaction vessel, which was transferred subsequently to an oil bath pre-warmed to 50 °C. The reaction mixture was stirred and detected through TLC until its complete reaction. The reaction mixture was transferred to a one-neck flask. Then 10 mL 2 M NaOH solution was added to the reaction mixture. The suspension was heated and stirred vigorously at 75 °C overnight. The mixture was filtered as soon as possible while it is hot. The filtrate was then cooled to RT and acidified with concentrated HCl until a pH < 2 was attained and the resulting precipitate was collected by vacuum filtration, washed with ample H_2O and dried at 65 °C for 24 h to provide the target compound as white powder (0.6 g, 63.6 % isolated yield). ^{1}H NMR (400 MHz, DMSO-*d*$_6$) δ (ppm) = 7.87 (d, J = 8.7 Hz, 2 H), 7.36 - 7.19 (m, 4 H). ^{13}C NMR (101MHz, DMSO-*d*$_6$) δ (ppm) = 171.7, 161.4, 145.6, 131.0, 118.0, 115.3, 112.8. HRMS (ESI) calcd. for $C_{14}H_{10}O_6$: *m/z* = 273.0 ($[M-H]^-$); Found *m/z* = 273.0.

Linker III

Compound **6** (1.5 g, 4.26 mmol) was dissolved in 70 mL THF and 30 mL MeOH, added with 10% by weight Pd/C (0.98 g, 0.90 mmol). The reaction septum and the heterogeneous solution were purged with H_2 that was delivered by a balloon attached to an 8” stainless steel needle. A balloon inflated with H_2 was attached to the reaction vessel, which was transferred subsequently to an oil bath pre-warmed to 50 °C. The reaction mixture was stirred and detected through TLC until its complete reaction. The reaction mixture was transferred to a one-neck flask. Then 10 mL 2 M NaOH solution was added to the reaction mixture. The suspension was heated and stirred vigorously at 75 °C overnight. The mixture was filtered as soon as possible while it is hot. The filtrate was then cooled to RT and acidified with concentrated HCl until a pH < 2 was attained and the resulting precipitate was collected by vacuum filtration, washed with ample H_2O and dried at 65 °C for 24 h to provide the target compound as white powder (0.6 g, 63.6 % isolated yield). ^{1}H NMR (400 MHz, DMSO-d_6) δ (ppm) = 7.86 (d, J = 8.5 Hz, 2 H), 7.19 (s, 2 H), 7.01 - 6.92 (m, 4 H), 2.25 (s, 6 H), ^{13}C NMR (101MHz, DMSO-d_6) δ (ppm) = 171.8, 160.9, 148.3, 139.7, 132.2, 131.3, 130.2, 120.3, 117.4, 111.7, 19.5. HRMS (ESI) calcd. For $C_{22}H_{17}O_6$: m/z = 377.1 ([M-H]$^-$); Found m/z = 377.1.

Linker IV

Compound **10** (1.5 g, 2.17 mmol) was dissolved in 70 mL THF and 30 mL MeOH, added with 10% by weight Pd/C (0.98 g, 0.90 mmol). The reaction septum and the heterogenous solution was purged with H_2 that was delivered by a balloon atttached to an 8” stainless steel needle. A balloon inflated with H_2 was attached to the reaction vessel, which was transferred subsequently to an oil bath pre-warmed to 50 °C. The reaction mixture was stirred and detected through TLC until its complete reaction. The reaction mixture was transferred to a one-neck flask. Then 10 mL 2 M NaOH solution was added to the reaction mixture. The suspension was heated and stirred vigorously at 75 °C overnight. The mixture was filtered as soon as possible while it is hot. The filtrate was then cooled to RT and acidified with concentrated HCl until a pH < 2 was attained and the resulting precipitate was collected by vacuum filtration, washed with ample H_2O and dried at 65 °C for 24 h to provide the target compound as white powder (0.8 g, 76.4 % isolated yield). ^{1}H NMR (400 MHz, DMSO-d_6) δ (ppm) = 11.38 (s, 2 H), 7.87 (d, J = 8.6 Hz, 2 H), 7.20 (s, 2 H), 7.07 (s, 2 H), 7.00 (d, J = 1.6 Hz, 1 H), 7.00 – 6.93 (m, 3 H), 2.26 (s, 6 H), 2.07 (s, 6 H). ^{13}C NMR (101MHz, DMSO-d_6) δ (ppm) = 171.9, 160.9, 148.5, 140.3, 139.0, 132.8, 131.8, 131.3, 130.6, 130.1, 120.3, 117.4, 111.8, 19.6, 19.1. HRMS (ESI) calcd. For $C_{30}H_{26}O_6$: m/z = 481.2 ([M-H]$^-$); Found m/z = 481.2.

Linker V

Compound **11** (1.5 g, 1.89 mmol) was dissolved in 70 mL THF and 30 mL MeOH, added with 10% by weight Pd/C (0.98 g, 0.90 mmol). The reaction septum and the heterogenous solution was purged with H_2 that was delivered by a balloon atttached to an 8” stainless steel needle. A balloon inflated with H_2 was attached to the reaction vessel, which was transferred subsequently to an oil bath pre-warmed to 50 °C. The reaction mixture was stirred and detected through TLC until its complete reaction. The reaction mixture was transferred to a one-neck flask. Then 10 mL 2 M NaOH solution was added to the reaction mixture. The suspension was heated and stirred vigorously at 75 °C overnight. The mixture was filtered as soon as possible while it is hot. The filtrate was then cooled to RT and acidified with concentrated HCl until a pH < 2 was attained and the resulting precipitate was collected by vacuum filtration, washed with ample H_2O and dried at 65 °C for 24 h to provide the target compound as white powder (0.9 g, 81.8 % isolated yield). ^1H NMR (400 MHz, DMSO-d_6) δ (ppm) = 7.87 (d, J = 8.6 Hz, 2 H), 7.21 (s, 2 H), 7.11 (d, J = 6.3 Hz, 2 H), 7.07 (d, J = 2.1 Hz, 2 H), 7.00 (dd, J = 6.7, 1.6 Hz, 4 H), 2.27 (s, 6 H), 2.08 (d, J = 6.8 Hz, 12 H). ^{13}C NMR (101MHz, DMSO-d_6) δ (ppm) = 171.9, 161.0, 148.6, 140.4, 139.0, 132.8, 131.8, 131.3, 130.6, 130.1, 120.4, 117.4, 111.6, 19.6, 19.1. HRMS (ESI) calcd. For $C_{38}H_{34}O_6$: *m/z* = 585.2 ([M-H]$^-$); Found *m/z* = 585.2.

Supplementary Method 2.

Synthesis and Structural Characterization of Ni-IRMOF-74 Series and MOF Nano-crystals

X-ray Crystallography

The PXRD data of Ni-IRMOF-74-II to-V was collected in transmission geometry in Synchrotron for structure refinements, while the PXRD data of the stability test were collected on a Rigaku Smartlab diffractometer (Cu Kα). Quartz capillaries with 0.6 mm diameter made by Hampton Research were used for data collection. The wavelength of Beamline 14B of the Shanghai Synchrotron Radiation Facility (SSRF) was tuned to 1.23823 Å (10 keV). Supplementary Table 1 shows the Rietveld refinement results of the lattice. Supplementary Tables 2 to 5 list the atomic parameters from Ni-IRMOF-74-II to Ni-IRMOF-74-V.

Water stability test

10 mg of activated Ni-IRMOF-74 was immersed in 5 mL deionized water for 24 hours before centrifugation. After centrifugation, the sample was washed by ethanol for three times and dried under vacuum (10^{-2} Torr) at room temperature for 12 h followed by heating at 130 °C for 12 h.

Elemental Analysis

Elemental analysis was performed on an Elementar Vario EL cube instrument using about 5 mg of sample every time.

Supplementary Table 1 Lattice parameters of Ni-IRMOF-74 crystals

Ni-IRMOF-74-	II	III	IV	V
Empirical Formula	$C_7H_4NiO_{10}$	$C_{22}H_{14}Ni_2O_{13}$	$C_{30}H_{22}Ni_2O_{10}$	$C_{38}H_{30}Ni_2O_9$
Space group	$R\bar{3}$	$R\bar{3}$	$R3$	$R3$
Z	18	18	9	9
a (Å)	36.03(5)	46.28(5)	56.2(2)	68.2(4)
c (Å)	6.964(12)	6.705(8)	6.57(4)	7.02(7)
R_p	7.22	5.82	4.05	4.73
R_{wp}	10.42	8.33	5.67	7.06

Z: numbers of molecules in one unit cell.

a and c: parameters of unit cell.

Synthesis of Ni-MOF-74:

Ni-MOF-74 was synthesized and activated according to the literature procedure[2-5]. The good agreement between the pattern of activated sample and that of the simulated model indicated the material has the same crystal structure as simulated one. The unaltered PXRD pattern of the activated sample after immersion in water for 24 hours demonstrates that Ni-MOF-74 is stable in water (Supplementary Figure 4). Anhydrous DMF and dry ethanol for the synthesis of MOFs are purchased from Sigma-Aldrich Chemical Co. (China). DMF used in the solvent exchange is dried with molecular sieve (4 Å).

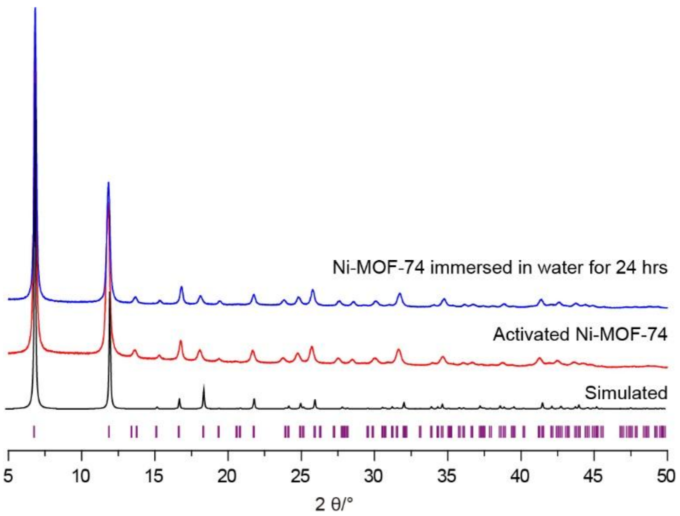

Supplementary Figure 4 Comparisons of the experimental PXRD pattern of activated Ni-MOF-74 (red), after immersion in water for 24 hours (blue) with the simulated diffraction pattern (black) (Cu Kα λ = 1.5406 Å).

Synthesis of Ni-IRMOF-74-II:

The patterns of Ni-IRMOF-74-II used for Rietveld refinements were collected at the beam line 14B of the Shanghai Synchrotron Radiation Facility (SSRF) using capillaries (diameter: 0.6 mm, quartz made by Hampton Research) with a wavelength of 1.23823 Å (Supplementary Figure 5). Supplementary Table 2 shows the results of lattice refinements.

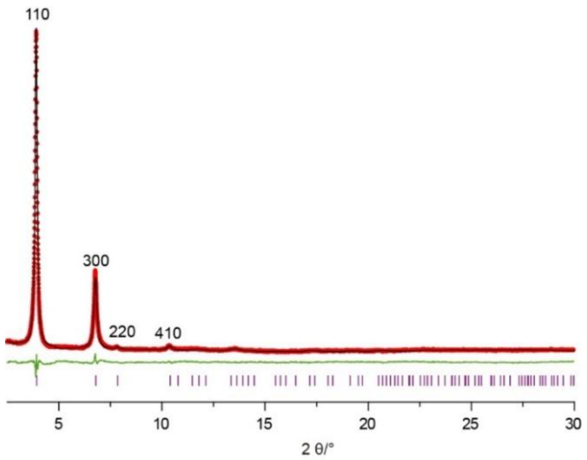

Supplementary Figure 5 X-ray diffraction patterns of Ni-IRMOF-74-II collected in Synchrotron (λ=1.23823 Å). The experimental (red), refined (black) and difference (green) patterns are displayed. The Bragg positions are marked as pink bars.

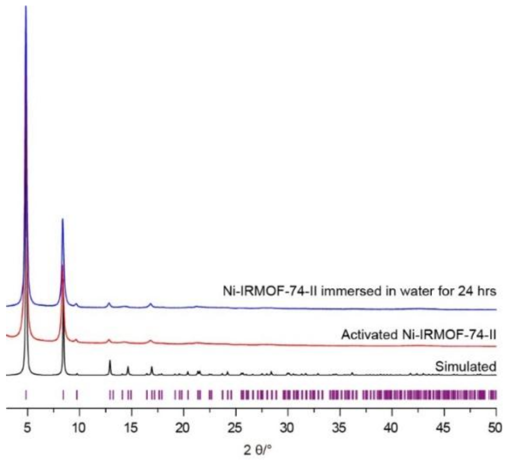

Supplementary Figure 6 Comparisons of the experimental PXRD pattern of activated Ni-IRMOF-74-II (red), after immersion in water for 24 hours (blue) with the simulated diffraction pattern (black) (Cu Kα λ = 1.5406 Å.

Supplementary Table 2 Atomic parameters of Ni-IRMOF-74-II

Atom	Occupancy	x	y	z	B_{iso} (Å^2)
Ni1	1.0	0.349(2)	0.322(3)	-0.15(2)	1
C1	1.0	0.344(2)	0.2850(17)	0.240(16)	3

C2	1.0	0.343(8)	0.252(4)	0.374(18)	3
C3	1.0	0.337(11)	0.254(5)	0.560(17)	3
C4	1.0	0.337(18)	0.221(9)	0.69(2)	3
C5	1.0	0.34(2)	0.187(11)	0.60(3)	3
C6	1.0	0.35(2)	0.188(11)	0.43(3)	3
C7	1.0	0.353(13)	0.221(8)	0.30(2)	3
O1	1.0	0.350(5)	0.282(2)	0.059(17)	3
O2	1.0	0.340(6)	0.316(3)	0.301(18)	3
O3	1.0	0.330(9)	0.287(4)	0.620(18)	3
O4	1.0	0.401303	0.336460	-0.223960	5
O5	1.0	0.343409	0.885634	0.705620	5
O6	1.0	0.423610	1.086659	0.120773	5
O7	1.0	0.429901	0.17402	0.819527	5
O8	1.0	0.789713	0.049415	0.876900	5
O9	1.0	0.773460	0.039060	0.781310	5
O10	1.0	0.500000	0.000000	0.500000	5

Synthesis of Ni-IRMOF-74-III:

The patterns of Ni-IRMOF-74-III used for Rietveld refinements were collected at the beam line 14B of the Shanghai Synchrotron Radiation Facility (SSRF) using capillaries (diameter: 0.6 mm, quartz made by Hampton Research) with a wavelength of 1.23823 Å (Supplementary Figure 7). Supplementary Table 3 shows the results of lattice refinements.

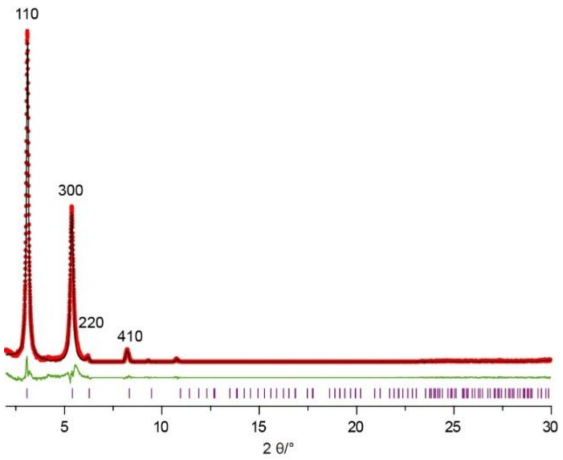

Supplementary Figure 7 X-ray diffraction patterns of Ni-IRMOF-74-III were collected in Synchrotron (λ=1.23823 Å). The experimental (red), refined (black) and difference (green) patterns are displayed. The Bragg positions are marked as pink bars.

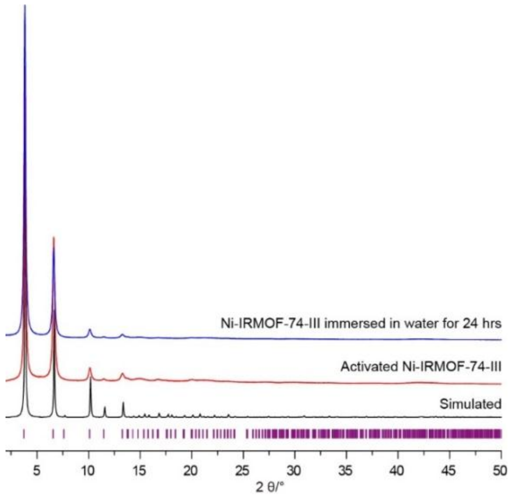

Supplementary Figure 8 Comparisons of the experimental PXRD pattern of activated Ni-IRMOF-74-III (red), after immersion in water for 24 hours (blue) with the simulated pattern (black) (Cu Kα λ = 1.5406 Å).

Supplementary Table 3 Atomic parameters of Ni-IRMOF-74-III

Atom	Occupancy	x	y	z	B_{iso} (Å^2)
C1	1.0	0.341600	0.296330	0.500900	1
C2	1.0	0.339480	0.270140	0.645900	1

C3	1.0	0.328810	0.267030	0.856480	1
C4	1.0	0.327030	0.241240	0.980520	1
C5	1.0	0.33690	0.219220	0.904200	1
C6	1.0	0.347940	0.222430	0.698260	1
C7	1.0	0.349270	0.247510	0.570970	1
C8	1.0	0.335600	0.192620	1.039470	1
C9	1.0	0.364900	0.193840	1.114240	1
C10	1.0	0.304390	0.165570	1.093310	1
C11	1.0	0.398850	0.223030	1.065600	1
Ni1	1.0	0.351260	0.32650	0.110510	1
O1	1.0	0.351460	0.296910	0.315620	1
O2	1.0	0.333660	0.317510	0.556660	1
O3	1.0	0.318600	0.287620	0.949300	1
O4	1.0	0.391370	0.331330	-0.014390	1
O5	1.0	0.300840	0.668030	0.062280	1
O6	1.0	0.473920	0.350250	0.573290	1
O7	1.0	0.364510	0.049500	0.916667	1
O8	1.0	0.652370	0.773430	0.750310	1

Synthesis of Ni-IRMOF-74-IV:

The patterns of Ni-IRMOF-74-IV used for Rietveld refinements were collected at the beam line 14B of the Shanghai Synchrotron Radiation Facility (SSRF) using capillaries (diameter: 0.6 mm, quartz made by Hampton Research) with a wavelength of 1.23823 Å (Supplementary Figure 9). Supplementary Table 4 shows the results of lattice refinements.

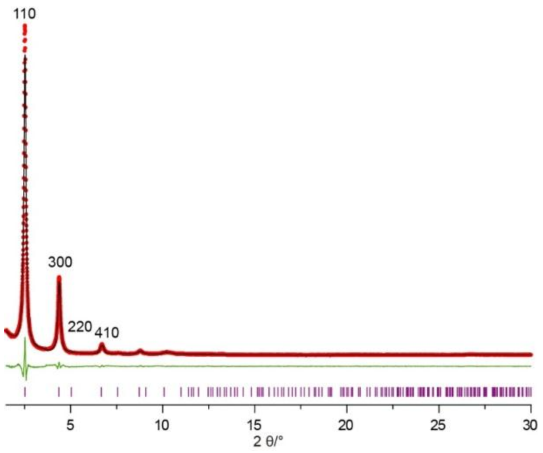

Supplementary Figure 9 X-ray diffraction patterns of Ni-IRMOF-74-IV were collected in Synchrotron (λ=1.23823 Å). The experimental (red), refined (black) and difference (green) patterns are displayed. The Bragg positions are marked as pink bars.

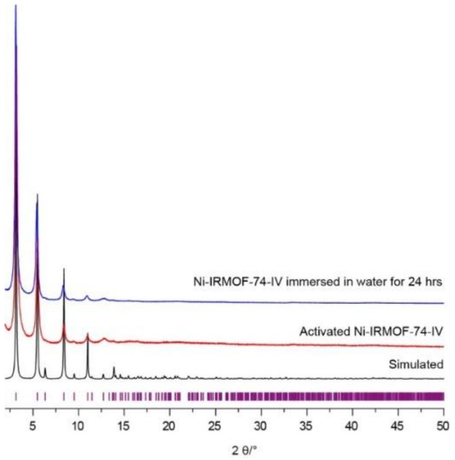

Supplementary Figure 10 Comparisons of the experimental PXRD pattern of activated Ni-IRMOF-74-IV (red), after immersion in water for 24 hours (blue) with the simulated diffraction pattern (black) (Cu Kα λ = 1.5406 Å).

Supplementary Table 4 Atomic parameters of Ni-IRMOF-74-IV

Atom	Occupancy	x	y	z	B_{iso} (Å^2)
C1	1.0	-0.108040	0.206710	0.327960	1
C2	1.0	-0.128040	0.186870	0.205120	1
C3	1.0	-0.156160	0.175840	0.24520	1
C4	1.0	-0.164060	0.185140	0.411980	1
C5	1.0	-0.144020	0.204930	0.535000	1
C6	1.0	-0.116040	0.215990	0.493350	1
C7	1.0	-0.25755	0.080540	-0.604360	1
C8	1.0	-0.237450	0.100250	-0.482260	1
C9	1.0	-0.237110	0.094960	-0.282530	1
C10	1.0	-0.25665	0.069320	-0.208720	1
C11	1.0	-0.055550	0.282940	0.663880	1
C12	1.0	-0.054680	0.277910	0.868110	1
C13	1.0	-0.032780	0.297150	1.006340	1
C14	1.0	-0.299980	0.035850	-0.670410	1
C15	1.0	-0.277940	0.054760	-0.531530	1
C16	1.0	-0.277130	0.049340	-0.328040	1
C17	1.0	-0.075860	0.262500	0.545810	1
C18	1.0	-0.095210	0.237070	0.623130	1
C19	1.0	-0.094860	0.232130	0.822630	1
C20	1.0	-0.074850	0.252190	0.942970	1
C21	1.0	-0.216270	0.115990	-0.149740	1
C22	1.0	-0.201240	0.108580	-0.024110	1
C23	1.0	-0.181940	0.127610	0.106740	1
C24	1.0	-0.176820	0.154900	0.110300	1
C25	1.0	-0.191740	0.162330	-0.016140	1
C26	1.0	-0.211820	0.143010	-0.141270	1
C27	1.0	-0.078390	0.217380	0.281590	1
C28	1.0	-0.193560	0.174150	0.463430	1
C29	1.0	-0.167930	0.117960	0.245280	1
C30	1.0	-0.229350	0.151270	-0.253650	1
Ni1	1.0	-0.007620	0.311040	1.397600	1
Ni2	1.0	-0.325080	0.022370	-1.062400	1

Atom	Occupancy	x	y	z	1
O1	1.0	-0.005780	0.278180	1.493850	1
O2	1.0	-0.038160	0.308020	0.572490	1
O3	1.0	-0.013360	0.320400	0.948910	1
O4	1.0	-0.033740	0.289820	1.184820	1
O5	1.0	-0.298760	0.043240	-0.849040	1
O6	1.0	-0.319800	0.012810	-0.613220	1
O7	1.0	-0.295010	0.024370	-0.237470	1
O8	1.0	0.673050	0.073540	-1.213110	1
O9	1.0	0.372663	0.338765	-0.19178	1
O10	1.0	0.425296	0.287185	-0.07242	1
O11	1.0	0.325814	0.251933	-0.42302	1

Synthesis of Ni-IRMOF-74-V:

The patterns of Ni-IRMOF-74-V used for Rietveld refinements were collected at the beam line 14B of the Shanghai Synchrotron Radiation Facility (SSRF) using capillaries (diameter: 0.6 mm, quartz made by Hampton Research) with a wavelength of 1.23823 Å (Supplementary Figure 11). Supplementary Table 5 shows the results of lattice refinement.

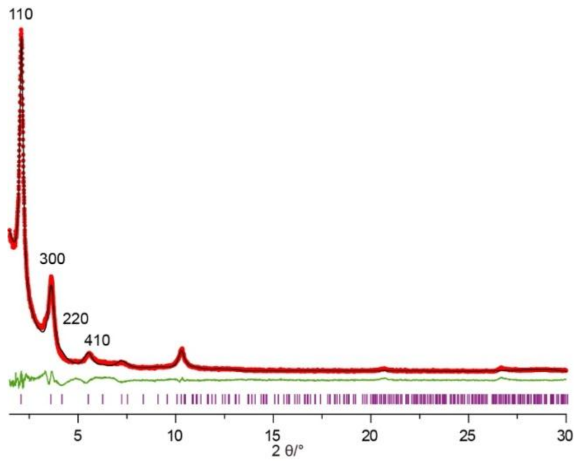

Supplementary Figure 11 X-ray diffraction patterns of Ni-IRMOF-74-V collected in Synchrotron (λ=1.23823 Å). The experimental (red), refined (black) and difference (green) patterns are displayed. The Bragg positions are marked as pink bars.

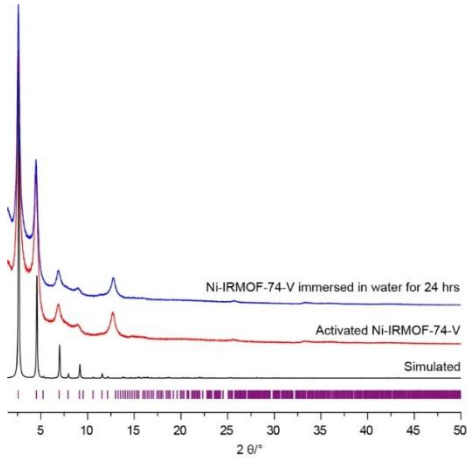

Supplementary Figure 12 Comparisons of the experimental PXRD pattern of activated Ni-IRMOF-74-V (red), after immersion in water for 24 hours (blue) with the simulated diffraction pattern (black) (Cu Kα λ = 1.5406 Å).

Supplementary Table 5 Atomic parameters of Ni-IRMOF-74-V

Atom	Occupancy	x	y	z	B_{iso}
C1	1.0	-0.178910	0.180320	0.537130	1
C2	1.0	-0.112030	0.178600	0.444140	1
C3	1.0	-0.115130	0.261850	0.550830	1
C4	1.0	-0.163380	0.159110	-0.010890	1
C5	1.0	-0.145920	0.176610	0.096880	1
C6	1.0	-0.150220	0.183900	0.275610	1
C7	1.0	-0.096410	0.217660	0.574490	1
C8	1.0	-0.113320	0.199920	0.464620	1
C9	1.0	-0.131000	0.202460	0.383040	1
C10	1.0	-0.130670	0.223050	0.406460	1
C11	1.0	-0.113880	0.240680	0.518500	1
C12	1.0	-0.096690	0.237810	0.605160	1
C13	1.0	-0.272200	0.066100	-0.728150	1
C14	1.0	-0.255930	0.082210	-0.603590	1
C15	1.0	-0.256140	0.077710	-0.403640	1
C16	1.0	-0.272780	0.056950	-0.329370	1
C17	1.0	-0.046130	0.292540	0.777370	1
C18	1.0	-0.045300	0.288040	0.981090	1
C19	1.0	-0.027280	0.303680	1.120490	1
C20	1.0	-0.306670	0.030170	-0.786290	1
C21	1.0	-0.28930	0.044940	-0.656460	1
C22	1.0	-0.289160	0.040440	-0.452000	1
C23	1.0	-0.063110	0.276100	0.658530	1
C24	1.0	-0.079260	0.255170	0.734170	1
C25	1.0	-0.078490	0.250540	0.932520	1
C26	1.0	-0.06184	0.266720	1.054020	1
C27	1.0	-0.239020	0.094970	-0.272030	1
C28	1.0	-0.222020	0.092030	-0.182570	1
C29	1.0	-0.205360	0.109620	-0.069300	1
C30	1.0	-0.205040	0.130220	-0.046910	1
C31	1.0	-0.222630	0.132770	-0.130240	1
C32	1.0	-0.239360	0.115090	-0.241900	1

Atom	Occupancy	x	y	z	U_{iso}
C33	1.0	-0.185840	0.148810	0.060460	1
C34	1.0	-0.190140	0.156100	0.239130	1
C35	1.0	-0.172680	0.173600	0.346950	1
C36	1.0	-0.220830	0.070790	-0.213020	1
C37	1.0	-0.224010	0.154040	-0.109830	1
C38	1.0	-0.157140	0.152370	-0.200920	1
Ni1	1.0	-0.006650	0.314980	1.512570	1
Ni2	1.0	-0.326980	0.017890	-1.182520	1
O1	1.0	-0.005850	0.287880	1.615190	1
O2	1.0	-0.031560	0.313150	0.686650	1
O3	1.0	-0.011170	0.322780	1.063940	1
O4	1.0	-0.028190	0.297550	1.299130	1
O5	1.0	-0.305770	0.035590	-0.966850	1
O6	1.0	-0.323260	0.011700	-0.724610	1
O7	1.0	-0.303350	0.021570	-0.374770	1
O8	1.0	-0.329120	0.044810	-1.269940	1
O9	1.0	0.333333	0.666667	0.020140	5

Supplementary Discussion:

The good agreement between the pattern of activated sample and that of the simulated model indicated the material has the same crystal structure as simulated one. The unaltered PXRD pattern of the activated sample after immersion in water for 24 hours demonstrates that Ni-IRMOF-74-II is stable in water (Supplementary Figure 6). Elemental analysis (activated sample): Found (%): C, 30.71; H, 4.18.

The good agreement between the pattern of activated sample and that of the simulated model indicated the material has the same crystal structure as simulated one. The unaltered PXRD pattern of the activated sample after immersion in water for 24 hours demonstrates that Ni-IRMOF-74-III is stable in water (Supplementary Figure 8). Elemental analysis (activated sample): Found (%): 44.77; H, 4.85.

The good agreement between the pattern of activated sample and that of the simulated model indicated the material has the same crystal structure as simulated one. The unaltered PXRD pattern of the activated sample after immersion in water for 24 hours demonstrates that Ni-IRMOF-74-IV is stable in water (Supplementary Figure 10). Elemental analysis (activated sample): Found (%): C, 43.91; H, 2.86.

The good agreement between the pattern of the activated sample and that of the simulated model indicated the material has the same crystal structure as simulated one. The unaltered PXRD pattern of the activated sample after immersion in water for 24 hours demonstrates that Ni-IRMOF-74-V is stable in water (Supplementary Figure 12). Elemental analysis (activated sample): Found (%): C, 44.02; H, 2.74.

Synthesis of MIL-101 (Cr):

MIL-101(Cr) was synthesized and activated according to the literature procedure[6]. The good agreement between the pattern of the activated sample and that of the simulated model indicated the material has the same crystal structure as simulated one (Supplementary Figure 13).

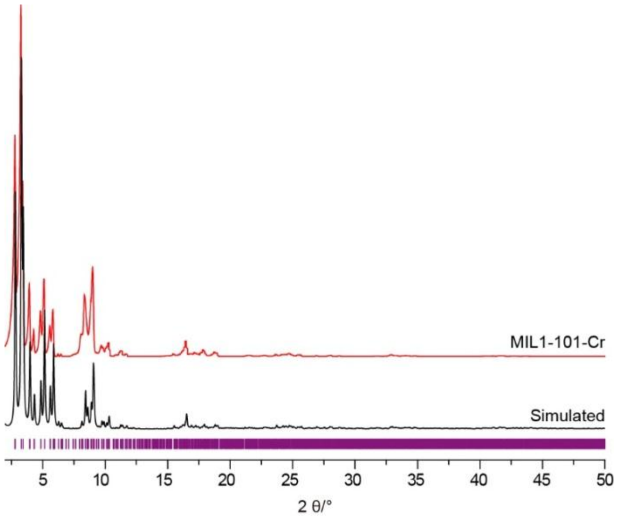

Supplementary Figure 13 Comparison of the experimental PXRD pattern of the activated MIL-101 (Cr) (red) with the simulated diffraction pattern (black) (Cu Kα λ = 1.5406 Å).

Synthesis of UiO-66 and UiO-67:

UiO-66 and UiO-67 was synthesized and activated according to the literature procedure[7].The good agreement between the pattern of the activated sample and that of the simulated model indicated the material has the same crystal structure as simulated one (Supplementary Figures 14 and 15).

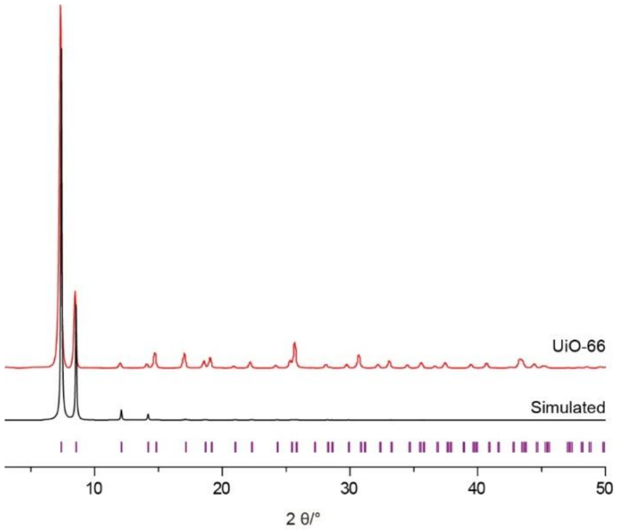

Supplementary Figure14 Comparison of the experimental PXRD pattern of the activated UiO-66 (red) with the simulated diffraction pattern (black) (Cu Kα λ = 1.5406 Å).

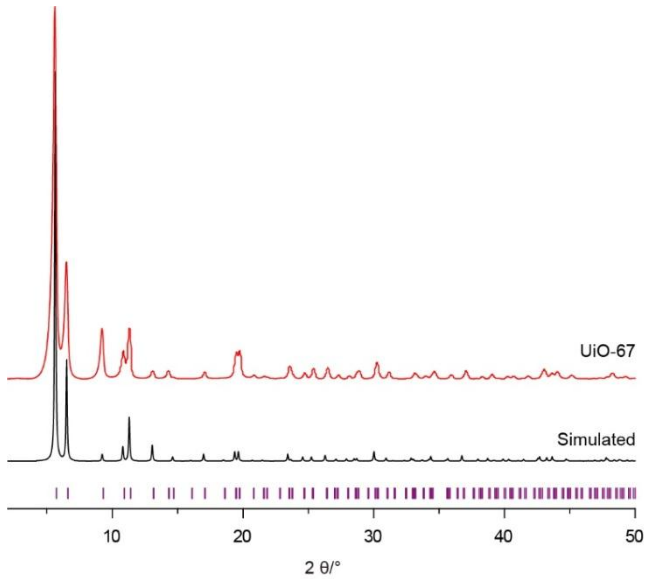

Supplementary Figure 15 Comparison of the experimental PXRD pattern of the activated UiO-67 (red) with the simulated diffraction pattern (black) (Cu Kα λ = 1.5406 Å).

Synthesis of SBA-15:

SBA-15 was synthesized as the literature reported[8] (Supplementary Figure 16).

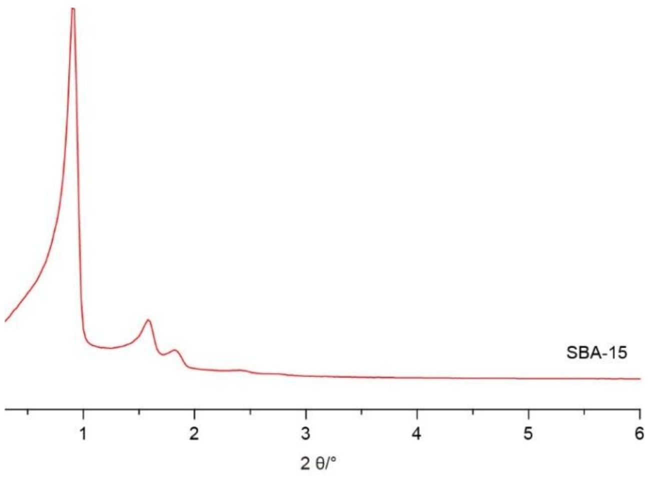

Supplementary Figure 16 Experimental PXRD pattern of the activated SBA-15 (Cu Kα λ = 1.5406 Å).

Synthesis of nanocrystals of Ni-IRMOF-74-II:

Nanocrystals Ni-IRMOF-74-II was synthesized using the similar method for the synthesis of nanocrystals of Ni-MOF-74[9]. $Ni(NO_3)_2{\cdot}6H_2O$ (79 mg, 0.27 mmol) and linker II (28 mg, 0.10 mmol) were dissolved with 6.75 mL anhydrous DMF in a 20 mL vial. The vial was stirred vigorously till it becomes clear solution. To this solution, a mixture of 0.45 mL of ethanol, 0.45 mL of deionized water and 0.05 mL triethylamine

was added quickly. The vial was then vigorously stirred for 2 h. Then, the solid was separated by centrifugation and dispersed in anhydrous DMF again. This solution was heated in an isothermal oven at 100 °C for 2 h to dissolve impurities trapped in the MOF pores. Then, the solution was cooled down to room temperature and exchanged by anhydrous DMF three times. After washing with anhydrous DMF, the solid was centrifuged and exchanged by EtOH for six times to remove the DMF residues. Finally, double-distilled water was used to wash the solid for nine times in order to remove ethanol from Ni-IRMOF-74-II. The unchanged peak positions between the pattern of nanocrystals and that of the bulk material proved they are the same crystal structure. Broadening in the PXRD peaks of Ni-IRMOF-74-II nanocrystals indicated a much smaller particle size according to *Debye-Scherrer* Equation (Supplementary Figure 17).

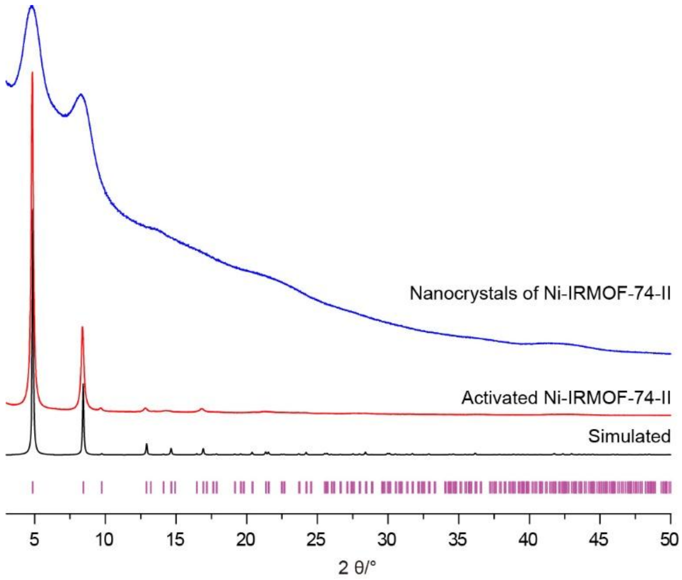

Supplementary Figure 17 Comparison of the experimental PXRD pattern of nanocrystals of Ni-IRMOF-74-II (blue), the activated Ni-IRMOF-74-II (red), the simulated Ni-IRMOF-74-II diffraction pattern (black) (Cu Kα λ = 1.5406 Å).

Synthesis of nanocrystals of Ni-IRMOF-74-III:

$Ni(NO_3)_2·6H_2O$ (79 mg, 0.27 mmol), and linker III (38 mg, 0.10 mmol) were dissolved with 6.75 mL anhydrous DMF in a 20 mL vial and followed the method above. The unchanged peak positions between the pattern of nanocrystals and that of the bulk material proved they are the same crystal structure. Broadening in the PXRD peaks of Ni-IRMOF-74-III nanocrystals indicates a much smaller particle size according to *Debye-Scherrer* Equation (Supplementary Figure 18).

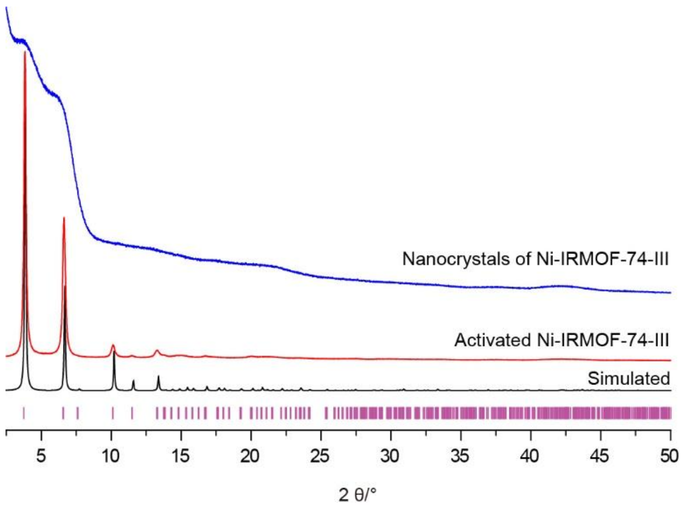

Supplementary Figure 18 Comparison of the experimental PXRD pattern of nanocrystals of Ni-IRMOF-74-III (blue), the activated Ni-IRMOF-74-III (red), the simulated Ni-IRMOF-74-III diffraction pattern (black) (Cu Kα λ = 1.5406 Å).

Elemental analysis

Inductively Coupled Plasma-Atomic Emission Spectrometry (ICP-AES) technology was performed on a THERMO Intrepid XSP Radial ICP-AES instrument. Aqueous solution of nanocrystals was well dispersed in a tube by sonicating for 15 min and 1000 μL solution was taken and dried under 85 °C for 48 hours to remove water. Then 1 mL aqua regia was added to dissolve the MOF to release the Ni^{2+} and the solution was diluted to 10 mL in a volumetric flask using deionized water. The ratio of Ni was quantified by ICP-AES and pure MOF formula was used to calculate the real mass concentration of MOF (Supplementary Table 6).

Supplementary Table 6 Composition of Ni-IRMOF-74-II nanocrystals.

Formula of Ni-IRMOF-74-II	Ni^{2+} concentration by ICP-AES	Ni^{2+} mass/mg	Aqueous dispersion/μL	Concentration of Ni-IRMOF-74-II (mg/mL)
$C_7H_4NiO_4$	59.6	596	1000	2.16

Particle size distribution

Particle size was measured by laser doppler micro-electrophoresis (DLS). Here a Zetasizer Nano-ZS, from Malvern UK was used. The particle sizes of nanocrystals were around 100 -150 nm (Supplementary Figure 19 and 20) with a low PDI values (0.151 and 0.170), indicating uniform sizes for nano-sized Ni-IRMOF-74-II & III (Supplementary Table 7).

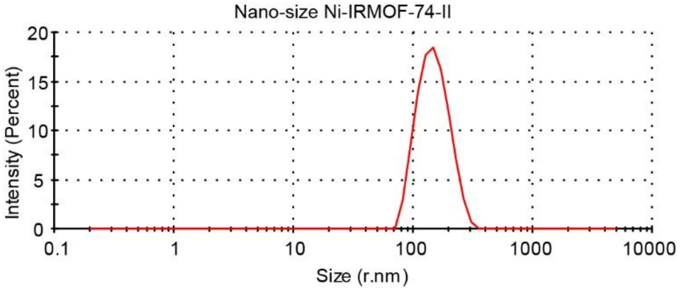

Supplementary Figure 19 DLS size distribution of Ni-IRMOF-74-II nanocrystals in aqueous dispersion

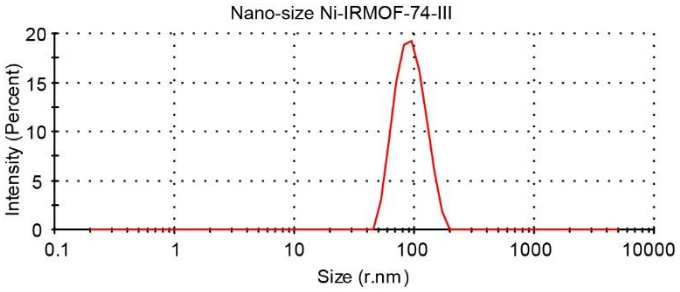

Supplementary Figure 20 DLS size distribution of Ni-IRMOF-74-III nanocrystals in aqueous dispersion.

Supplementary Table 7 Particle size distribution analysis.

Nanocrystals	Hydrodynamic radius (nm)	PDI
Ni-IRMOF-74-II	146.4 ± 56.85	0.151
Ni-IRMOF-74-III	89.91 ± 37.10	0.170

N_2 adsorption analysis

All N_2 adsorptions experiments were measured on a Quantachrome Autosorb-1 automatic volumetric instrument. A liquid nitrogen bath (77 K) was used for isotherm measurements. Ultra-high purity grade N_2 was used to the adsorption experiments. The BET analysis is performed by plotting $x/v(1 - x)$ vs x, where $x = P/P_0$ (P_0 = 1 bar) and v is the volume of nitrogen adsorbed per gram of MOF at STP (Supplementary Figure 21-30). This analysis produces a curve typically consisting of three regions: concave to the x axis at low pressures, linear at intermediate pressures, and convex to the x axis at high pressures. The slope ($[c - 1]/v_m c$) and y intercept ($1/v_m c$) of this linear region give the monolayer capacity, v_m, that is then used to calculate the surface area from $A = v_m \sigma_0 N_{AV}$, where σ_0 is the cross-section

al area of the adsorbate at liquid density (16.2 $Å^2$ for nitrogen) and N_{AV} is Avogadro's number[10].

Pore size distributions for MOFs were analyzed using quenched solid state density functional theory (QSDFT) based on a carbon model containing cylindrical pores. The analysis of all Ni-IRMOF-74 fit well with the simulated structures. The pore size gradually increases as the length of organic strut from Ni-IRMOF-74-II to Ni-IRMOF-74-V (Supplementary Table 8) exhibiting a type IV isotherm. Estimated distributions for these MOFs are very close to pore diameters (the short dimension of the pore aperture metrics) calculated from simulated crystal structures. With the inclusion of ssDNA, the surface area and pore volume of Ni-IRMOF-74-II and Ni-IRMOF-74-III decrease dramatically indicating a highly efficient absorption of ssDNA into their pores (Supplementary Table 8).

Supplementary Table 8 Porosity parameters from nitrogen absorption analysis.

Compound	Framework molecular formula	BET SA (m^2/g)	Pore volume (cm^3/g)	Pore width (nm)	Reference
Ni-IRMOF-74-II	$C_7H_4NiO_4$	1930	0.77	1.8	This work
Mg-IRMOF-74-II	$C_7H_4MgO_4$	2510	1.04	1.7	Ref 1
Ni-IRMOF-74-III	$C_{10}H_6NiO_4$	2120	0.93	2.4	This work
Mg-IRMOF-74-III	$C_{10}H_6MgO_4$	2440	1.23	2.2	Ref 1
Ni-IRMOF-74-IV	$C_{15}H_{13}NiO_4$	1920	1.14	3.0	This work
Mg-IRMOF-74-IV	$C_{15}H_{13}MgO_4$	2480	1.6	2.8	Ref 1
Ni-IRMOF-74-V	$C_{19}H_{16}NiO_4$	1900	1.39	3.6	This work
Mg-IRMOF-74-V	$C_{19}H_{16}MgO_4$	2230	1.89	3.5	Ref 1

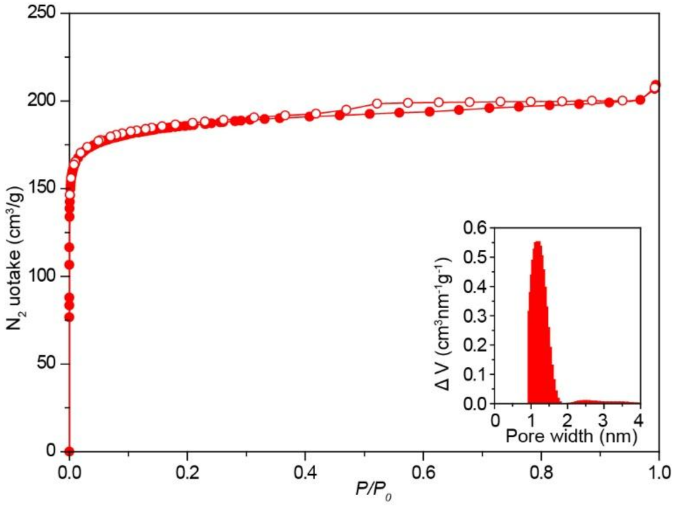

Supplementary Figure 21 Nitrogen isotherm of Ni-MOF-74 at 77 K**.** Filled and open symbols represent absorption and desorption branches, respectively. Points are connected to provide clear shape of the isotherm. Inserted plot shows the pore size distribution of Ni-MOF-74.

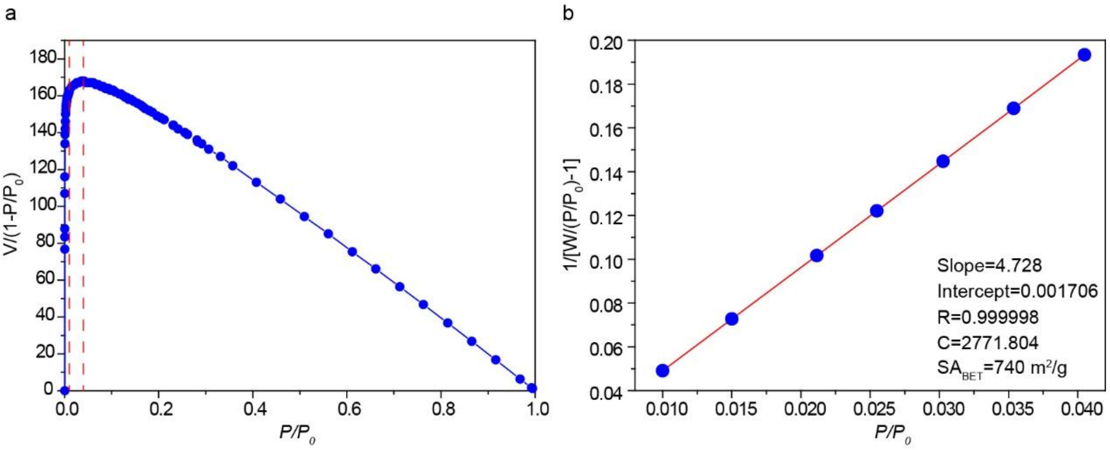

Supplementary Figure 22 BET area calculation for Ni-MOF-74 from simulated nitrogen isotherm at 77 K (a) Only points between the dashed line are selected based on the first consistency criterion, (b) Plot to select linear P/P_0 range.

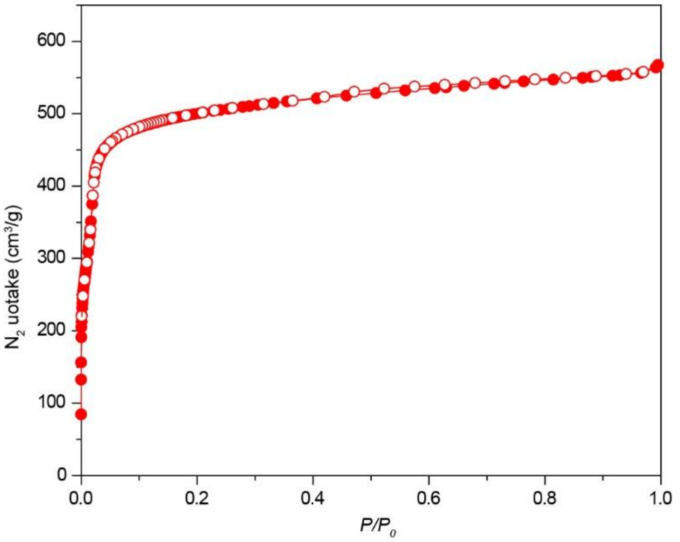

Supplementary Figure 23 Nitrogen isotherm of Ni-IRMOF-74-II at 77 K**.** Filled and open symbols represent absorption and desorption branches, respectively. Points are connected to provide clear shape of the isotherm. Inserted plot shows the pore size distribution of Ni-IRMOF-74-II.

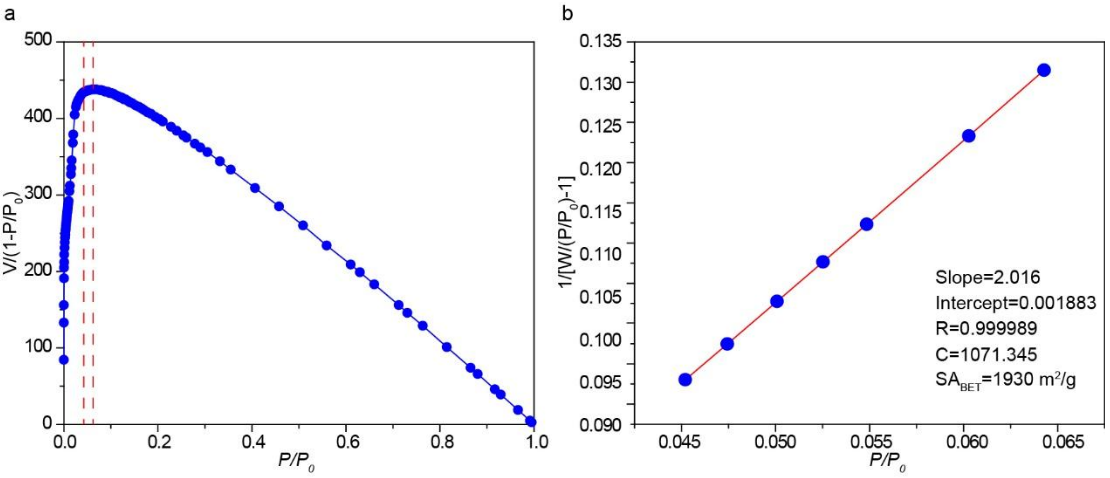

Supplementary Figure 24 BET area calculation for Ni-IRMOF-74-II from simulated nitrogen isotherm at 77 K (a) Only points between the dashed lines are selected based on the first consistency criterion, (b) Plot to select linear P/P_0 range.

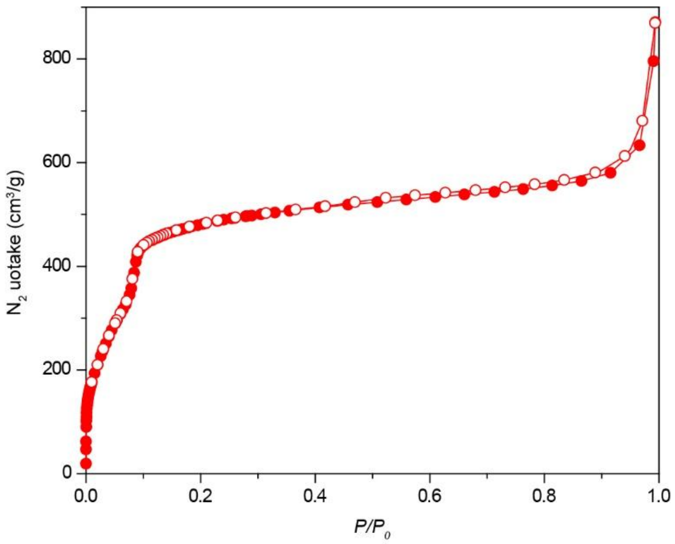

Supplementary Figure 25 Nitrogen isotherm of Ni-IRMOF-74-III at 77 K**.** Filled and open symbols represent absorption and desorption branches, respectively. Points are connected to provide clear shape of the isotherm. Inserted plot shows the pore size distribution of Ni-IRMOF-74-III.

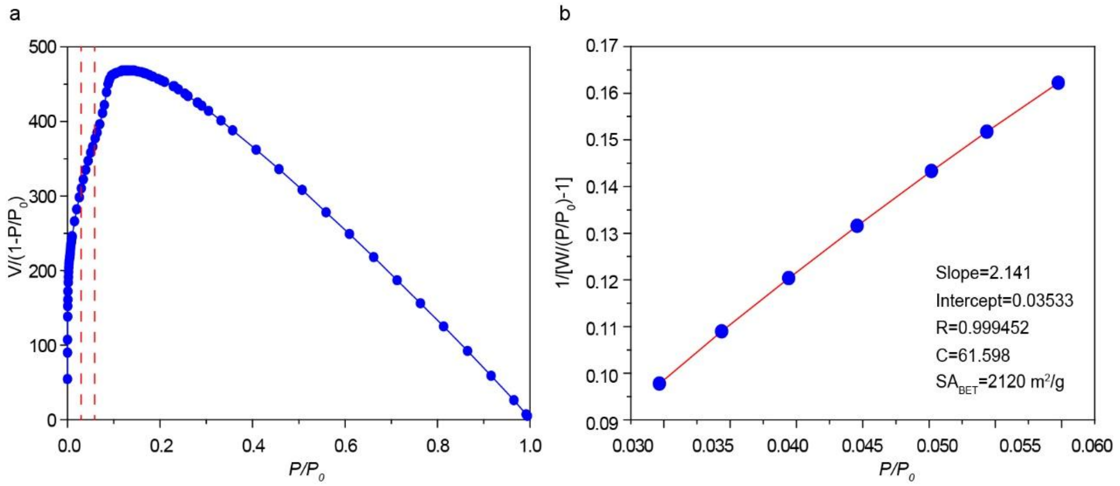

Supplementary Figure 26 BET area calculation for pristine Ni-IRMOF-74-III from simulated nitrogen isotherm at 77 K (a) Only points between the dashed lines are selected based on the first consistency criterion, (b) Plot to select linear P/P_0 range.

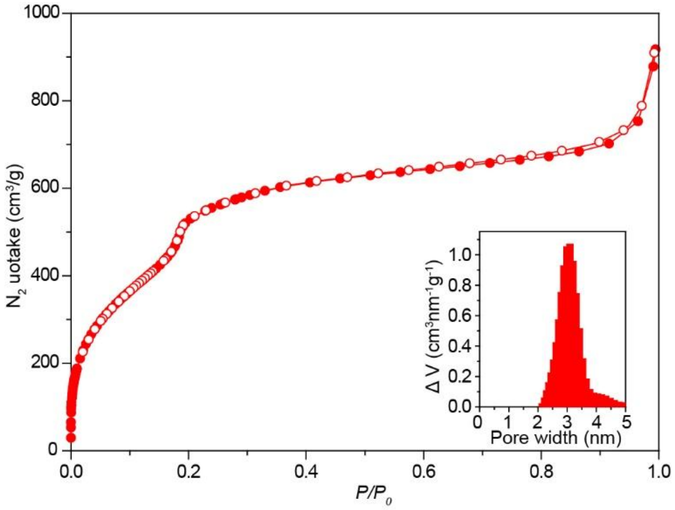

Supplementary Figure 27 Nitrogen isotherm of Ni-IRMOF-74-IV at 77 K**.** Filled and open symbols represent absorption and desorption branches, respectively. Points are connected to provide clear shape of the isotherm. Inserted plot shows the pore size distribution of Ni-IRMOF-74-IV.

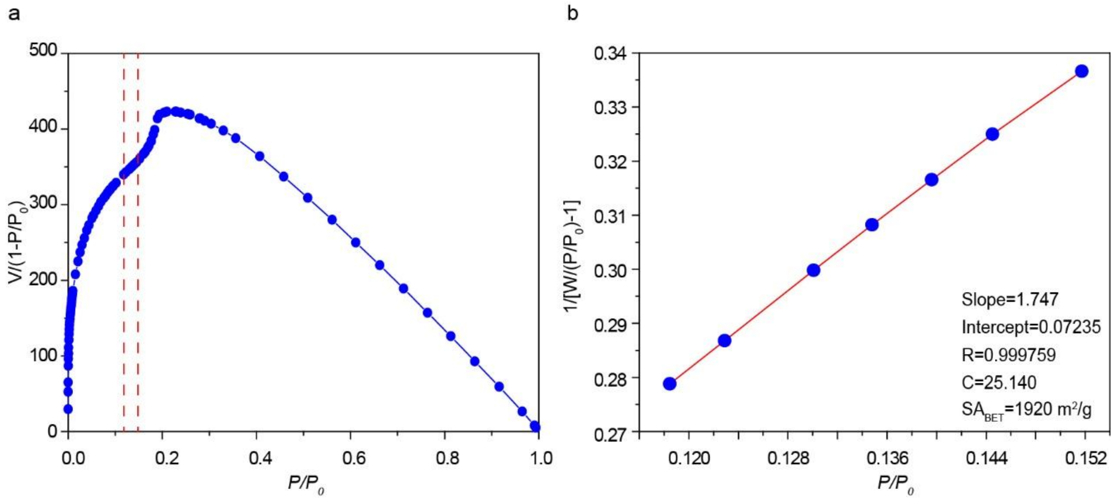

Supplementary Figure 28 BET area calculation for Ni-IRMOF-74-IV from simulated nitrogen isotherm at 77 K (a) Only points between the dashed lines are selected based on the first consistency criterion, (b) Plot to select linear P/P_0 range.

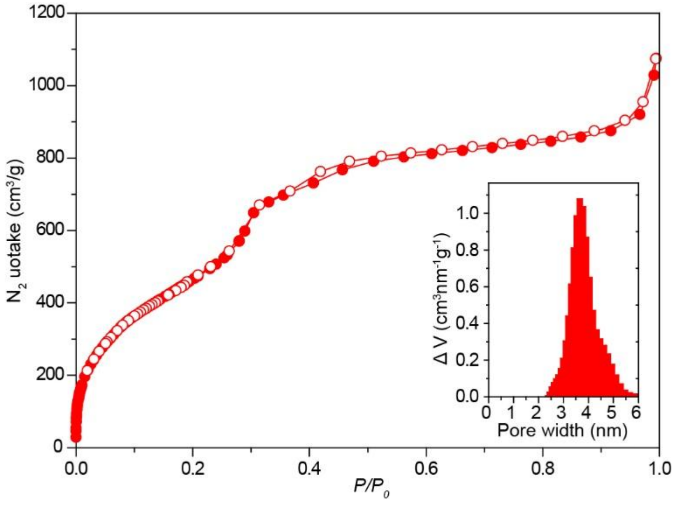

Supplementary Figure 29 Nitrogen isotherm of Ni-IRMOF-74-V at 77 K**.** Filled and open symbols represent absorption and desorption branches, respectively. Points are connected to provide clear shape of the isotherm. Inserted plot shows the pore size distribution of Ni-IRMOF-74-V.

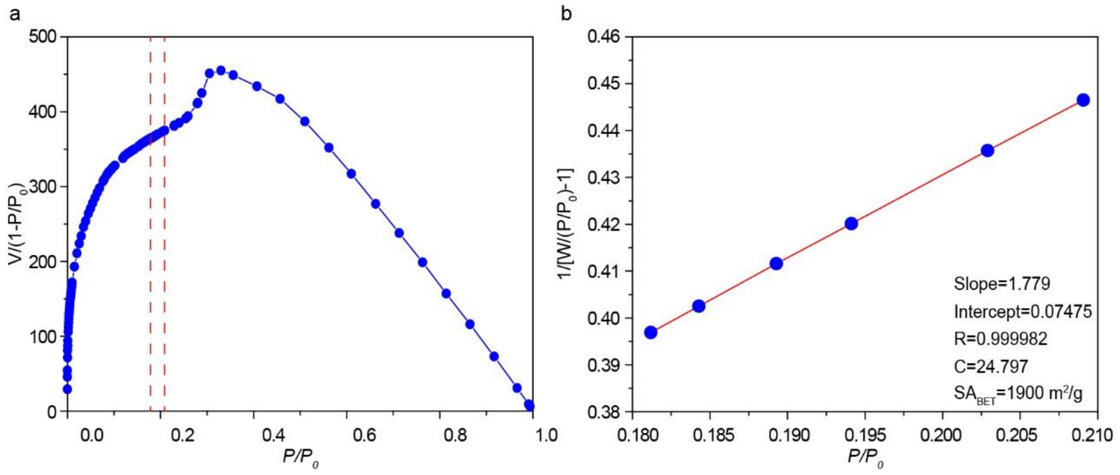

Supplementary Figure 30 BET area calculation for Ni-IRMOF-74-V from simulated nitrogen isotherm at 77 K (a) Only points between the dashed lines are selected based on the first consistency criterion, (b) Plot to select linear P/P_0 range.

Thermogravimetric analysis (TG)

All samples were performed on a TGA Q500 thermal gravimetric analyzer with samples held in aluminum oxide pans in a continuous airflow atmosphere (Balance gas: N_2: 20.0 mL/min, Sample gas: Air: 20.0 mL/min). The samples were heated at a constant rate of 10 °C during all TGA experiments.

In each TGA plot, the slight weight loss before 100 °C was attributed to the evaporation of water introduced into MOF from air during the sample mounting. The major weight loss evidenced (around 300 °C) was caused by the destruction of Ni-

IRMOF series (Supplementary Figure 31-36). The major weight loss of MIL-101-Cr was around 350 °C (Supplementary Figure 37), while UiO-66 and UiO-67 decomposed at around 550 °C (Supplementary Figure 38 and 39).

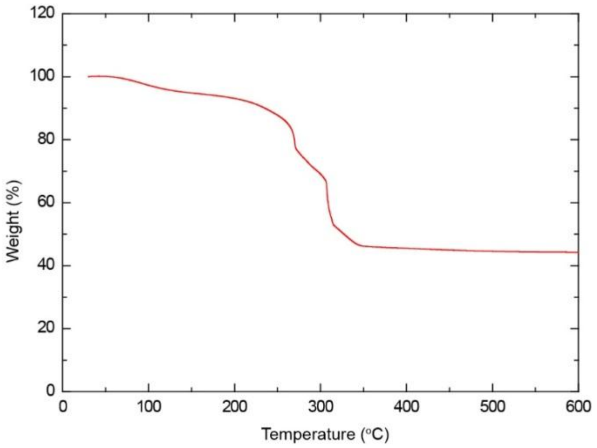

Supplementary Figure 31 TGA trace of activated Ni-MOF-74.

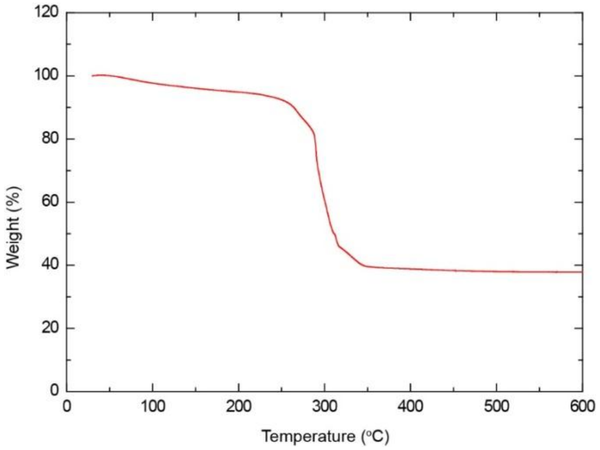

Supplementary Figure 32 TGA trace of activated Ni-IRMOF-74-II.

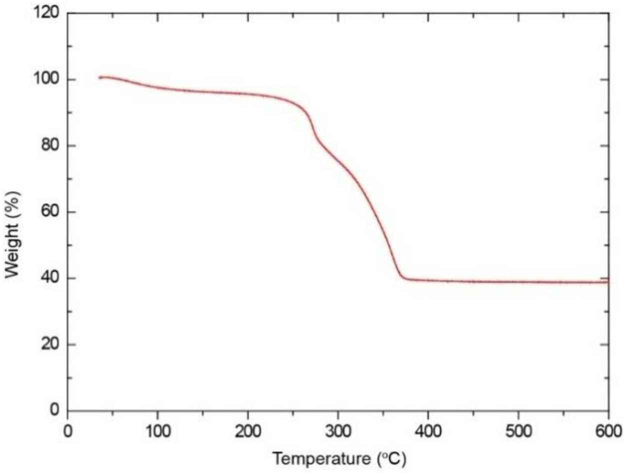

Supplementary Figure 33 TGA trace of activated Ni-IRMOF-74-III.

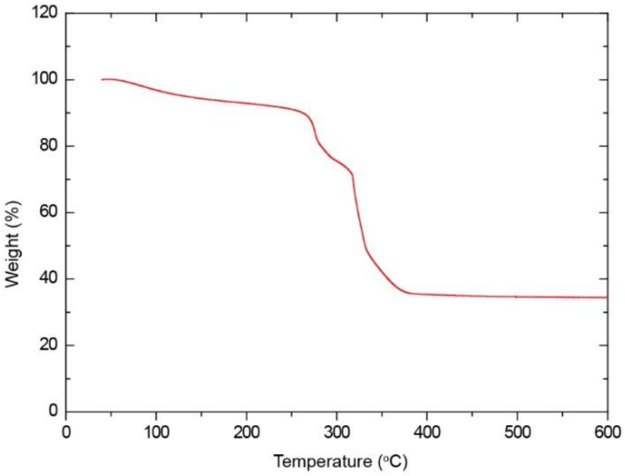

Supplementary Figure 34 TGA trace of activated Ni-IRMOF-74-IV.

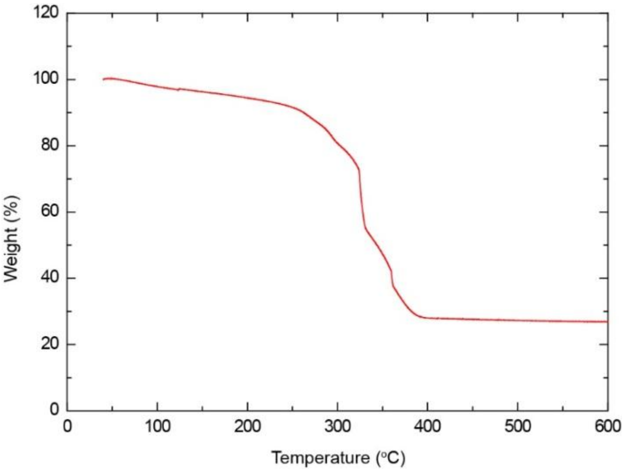

Supplementary Figure 35 TGA trace of activated Ni-IRMOF-74-V.

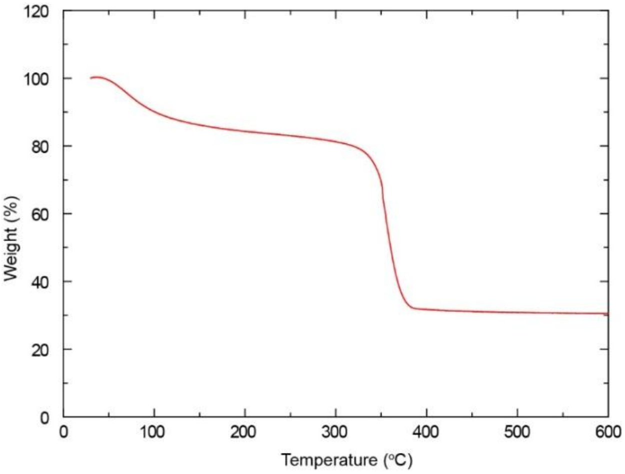

Supplementary Figure 36 TGA trace of nanocrystals Ni-IRMOF-74-II.

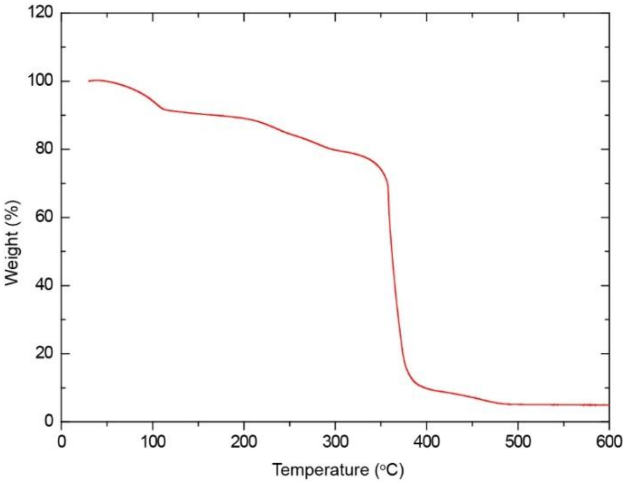

Supplementary Figure 37 TGA trace of MIL-101-Cr.

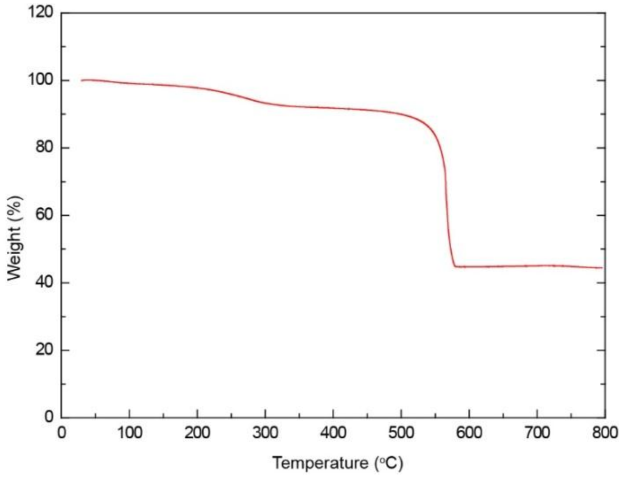

Supplementary Figure 38 TGA trace of UiO-66.

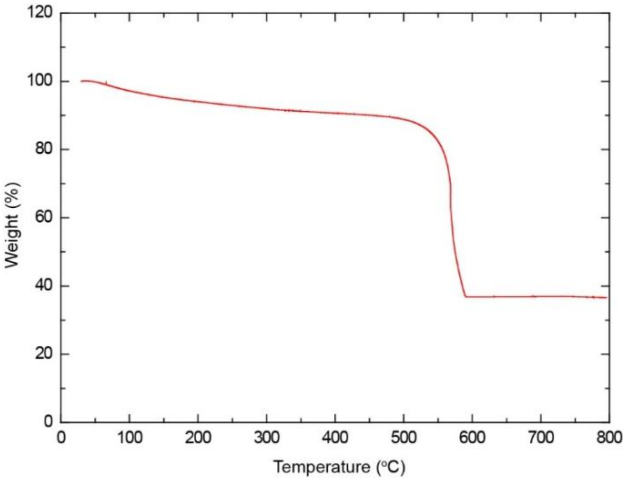

Supplementary Figure 39 TGA trace of UiO-67.

Scanning electron microscopy (SEM) imaging of MOFs

MIL-101-Cr was dispersed on the surface of sticky carbon attached on a copper sample holder and then coated with platinum at a pressure of 8.00 Pa and ambient temperature in an argon atmosphere for 100 s using a current of 15 mA. SEM measurements of MIL-101-Cr were performed on a Merlin Compact FE-SEM, Zeiss with accelerating voltage of 5.0 kV. Other samples of synthesized Ni-IRMOF-74, UiO-66 and UiO-67 were dispersed on the surface of sticky carbon attached on a copper sample holder and then under evacuation for 15 minutes without coating. The SEM measurements of other samples were performed on a Hitachi S4800, with accelerating voltage of 1.0 kV. Nanocrystals of Ni-IRMOF-74-II & III were slightly grinded and performed on a FEI Varios, with accelerating voltage of 0.5 kV.

Multiple samples of each Ni-IRMOF-74 were surveyed to confirm the homogeneity. All of the Ni-IRMOF-74-II to V samples exhibit needle shape with uniform sizes and the needles form a spherical morphology (Supplementary Figure 41-44). MIL-101-Cr, UiO-66 and UiO-67 were the same morphology as previously reported[51, 52]. The nanocrystals of Ni-IRMOF-74-II and -III exhibit a uniform particle size around 100 nm and similar to the morphology of Ni-MOF-74 nanocrystals as reported[54] (Supplementary Figure 48 and 49).

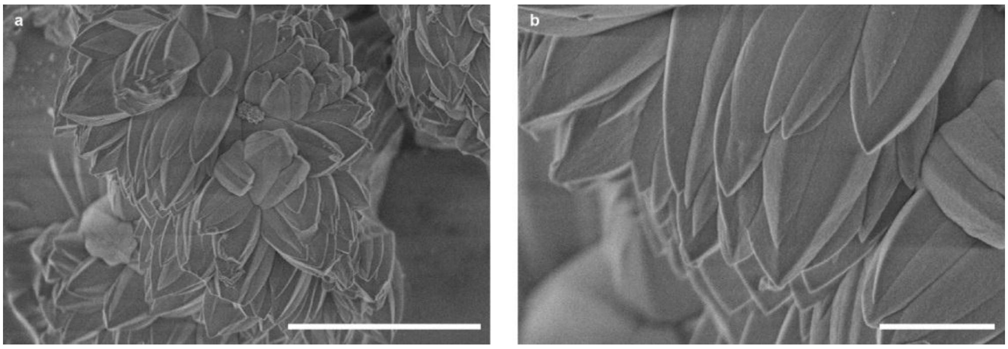

Supplementary Figure 40 SEM images of Ni-MOF-74, scale bar, 5 μm (a) and 1 μm (b).

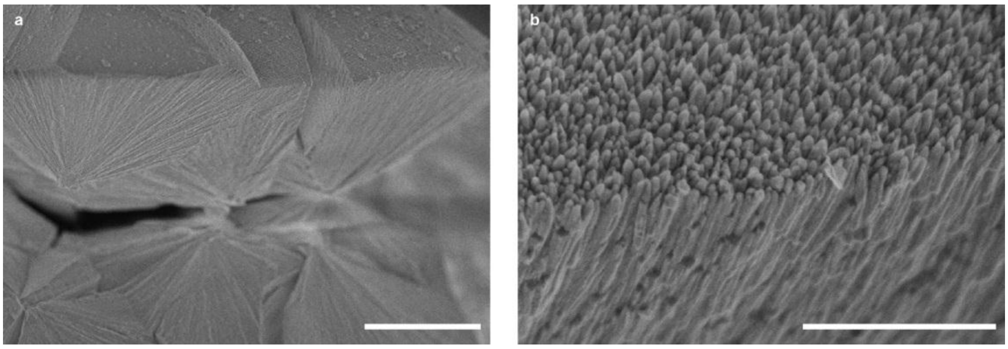

Supplementary Figure 41 SEM images of Ni-IRMOF-74-II, scale bar, 10 μm (a) and 1 μm (b).

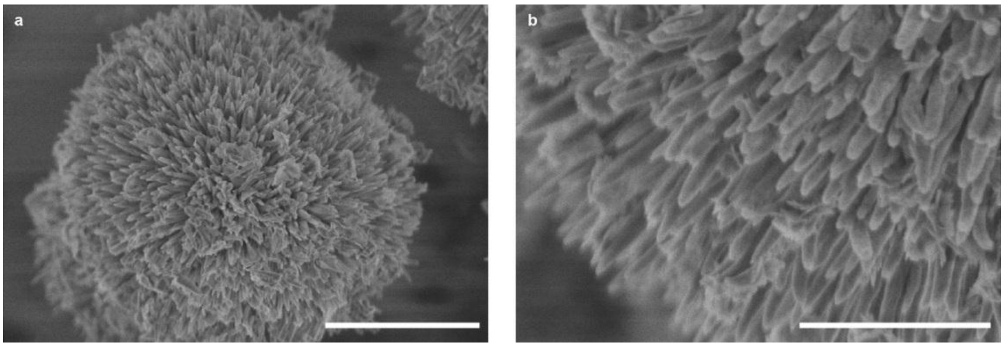

Supplementary Figure 42 SEM images of Ni-IRMOF-74-III, scale bar, 2 μm (a) and 1 μm (b).

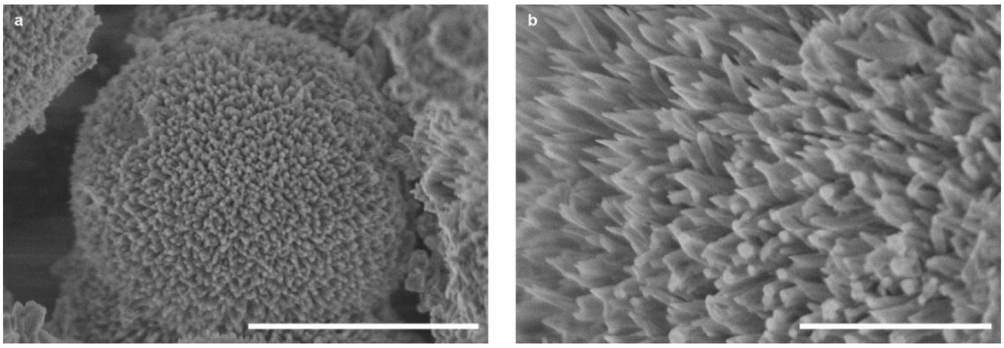

Supplementary Figure 43 SEM images of Ni-IRMOF-74-IV, scale bar, 2 μm (a) and 1 μm (b).

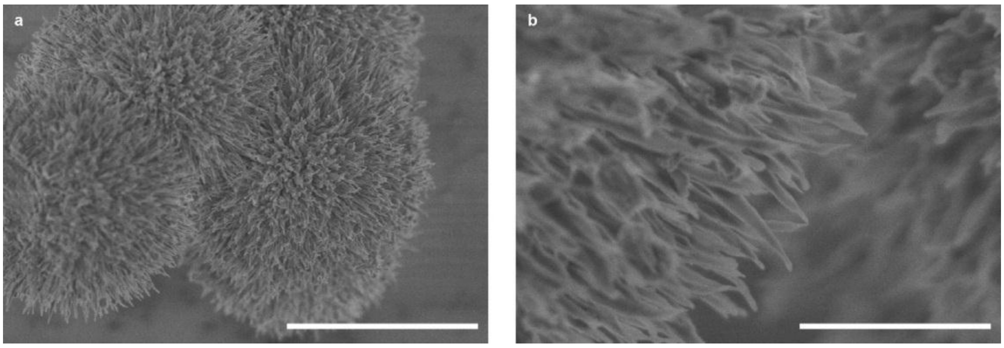

Supplementary Figure 44 SEM images of Ni-IRMOF-74-V, scale bar, 5 μm (a) and 1 μm (b).

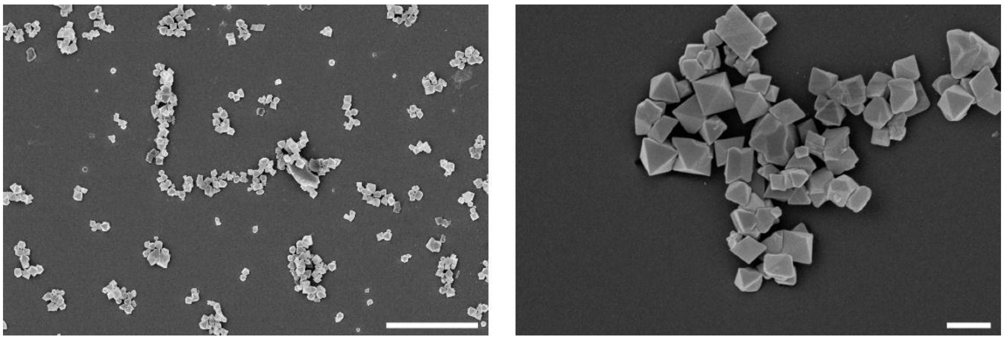

Supplementary Figure 45 SEM images of MIL-101, scale bar, 2 μm (a) and 200 nm (b).

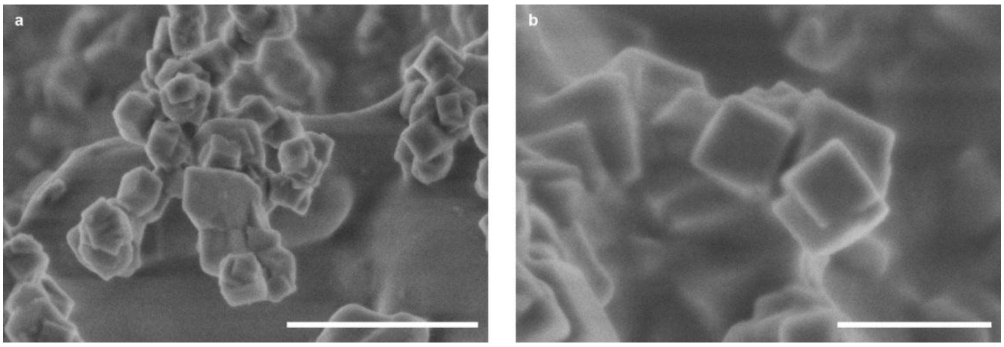

Supplementary Figure 46 SEM images of UiO-66, scale bar, 500 nm (a) and 200 nm (b).

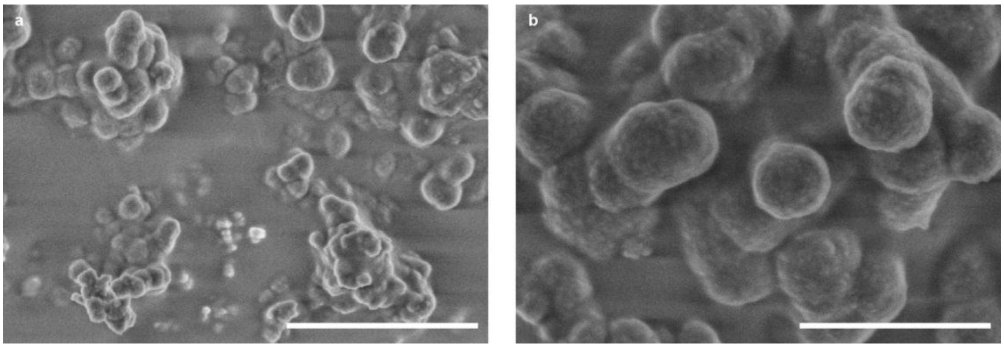

Supplementary Figure 47 SEM images of UiO-67, scale bar, 1 μm (a) and 500 nm (b).

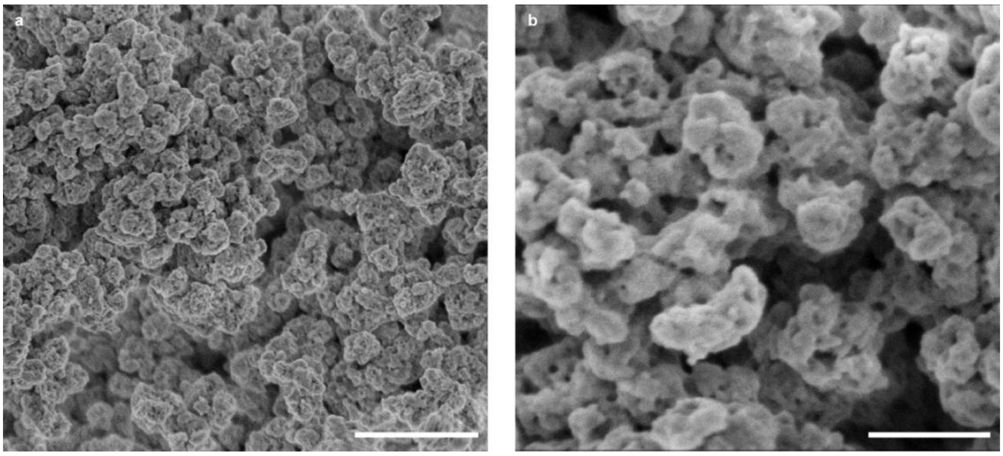

Supplementary Figure 48 SEM images of Ni-IRMOF-74-II nanocrystals, scale bar, 300 nm (a) and 100 nm (b).

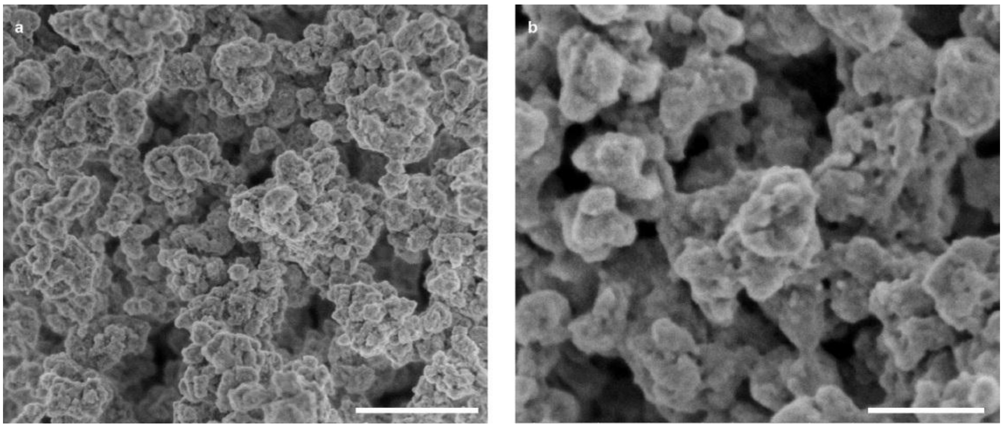

Supplementary Figure 49 SEM images of Ni-IRMOF-74-III nanocrystals, scale bar, 300 nm (a) and 100 nm (b).

Infrared (IR) spectra of MOFs

METHODS:

All IR spectra were recorded on a Thermo Nicolet iS10 IR spectroscopy made by Thermofisher and samples were tableted and KBr was utilized as background. The wavelength range is 4000 cm^{-1} to 400 cm^{-1}. The absence of strong and broad peaks of O-H around 3250 cm^{-1} indicates that the hydroxyl groups and carboxylic groups were fully coordinated to metal sites (Supplementary Figure 50-54). The absence of broad peaks at around 3000 cm^{-1} to 2500 cm^{-1} indicated the carboxylic groups were fully coordinated metal sites (Supplementary Figure 55-57).

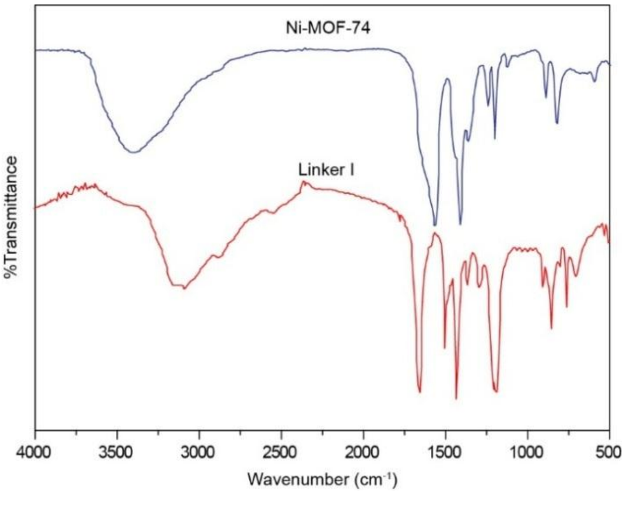

Supplementary Figure 50 IR spectra of Ni-MOF-74 and linker I.

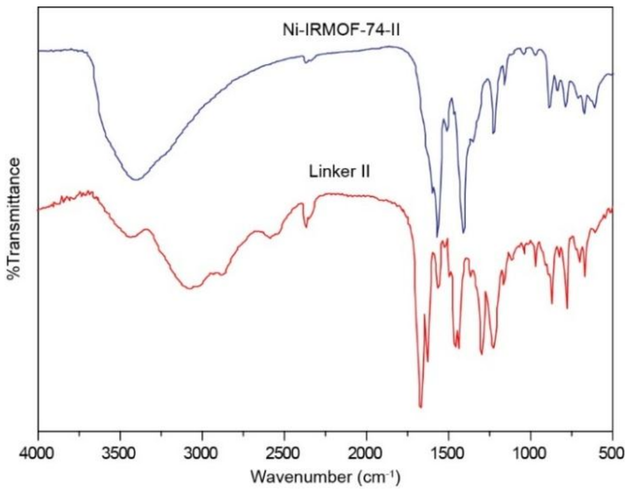

Supplementary Figure 51 IR spectra of Ni-IRMOF-74-II and linker II.

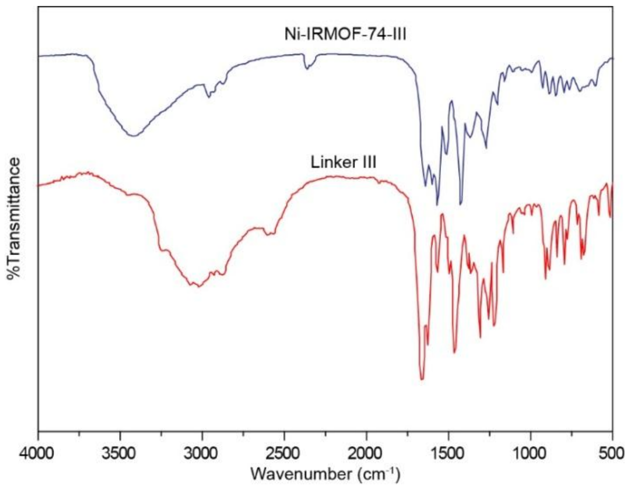

Supplementary Figure 52 IR spectra of Ni-IRMOF-74-III and linker III.

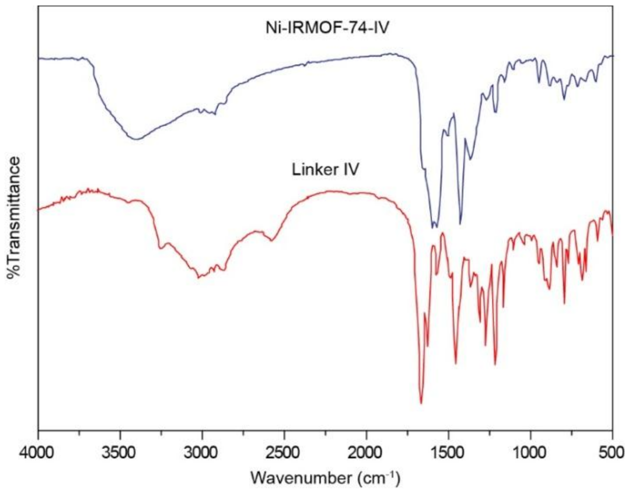

Supplementary Figure 53 IR spectra of Ni-IRMOF-74-IV and linker IV.

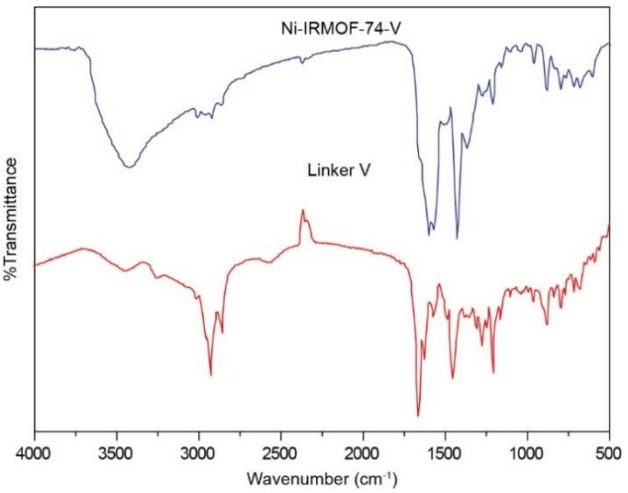

Supplementary Figure 54 IR spectra of Ni-IRMOF-74-V and linker V.

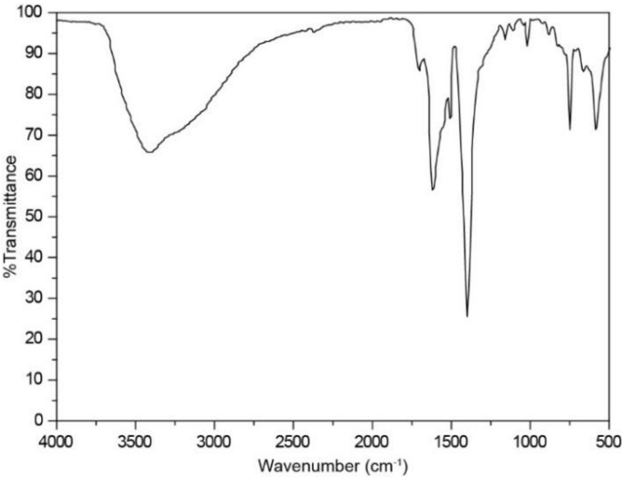

Supplementary Figure 55 IR spectra of MIL-101-Cr.

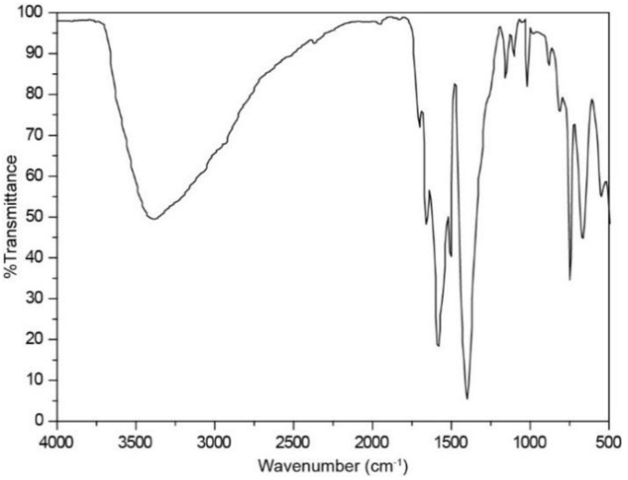

Supplementary Figure 56 IR spectra of UiO-66.

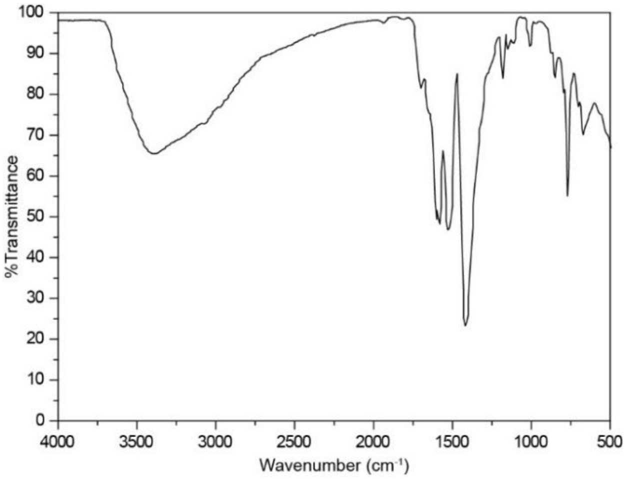

Supplementary Figure 57 IR spectra of UiO-67.

Supplementary Method 3.

Characterization of ssDNA Loaded MOFs

Control experiments of fluorophore molecules uptake in MOFs

In this experiment, we use two fluorescent molecules, 6-carboxyfluorescein (FAM) and 6-carboxytetramethylrhodamine (TAMRA), as separate controls to see if they interact with the MOFs. TAMRA is a dye with conjugate structure while FAM is easier to ionize and carry negative charge due to the deprotonation of carboxylic and phenol groups. They are good indicators for the existence of π-π interactions and electrostatic force, respectively. Fluorescent dye FAM and TAMRA (50 nM) interacted with 62 μM of vectors (Ni-IRMOF-74, -II, -III, -IV, V, UiO-66, UiO-67, MIL-101-Cr, respectively) under 1000 rpm vibration at 37 °C for 3 h. The fluorescence of the solution was then measured and the results were shown in Supplementary Figure 58. The adsorption amount of TAMRA increased from IRMOF-74-II to -V as the number of phenylene units added up, which indicated an increase in π-π interaction, while MIL-101 exhibited negligible uptake due to the limited number of phenyl units in their constituent linkers. In the case of FAM, IRMOF-74-II to -V didn't adsorb any FAM molecule due to the same negative charge of the framework (Supplementary Table 14). In contrast, significant uptake of FAM was observed in MIL-101 due to the positive charge of the framework and thereby strong electrostatic interaction with FAM. Although UiO-66 and UiO-67 are also positively charge (Supplementary Figure 88), their limited pore sizes prevent the entering of both FAM and TAMRA molecules.

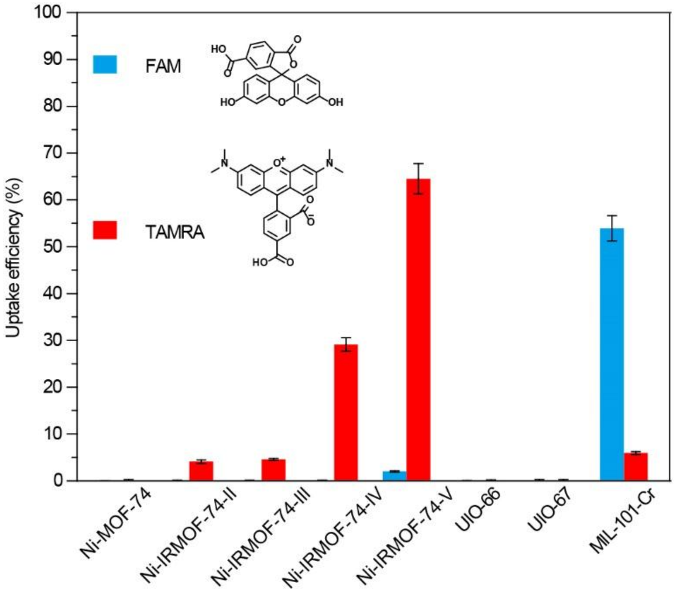

Supplementary Figure 58 Uptake of fluorophores in various MOF vectors (n = 3 technical replicates; bars represent mean ± s.d.).

Inclusion of entire ssDNA into MOF pores

5' end of ssDNA was labeled with FAM and the 3' end with TAMRA. ssDNA (50 nM) and Ni-IRMOF-74- II (24 μg/ mL) were dispersed under 1000 rpm vibration at 37 °C before measurement of fluorescence intensity at different time. Excitation and emission for FAM are 480 nm and 520 nm, respectively. Excitation and emission for TAMRA are 550 nm and 580 nm, respectively. Concurrent decay of fluorescence at both wavelengths was observed in the solution (Supplementary Figure 59), indicating the proper accommodation of entire ssDNA into MOF pores, rather than partial inclusion.

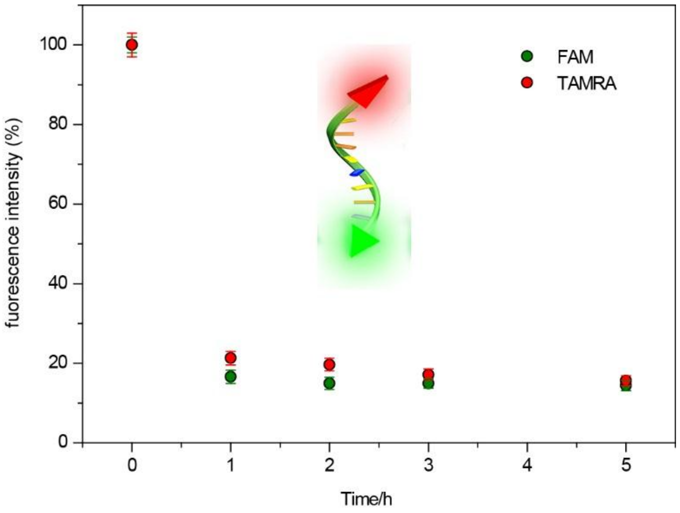

Supplementary Figure 59 Fluorescence quench of doubly labeled ssDNA using Ni-IRMOF-74-II as vector (n = 3 technical replicates; bars represent mean ± s.d.).

Stability of ssDNA loaded Ni-IRMOF-74-III

The activated sample of Ni-IRMOF-74-III was immersed in aqueous solution of pH = 3, pH = 11, with ssDNA, cDNA and cell culture (DMEM), respectively. The samples were collected after immersion for 24 hours followed by washing with deionized water and ethanol for three times, respectively. The unaltered PXRD pattern of the activated sample after immersion in various solutions for 24 hours demonstrates that Ni-IRMOF-74-III is stable in various aqueous media (Supplementary Figure 60).

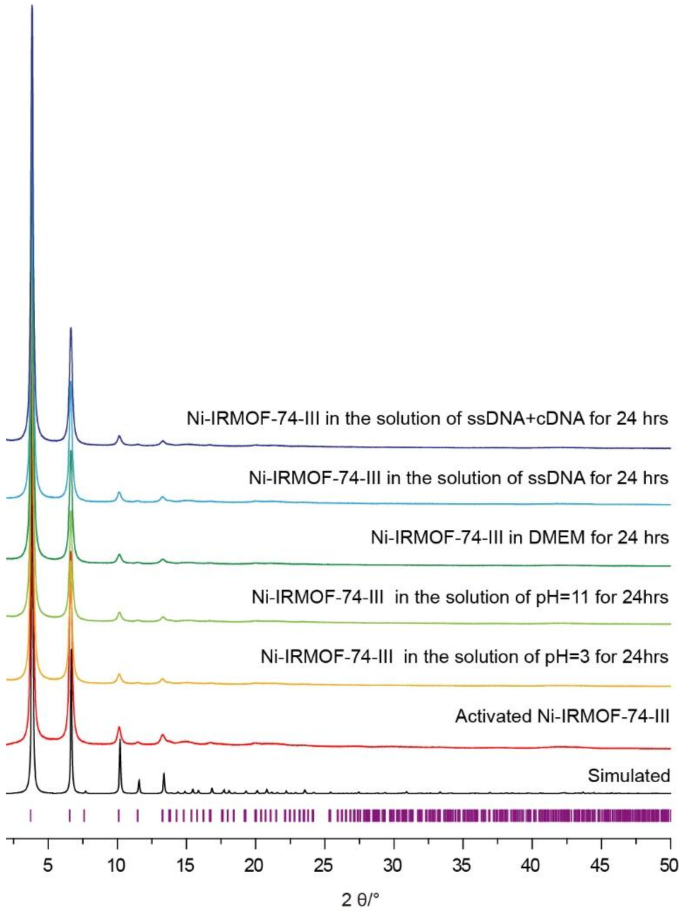

Supplementary Figure 60 Comparison of the experimental PXRD pattern of the activated Ni-IRMOF-74-III (red) with the diffraction patterns after immersion in various aqueous solution for 24 hours (Cu Kα λ = 1.5406 Å).

Stability test of Mg-IRMOF-74-II

Mg-IRMOF-74-II was synthesized and activated as reported[1]. The activated sample of Ni-IRMOF-74-III was immersed in the solution of pH = 3, pH = 11, with ssDNA, with cDNA and cell culture medium (DMEM), respectively. The samples were collected after immersion for 24 hours followed by washing with deionized water and ethanol for three times, respectively. The decrease in peak intensities and the emergence of new peak at around $2\theta = 6$ indicate the partial decomposition of MOF structure. Furthermore, most of the sample was dissolved by DMEM (containing 10% FBS) solution and the residual exhibited no peaks in PXRD, indicating a total destroy of the structure. In comparison, the chemical stability of Ni-IRMOF-74-II was much higher and thus was more suitable for the delivery of ssDNA (Figure 2b and Supplementary Figure 61).

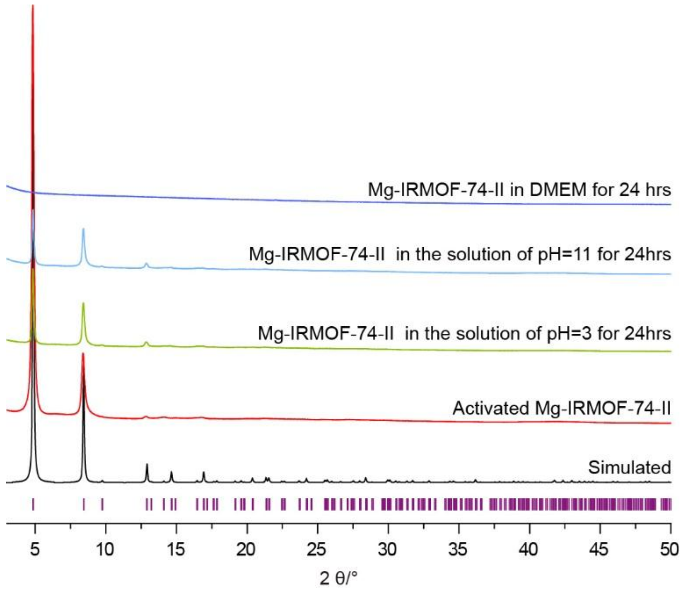

Supplementary Figure 61 Comparison of the experimental PXRD pattern of the activated Mg-IRMOF-74-II (red) with the diffraction patterns after immersion in various aqueous solution for 24 hours (Cu Kα $\lambda = 1.5406$ Å).

Mapping of DNA distribution inside MOF pores

Ni-IRMOF-74-II samples with different ssDNA loading amount were dried under vacuum for 12 h before immersion in a 10 mL solution of $Ni(NO_3)_2{\cdot}6H_2O$ (0.018 M). The actual loading amount of ssDNA in each MOF samples was listed in Supplementary Table 9. The samples were kept in the solution for half an hour to include Ni^{2+} that can coordinate with ssDNA. Then the mixture was centrifuged and washed with ionized water three times to remove the uncoordinated Ni^{2+} followed by washing with ethanol was to replace the solvent molecules inside MOFs. After exchange with ethanol for three times, these samples were evacuated (10^{-2} Torr) at room

temperature for 24 h. PXRD patterns of ssDNA loaded MOF samples were collected using synchrotron X-ray source with ssDNA free Ni-IRMOF-74-II samples as control.

We performed ICP-AES of a washed and dried sample of 6.9% wt ssDNA in Ni-IRMOF-74-II and a ratio of [Ni]/[P]=10.5 was obtained. The theoretical ratio corresponding to two phosphate group per unit cell of Ni-IRMOF-74-II is 8, which is close to the ratio of 10.5 considering the blocked space by ssDNA. This further validates the fact that 6.9% wt ssDNA in Ni-IRMOF-74-II is saturated corresponding to the saturated value in Table 1. The electron density map of ssDNA in the lattice plane (Supplementary Figure 63) is in good correlation to the electron distribution map without gradient in Figure 2. Supplementary Figure 63 emphasizes on the density variations inside the pores. With the concentration of ssDNA increase, the electron density around the walls increases and stretches to the centre of the pores, indicating the increased interaction between walls and ssDNA. The electron density on the walls of the framework might originate from the rotation of benzene rings. The unaltered pore size distribution derived from nitrogen adsorption isotherm of the ssDNA loaded MOF samples indicate the entire pore was blocked when ssDNA was full loaded (Supplementary Table 10).

Supplementary Table 9 Uptake of ssDNA at different feed ratio

MOF samples	2.3% ssDNA in Ni-IRMOF-74-II	4.7% ssDNA in Ni-IRMOF-74-II	6.9% ssDNA in Ni-IRMOF-74-II
Feed ratio (wt%)	2.5	5	10
Real ratio (wt%)	2.3	4.7	6.9

The PXRD of ssDNA loaded Ni-IRMOF-74 was collected on Rigaku Smartlab Diffractometer (Cu Kα λ = 1.5406 Å) (Supplementary Figure 62). As the loading of ssDNA increases, the intensity of 110 (hkl) peak also increases in comparison to 300 (hkl), indicating there is interaction between ssDNA and MOF pores.

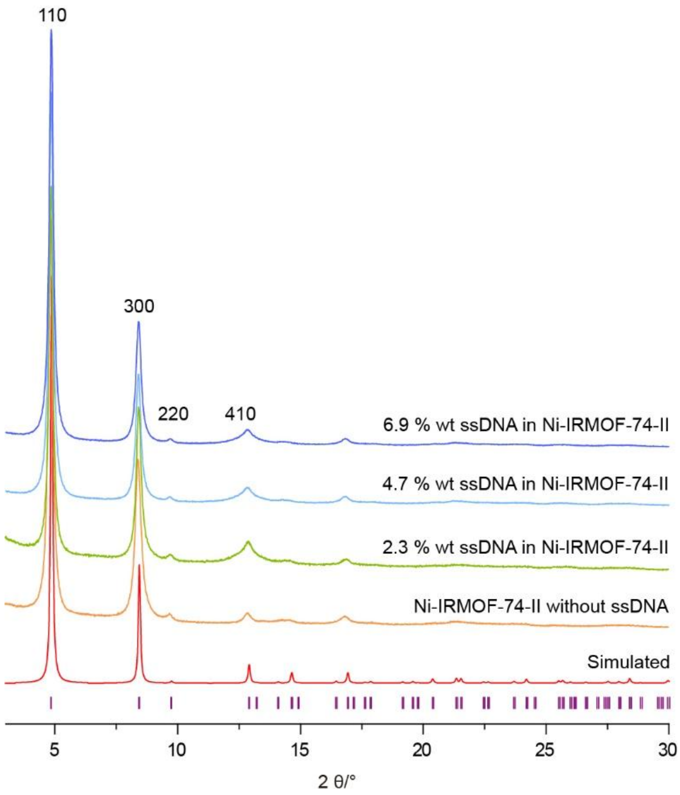

Supplementary Figure 62 Overlay of the experimental PXRD patterns of the Ni-IRMOF-74-II without ssDNA (red), 2.3% wt ssDNA in Ni-IRMOF-74-II (green), 4.7% wt ssDNA in Ni-IRMOF-74-II (light blue), 6.9% wt ssDNA in Ni-IRMOF-74-II (blue) (Cu Kα λ = 1.5406 Å).

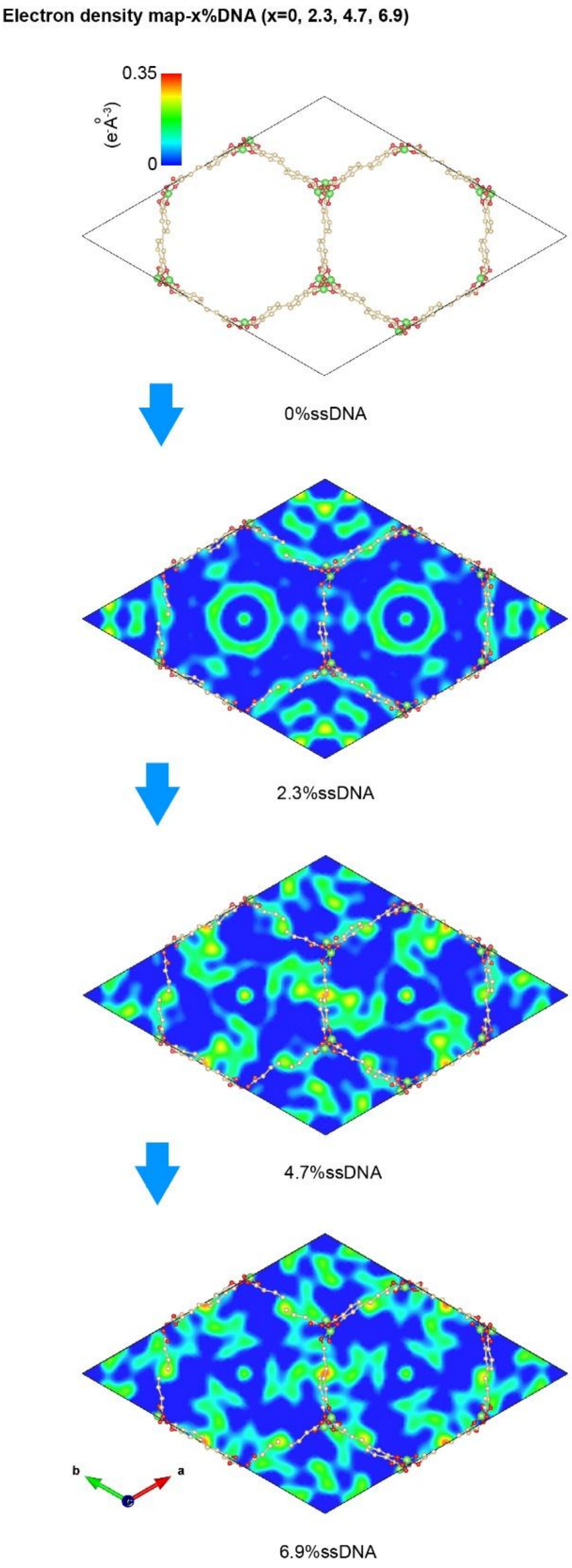

Supplementary Figure 63 Electron density maps with gradient of Ni-IRMOF-74-II with x% wt ssDNA inside pores (x=0, 2.3, 4.7, 6.9).

Activation of the ssDNA loaded Ni-IRMOF-74 samples

The ssDNA loaded samples of Ni-IRMOF-74-II and -III were washed with dry ethanol for three times before evacuated with supercritical CO_2 in a Tousimis Samdri PVT-3D critical point dryer. The samples were placed in the chamber and ethanol was gradually exchanged with liquid CO_2. After exchange with liquid CO_2 for three times, the chamber was filled fully with liquid CO_2. Then the whole chamber was heated up to about 40 °C and held constant at the supercritical condition (typically 1300 psi) for 0.5 h. CO_2 was slowly vented from the chamber at around 40 °C for about 8 hours before the pressure reached 400 psi. Later, the sample was evacuated with a dynamic vacuum (10^{-5} Torr pressure) at 100 °C for 5 h to remove the residual ethanol prior to N_2 absorption test (Supplementary Figure 64-71, Supplementary Table 10). The pore size distribution was not affected by the loading of ssDNA, indicating an excellent accommodation of ssDNA inside MOF pores. This is also in agreement with the electron density map of ssDNA in MOF pores, in which ssDNA fulfill the pores.

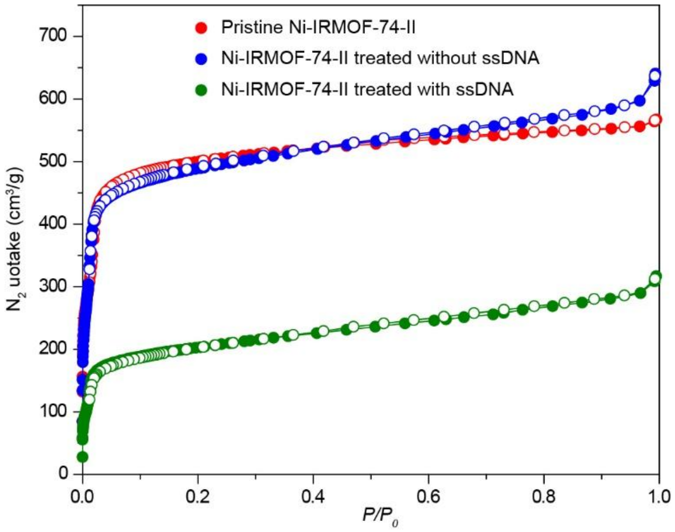

Supplementary Figure 64 Nitrogen isotherm of Ni-IRMOF-74-II (red), Ni-IRMOF-74-II treated without ssDNA (blue) and 6.9% wt ssDNA in Ni-IRMOF-74-II (green) at 77 K**.** Filled and open symbols represent absorption and desorption branches, respectively. Points are connected to provide clear shape of the isotherm.

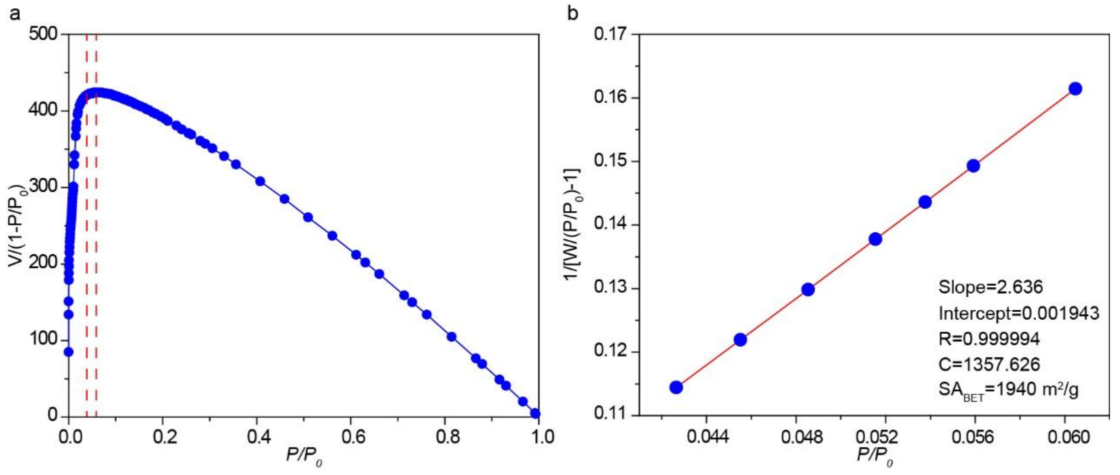

Supplementary Figure 65 BET area calculation for Ni-IRMOF-74-II treated without ssDNA from simulated nitrogen isotherm at 77 K (a) Only points between the dashed lines are selected based on the first consistency criterion, (b) Plot to select linear P/P_0 range.

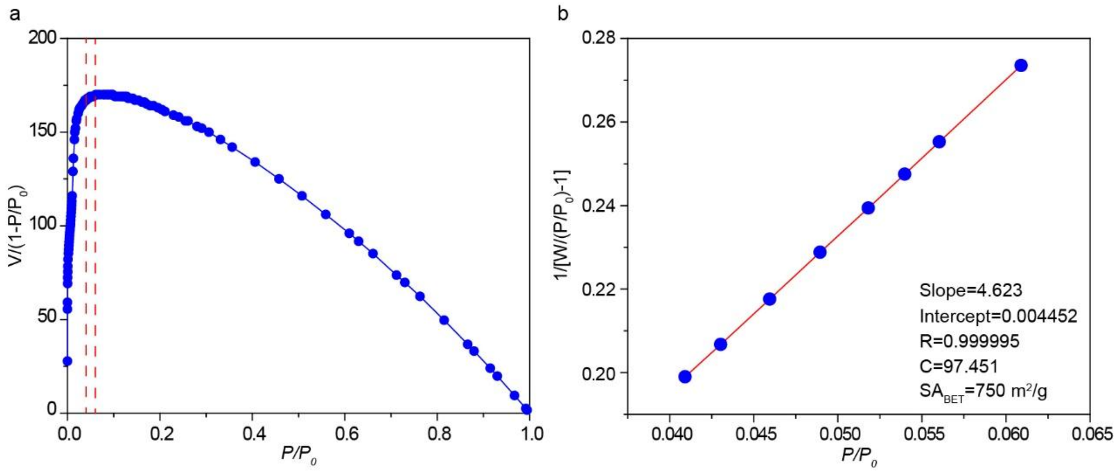

Supplementary Figure 66 BET area calculation for 6.9% wt ssDNA in Ni-IRMOF-74-II from simulated nitrogen isotherm at 77 K (a) Only points between the dashed lines are selected based on the first consistency criterion, (b) Plot to select linear P/P_0 range.

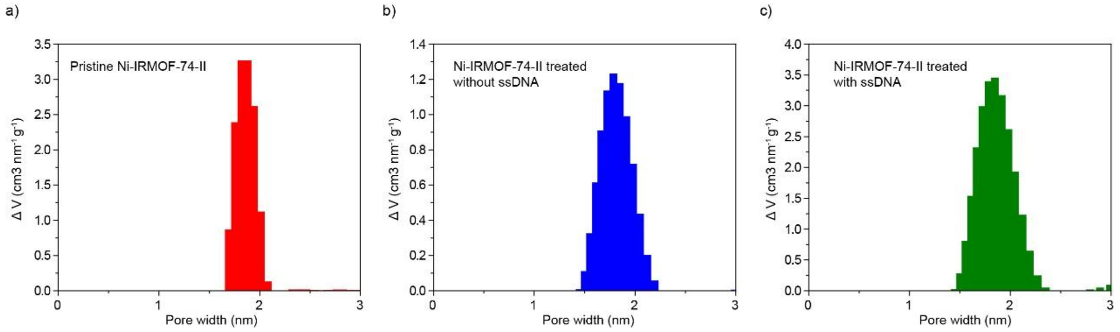

Supplementary Figure 67 Pore size distribution for Ni-IRMOF-74-II from simulated nitrogen isotherm at 77 K (a) Pristine Ni-IRMOF-74-II (b) Pristine Ni-IRMOF-74-II treated with pure water without ssDNA, (c) Pristine Ni-IRMOF-74-II treated with ssDNA aqueous solution.

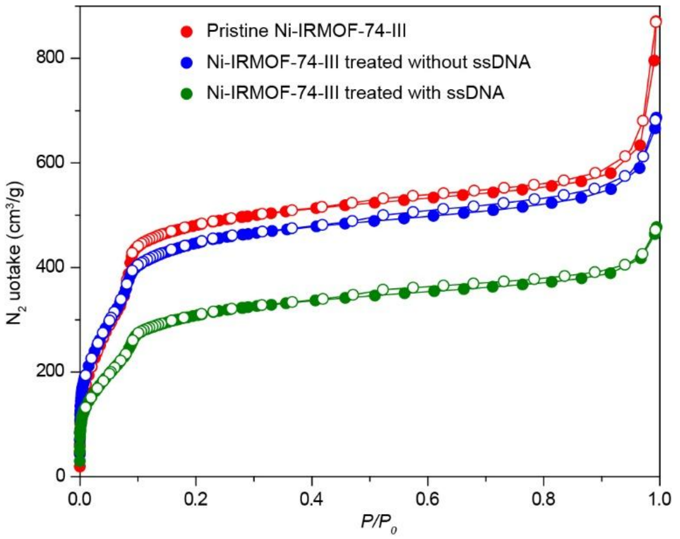

Supplementary Figure 68 Nitrogen isotherm of Ni-IRMOF-74-III (red), Ni-IRMOF-74-III treated without ssDNA (blue) and 6.9% wt ssDNA in Ni-IRMOF-74-III (green) at 77 K**.** Filled and open symbols represent absorption and desorption branches, respectively. Points are connected to provide clear shape of the isotherm.

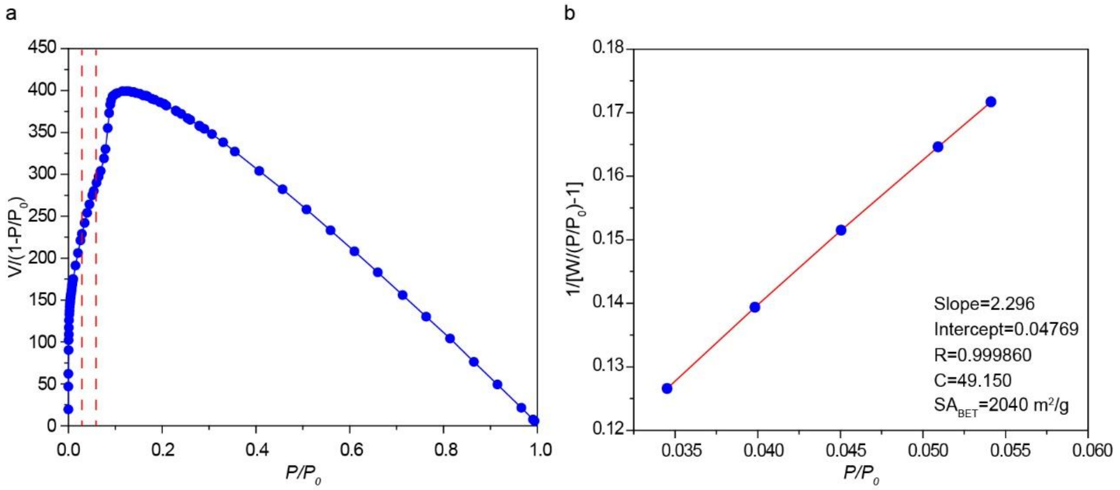

Supplementary Figure 69 BET area calculation for Ni-IRMOF-74-III treated without ssDNA from simulated nitrogen isotherm at 77 K (a) Only points between the dashed line are selected based on the first consistency criterion, (b) Plot to select linear P/P_0 range.

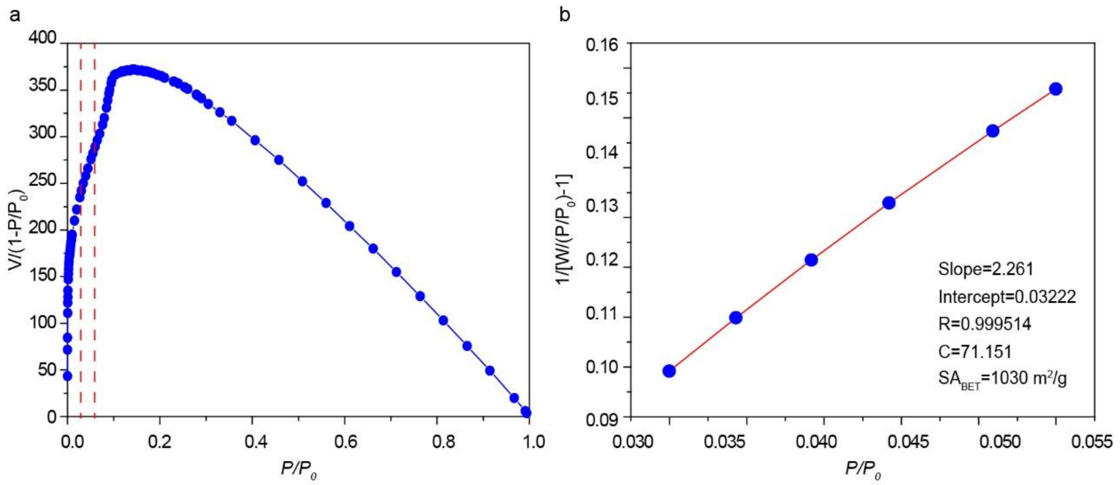

Supplementary Figure 70 BET area calculation for 6.9% wt ssDNA in Ni-IRMOF-74-III from simulated nitrogen isotherm at 77 K (a) Only points between the dashed line are selected based on the first consistency criterion, (b) Plot to select linear P/P_0 range.

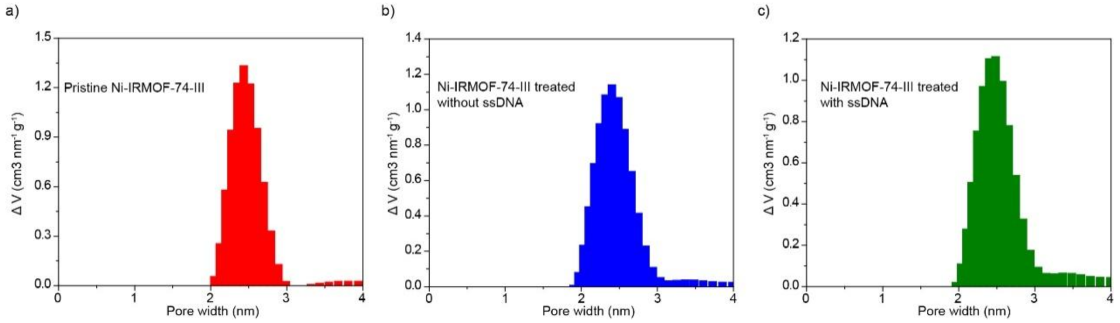

Supplementary Figure 71 Pore size distribution for Ni-IRMOF-74-III from simulated nitrogen isotherm at 77 K (a) Pristine Ni-IRMOF-74-III (b) Pristine Ni-IRMOF-74-III treated with pure water without ssDNA, (c) Pristine Ni-IRMOF-74-III treated with ssDNA aqueous solution.

Supplementary Table 10. Summary of parameters from nitrogen absorption analysis.

Compound	Framework molecular formula	BET SA (m^2/g)	Pore volume (cm^3/g)	Pore width (nm)
Ni-IRMOF-74-II without ssDNA	$C_7H_4NiO_4$	1940	0.78	1.8
6.9% wt ssDNA in Ni-IRMOF-74-II	N.A.	750	0.45	1.8
Ni-IRMOF-74-III without ssDNA	$C_{10}H_6NiO_4$	2040	0.93	2.4
6.9% wt ssDNA in Ni-IRMOF-74-III	N.A.	1030	0.61	2.4

N.A., not applicable.

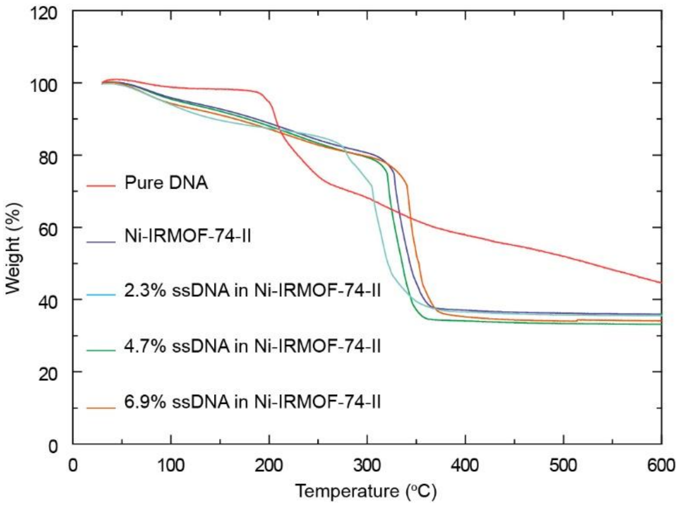

Supplementary Figure 72 TGA trace of pure DNA (blue) and Ni-IRMOF-74-II loaded with different amount of DNA. The activated Ni-IRMOF-74-II was loaded with ssDNA with 2.3% wt (light blue), 4.7% wt (green), 6.9% wt (red), respectively.

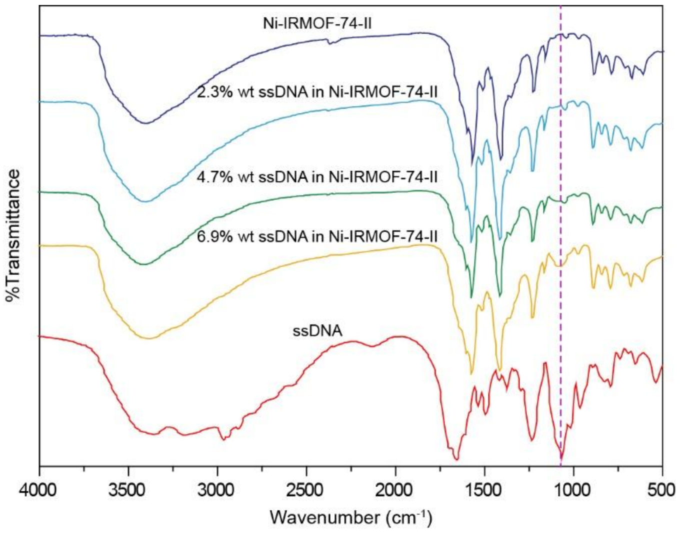

Supplementary Figure 73 IR spectra of Ni-IRMOF-74-II, Ni-IRMOF-74-II -2.5 (2.3)% ssDNA, The activated Ni-IRMOF-74-II was loaded with ssDNA with 2.3% wt (light blue), 4.7% wt (green), 6.9% wt (red), respectively.

Supplementary Method 4.

Quantification of the Uptake and Release of ssDNA using MOF as Vectors through Fluorescence Measurement

General method

Preparation of MOF and ssDNA storage solution

MOF solution was prepared by dispersing 2 mg of MOF samples in 1 mL double-distilled H_2O (ddH_2O) by sonication. ssDNA solution was prepared by dissolving ssDNA in ddH_2O at the concentration of 100 µM, and then diluted to 1µM.

Supplementary Table 11 Sequences of DNA used in this study

Name	Sequence (from 5' end to 3' end)
FAM-ssDNA	FAM-CCGCGGCCAGGCTAGCTACAACGACCTGGACGA
cDNA	TCGTCCAGGTCGTTGTAGCTAGCCTGGCCGCGG
Doubly labeled ssDNA	FAM-CCGCGGCCAGGCTAGCTACAACGACCTGGACGA-
Substrate DNA	TCG TCC AGG rArU GGC CGC GG
SC-DNA	ATCGAATAGTCTGACTACAACT
Control DNA	CCGCGGCCAGGCTACCTACAACGACCTGGACGA
DNAzyme	CCGCGGCCAGGCTAGCTACAACGACCTGGACGA
FAM-SC-DNA	FAM-ATCGAATAGTCTGACTACAACT
FAM-control DNA	FAM-CCGCGGCCAGGCTACCTACAACGACCTGGACGA
FAM-DNAzyme	FAM-CCGCGGCCAGGCTAGCTACAACGACCTGGACGA
53nt	FAM-
33nt	FAM-CCGCGGCCAGGCTAGCTACAACGACCTGGACGA
22nt	FAM-ATCGAATAGTCTGACTACAACT
11nt	FAM-GGTTGGTGTGG
F-EGR-1 primer	AAC AGT GGC AAC ACC TTG TG
R-EGR-1 primer	ACT GGT AGC TGG TAT TGA GG
F- β-action primer	CACGATGGAGGGGCCGGACTCATC
R- β-action primer	TAAAGACCTCTATGCCAACACAGT
EGR-1 siRNA sense	GCAGACAAAAGUGUUGUGGtt
EGR-1 siRNA anti-sense	CCACAACACUUUUGUCUGCtt

All oligonucleotides above were purchased from Sangon Biotechnology Co., Ltd. (Shanghai, China). DNA concentration was quantified by NanoDrop 2000c (Thermo Scientific, USA). All water used in this study was ultrapure water (18.2 MΩ/cm). The

cationic polymer, Neofect DNA transfection reagent was bought from NEOFECT Gene Delivery Biosystems Company. LipoGene 2000 Plus Transfection Reagent (Lipo) was purchased from Everbright Inc.USA.

Fluorescence measurement

The fluorescence intensity (FI) of the sample is determined by using a LS55 fluorescence spectrometer (Perkin-Elmer Inc, USA). For 6-carboxylfuorescein (FAM), excitation and emission wavelengths were set at 480 nm and 520 nm, respectively. The emission spectra were collected from 500 to 600 nm using the excitation wavelength of 480 nm, and the fluorescence intensity at 520 nm was used for analysis. For 6-carboxytetramethylrhodamine (TAMRA), the excitation and emission wavelengths of were set at 550 nm and 580 nm, respectively. The emission spectra were collected from 555 to 650 nm using the excitation wavelength of 550 nm, and the fluorescence intensity at 580 nm was used for analysis. The detection is performed at room temperature with a 1 cm-path-length cell and in the buffer solution (50 mM Tris-HCl, pH = 8.0 and 5 mM $MgCl_2$).

Quantification method of ssDNA

The quantification of ssDNA uptake in MOFs is obtained by the fluorescence decay of FAM labeled ssDNA in the solution. The release of the included ssDNA from the pores can be quantified based on the fluorescence recovery by adding complementary DNA (cDNA) to the solution to form dsDNA. In order to find the most appropriate conditions for the uptake of ssDNA, several experiments were carried out to explore the proper feed ratio, ionic strength and experimental amounts (Supplementary Figure 74-82).

Exploration of the appropriate feed ratio of Ni-IRMOF-74-II and ssDNA

25 μL of ssDNA (1 μM) were separately mixed with 2 μL, 4 μL, 6 μL, 8 μL, 10 μL of Ni-IRMOF-74-II (2 mg/mL) in 250 μL aqueous solution (50 mM Tris-HCl, pH = 8.0 and 5 mM $MgCl_2$) under 1000 rpm vibration at 37 ℃ for 3 h, respectively. The final concentrations of Ni-IRMOF-74-II were: 16 μg/ mL, 32 μg/ mL, 48 μg/ mL, 64 μg/ mL, 80 μg/ mL, respectively. And the fluorescence intensity (FI) of the solution was measured at 520 nm. The uptake value of ssDNA was obtained using the following formula[11] (Supplementary Equation 1, $FI_{original}$ is the fluorescence intensity of pure FAM labeled ssDNA in the buffer solution without any other vectors). Supplementary Figure 74 illustrated that 48 μg/mL Ni-IRMOF-74-II is the most appropriate concentration for the uptake of 100 nM of ssDNA, because it is close to the saturated uptake of ssDNA, and no genetic materials are wasted.

$$adsorption(\%) = \frac{FI_{original} - FI}{FI_{original}} \times 100\% \qquad (1)$$

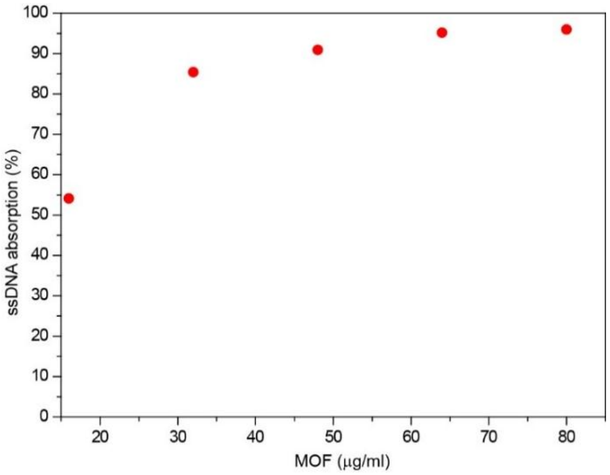

Supplementary Figure 74 Uptake of ssDNA (100 nM) by a series of concentrations of Ni-IRMOF-74-II.

Exploration of ionic strength for ssDNA inclusion

25 μL of ssDNA (1 μM) and 6 μL of Ni-IRMOF-74-II (2 mg/ml) were mixed in 250 μL aqueous solution (50 mM Tris-HCl, pH = 8.0). A series of $MgCl_2$ concentrations (0 mM, 1 mM, 5 mM, and 10 mM) were added to the complex of ssDNA and Ni-IRMOF-74-II and incubated under 1000 rpm vibration at 37 ℃ for 3 h. And FI of the solution was measured at 520 nm (Supplementary Figure 75). The decrease of FI indicated that high salt concentration of Mg^{2+} could promote the uptake of ssDNA by Ni-IRMOF-74-II. The 5 mM of $MgCl_2$ was chosen as the proper ionic strength in following experiment because it is the lowest concentration of $MgCl_2$ to ensure the uptake of almost all ssDNA with the least interference.

$$FI(\%) = \frac{FI}{FI_{original}} \times 100\% \qquad (2)$$

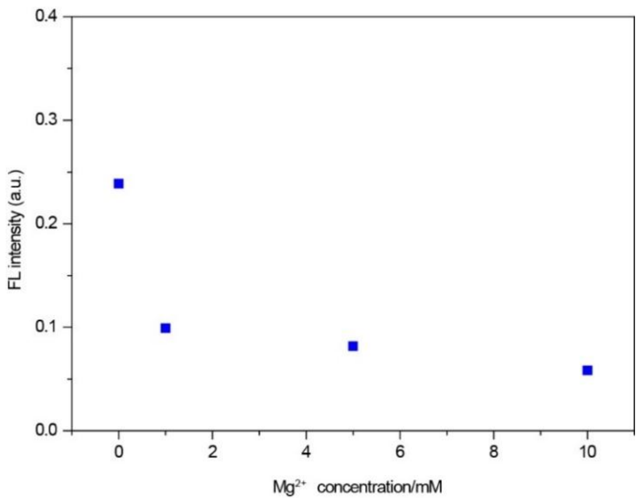

Supplementary Figure 75 ssDNA inclusion in Ni-IRMOF-74-II in $MgCl_2$ solutions of different concentrations.

Exploration of the ideal ration between cDNA and ssDNA loaded MOF samples for the recovery test

Different amount of the ssDNA labed MOF samples were added into a cDNA solution of fixed concentration (100 nM) (Supplementary Table 12).

Supplementary Table 12. Stoichiometry of ssDNA loaded MOFs and cDNA.

Number of reaction	A1	A2	A3	A4	A5	A6	A7
ssDNA (nM)	5	25	50	75	100	200	300
Ni-IRMOF-74-II (μg/mL)	2.4	12	24	36	48	96	144
cDNA (nM)	100	100	100	100	100	100	100
Number of reaction	C1	C2	C3	C4	C5	C6	C7
SsDNA (nM)	5	25	50	75	100	200	300
Ni-IRMOF-74-II (μg/mL)	0	0	0	0	0	0	0
cDNA (nM)	100	100	100	100	100	100	100

ssDNA and the corresponding Ni-IRMOF-74-II in Supplementary Table 12 were mixed. Then the mixture was diluted to 250 μL aqueous solution to form ssDNA loaded MOF samples (50 mM Tris-HCl, pH = 8.0 and 5 mM $MgCl_2$) and then incubated under 1000 rpm vibration at 37 ℃ for 3 h. FI of the solution was measured at 520 nm (FI_{quench}). Then 100 nM of cDNA was added and allowed to react under 1000 rpm vibration at 37 ℃ for 5 h. Fluorescence intensity (FI_A, FI_C) of the solution was measured at 520 nm (Supplementary Figure 76). This figure illustrated that 100 nM of cDNA could make the 50 nM of ssDNA loaded MOF samples reach the highest release efficiency.

$$FL\ recovery(\%) = \frac{FI_{A_n} - FL_{quench_n}}{FI_{C_n}} \times 100\% \qquad (3)$$

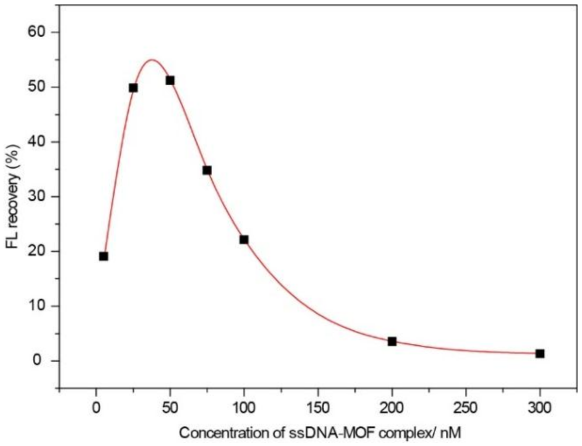

Supplementary Figure 76 Fluorescence recovery of ssDNA loaded MOF samples after addition of cDNA.

Fluorescence recovery

25 μL of ssDNA (1 μM) and 6 μL Ni-IRMOF-74-II (2 mg/mL) were mixed. Then the mixture was diluted to 500 μL aqueous solution (50 mM Tris-HCl, pH = 8.0 and 5 mM $MgCl_2$) and incubated at 37 ℃ for 3 h. Fluorescence intensity (FI) of the solution (FI_{quench}) was measured at 520 nm. Then different concentrations of cDNA reacted with above mixture solution under 1000 rpm vibration at 37 ℃ for 3 h, followed by detection of FI of the solution at 520 nm (Supplementary Figure 77). In the range of 3.125 nM to 50 nM of cDNA concentration, FI and cDNA concentration have great linear relationship (adjust R-square = 0.99812).

$$FL\ recovery\ intensity = FI_{original} - FI \qquad (4)$$

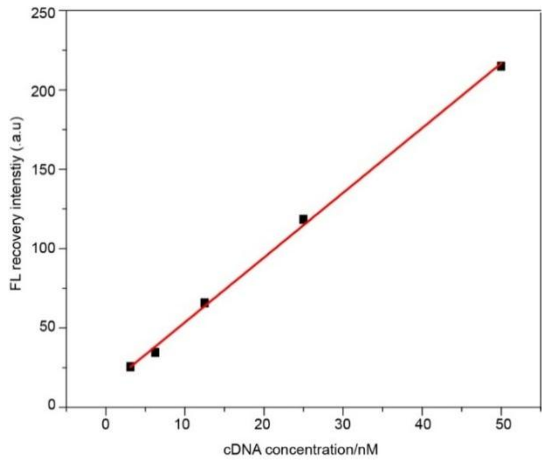

Supplementary Figure 77 Fluorescence recovery of ssDNA from MOF pores in the present of cDNA.

Uptake of FAM-labeled ssDNA by other MOF vectors

15.5 μmol of each MOF vectors (UiO-66, UiO-67 and MIL-101-Cr) and 12.5 μL of ssDNA (1 μM) were separately mixed. Then the mixture was diluted to 250 μL aqueous solution (50 mM Tris-HCl, pH = 8.0 and 5 mM $MgCl_2$) and incubated under 1000 rpm vibration at 37 °C, respectively. FI was detected at different time points (FI_t). Supplementary Figure 78 showed that MIL-101-Cr can remove almost all of the ssDNA from the solution, while UiO-66 and UiO-67 show relatively low uptake, probably just through surface binding. The molecular weight of these vectors was applied as reported[49-51]. The molecular weights of Ni-MOF-74, Ni-IRMOF-74-II, -III, -IV, -V, UiO-66, UiO-67 and MIL-101-Cr are: 311 g/mol, 388 g/mol, 491 g/mol, 596 g/mol, 700 g/mol, 532 g/mol, 707 g/mol, 637 g/mol, respectively.

$$ssDNA\ adsorption(\%) = \frac{FI_{original} - FI_t}{FI_{original}} \times 100\% \qquad (5)$$

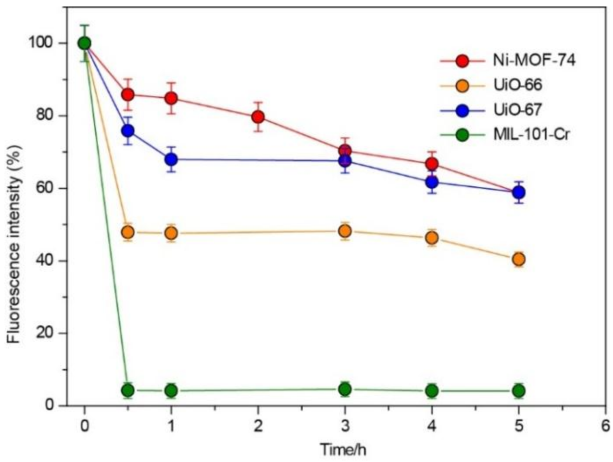

Supplementary Figure 78 Fluorescence quench of FAM labeled ssDNA by Ni-MOF-74, UiO-66, UiO-67 and MIL-101-Cr, respectively (n = 3 technical replicates; bars represent mean ± s.d.).

Control experiment of FAM labeled ssDNA uptake using structural components of Ni-IRMOF-74-II

15.5 μmol of metal ions, liner II, physical mixture of metal ions and liner II, and Ni-IRMOF-74-II were mixed with 12.5 μL of FAM-labeled ssDNA (1 μM). Then the mixture was diluted to 250 μL aqueous solution (50 mM Tris-HCl, pH = 8.0 and 5 mM $MgCl_2$) and incubated under 1000 rpm vibration at 37 °C, respectively. The significant decrease of FI was observed only in Ni-IRMOF-74-II. This indicated that the topology of Ni-IRMOF-74 is the main factor for the uptake of ssDNA in comparison to single or mixed components of this MOF (Supplementary Figure 79). And pure MOFs were also tested to ensure the influence on fluorescence by themselves (Supplementary Figure 80). The results of fluorescence are nearly zero, indicating a no interference for fluorescence.

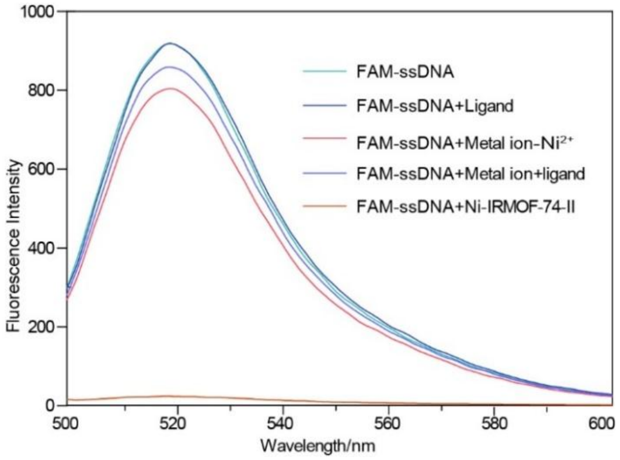

Supplementary Figure 79 Fluorescence quench by equivalent molar amounts (62 μM) of metal ions, organic ligand, physical mixture of metal ions and organic ligand, and Ni-IRMOF-74-II. Excitation was applied at 480 nm and emission at 520 nm.

Additionally, in order to eliminate the interference of the MOF itself, the fluorescence of MOF itself was test (Supplementary Figure 80). We could notice that there is almost no fluorescence was observed in the pure MOFs, indicating there is no residual fluorescence from MOFs which is in agreement with its structure built from non-planar benzene rings. To sum up, in the cell-uptake studies, the intracellular fluorescence comes from the FAM-ssDNA transfected by the MOF vector.

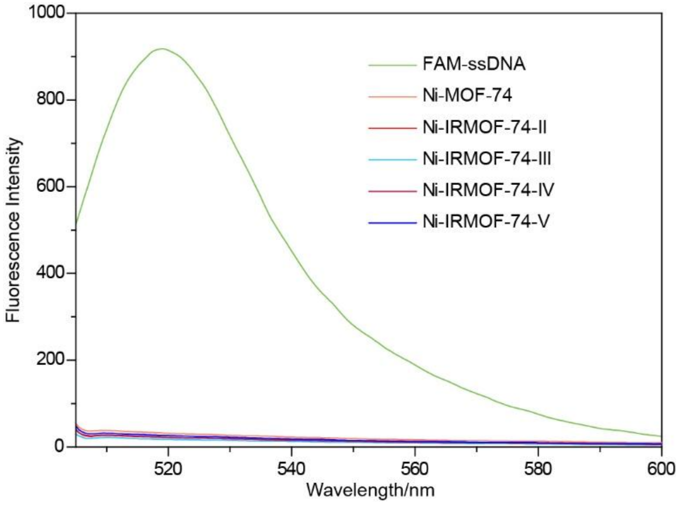

Supplementary Figure 80 Fluorescence of FAM-ssDNA, Ni-MOF-74, Ni-IRMOF-74-II to -V.

Uptake of ssDNA and dsDNA

FAM labeled ssDNA and cDNA was mixed and diluted to 1 mL aqueous solution (50 mM Tris-HCl, pH = 8.0 and 5 mM $MgCl_2$, FAM labeled ssDNA (1 μM) and cDNA (1 μM)). Then the solution was heated to 95 °C for 10 min in water bath, and cooled down to room temperature. FAM labeled ssDNA and cDNA were annealed to form dsDNA, which is the dsDNA storage solution.

15.5 μmol of Ni-MOF-74, Ni-IRMOF-74-II, Ni-IRMOF-74-III, Ni-IRMOF-74-IV, Ni-IRMOF-74-V and MIL-101-Cr samples were separately mixed with 12.5 μL of ssDNA (1 μM) and 12.5 μL of dsDNA (1 μM). Then the mixture was diluted to 250 μL aqueous solution (50 mM Tris-HCl, pH = 8.0 and 5 mM $MgCl_2$) and incubated under 1000 rpm vibration at 37 °C for 3 h. FI was detected at 520 nm (Supplementary Figure 81) and converted to uptake efficiency using the following equations (Supplementary Equation 6) and Supplementary Equation 7).

$$ssDNA\ uptake\ efficency(\%) = \frac{FI_{original\text{-}ss} - FI_{ss}}{FI_{original\text{-}ss}} \times 100\% \qquad (6)$$

$$dsDNA\ uptake\ efficency(\%) = \frac{FI_{original\text{-}ds} - FI_{ds}}{FI_{original\text{-}ds}} \times 100\% \qquad (7)$$

Supplementary Figure 81 shows that Ni-MOF-74 can neither include ssDNA nor dsDNA due to its small pore size. While Ni-IRMOF-74-II, -III, -IV, and -V show more than 90% absorption efficiency for ssDNA, and a gradually increasing absorption efficiency for dsDNA with the increasing the pore size. The reason is that ssDNA has a smaller diameter and more flexible chain so that it is easier to adjust to the pore of Ni-IRMOF-74-II to -V. Another reason is that ssDNA has exposed bases[12, 13], thus the ssDNA can readily enter the pore and interact closely with the interior surface of Ni-IRMOF-74-II to -V through multiple noncovalent interactions. However, double-stranded DNA has a rigid conformation without unpaired bases, thus has less uptake in comparison to ssDNA. The difference in uptake amount and interactions provides a good selectivity for ssDNA from dsDNA in Ni-IRMOF-74-II, in which the pore size is slightly smaller than the diameter of dsDNA. In addition, MIL-101-Cr can't distinguish the ssDNA and dsDNA due to a much stronger interaction between DNA and MOF pores (Supplementary Figure 81).

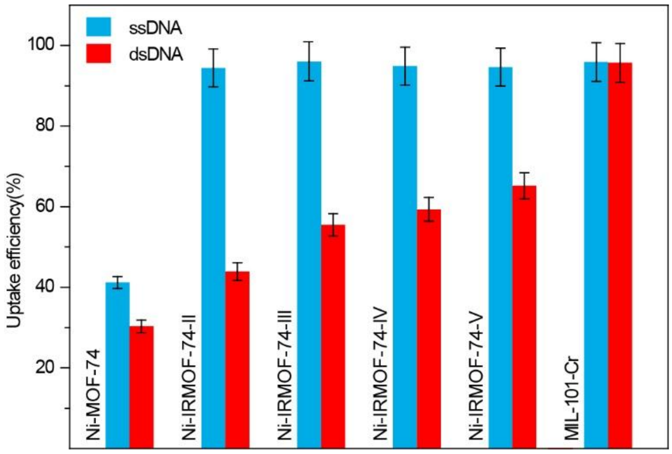

Supplementary Figure 81 Uptake of ssDNA and dsDNA by different MOF vectors (n = 3 technical replicates; bars represent mean ± s.d.).

Uptake of different length of ssDNA by Ni-IRMOF-74-II

Four ssDNAs (11 nt, 22 nt, 33 nt and 53 nt) labeled with FAM were chosen in this study (sequences refer to Supplementary Table 11). 3 μL of Ni-IRMOF-74-II (2 mg/mL) and 12.5 μL of each ssDNA (1 μM) follow the same operations above. Supplementary Figure 82 showed that all these four ssDNAs can be included into Ni-IRMOF-74-II, demonstrating that the inclusion of ssDNA is not limited by its length.

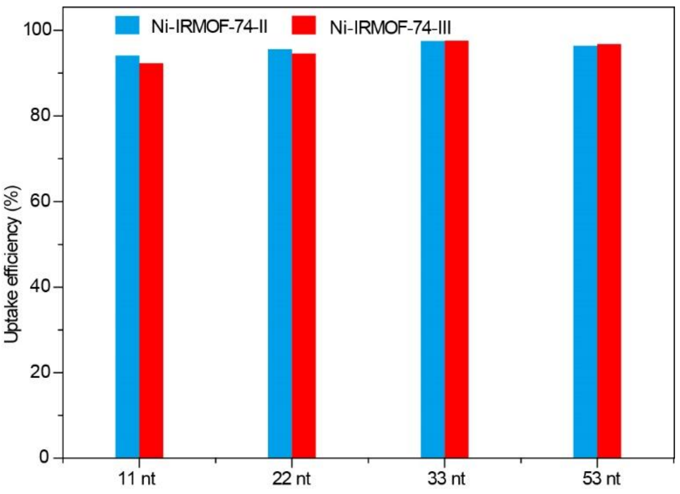

Supplementary Figure 82 Uptake of ssDNA with different length by Ni-IRMOF-74-II and Ni-IRMOF-74-III.

Uptake of siRNA

360 μg/mL of Ni-IRMOF-74-III, -IV, -V were incubated with siRNA (125 nM) under 1000 rpm vibration at 37 °C for 3 h, respectively. siRNA uptake efficiency was detected followed the same procedure above through 20% native polyacrylamide gel electrophoresis (PAGE). In the siRNA inclusion test (Supplementary Figure 83), the absorption increased as the pore size increases. And this experiment has demonstrated the possibility for the uptake of siRNA.

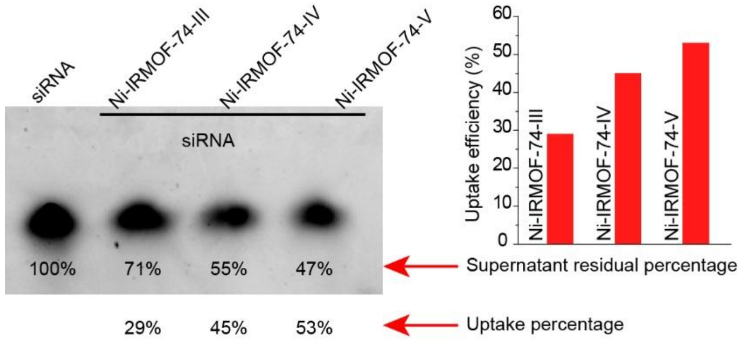

Supplementary Figure 83 Uptake of siRNA in Ni-IRMOF-74-III to -V by electrophoresis.

Displacement experiment for release mechanism

Excess FAM-ssDNA (100 nM) was added to two groups of Ni-IRMOF-74-II (32 μg/mL) under 1000 rpm vibration at 37 °C for 2 h respectively. A non-complementary ssDNA with 22 nucleotides (100 nM) was incubated with above mixture for 3h. And cDNA was added to the other group of Ni-IRMOF-74-II saturated by FAM-ssDNA. The fluorescence of two solutions was detected respectively.

In the release system, a control experiment using oligos of non-complementary sequence (sc-DNA) was performed in contrast with cDNA. First, excess FAM labeled

ssDNA was added to two groups of Ni-IRMOF-74-II until its saturated absorption respectively. Then, both groups are washed to remove the free ssDNA. After removal of free ssDNA, the cDNA was added to one group and equal molar amount of non-complementary DNA was mixed with the other group. Finally, the fluorescence signal was collected after 4.5h with the results shown below (Supplementary Figure 84). We found that the recovery amount for the cDNA was 55%, while the non-complementary system showed less than 5% recovery efficiency, which is much lower than that of the cDNA system. If the release were displacement of ssDNA, the release of these two groups would have been very close. The result showed otherwise, and it is in good agreement with the pair-up mechanism for ssDNA release in contact with cDNA, as we proposed. So the release efficiency of ssDNA in Ni-IRMOF-74-II is much more likely to be base-pairing interactions rather than displacement of ssDNA with cDNA.

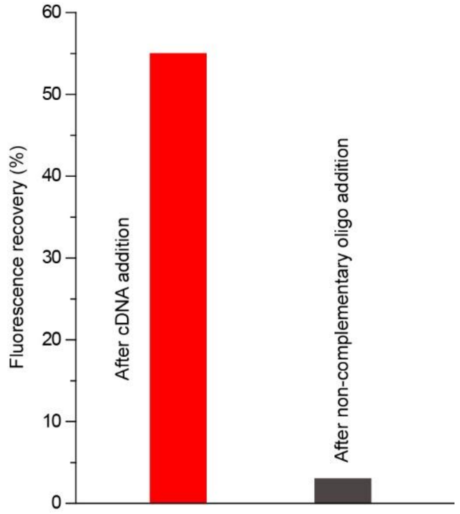

Supplementary Figure 84 Fluorescence recovery of ssDNA in Ni-IRMOF-74-II.

ssDNA inclusion in nanocrystals of Ni-IRMOF-74-II

The particle size of Ni-IRMOF-74 nanocrystals was 146.4 ± 56.85 nm (PDI = 0.151) as shown in DLS measurement (Supplementary Figure 19). In the ssDNA inclusion test using nano-MOF, the mixture of nano-MOF and FAM labeled ssDNA was diluted to 250 μL aqueous solution (nano-MOF, 24 μg/mL, FAM labeled ssDNA, 50 nM) under 1000 rpm vibration at 37 °C for 3 h. Fluorescence of the solution was measured at different point of time (Supplementary Figure 85). Different concentrations of cDNA were used in the recovery experiment. Fluorescence of the solution was measured after incubation with for 3 h for each concentration (Supplementary Figure 86).

As Ni-IRMOF-74-II crystal size scaled down to the nanometer region, fluorescence quench and recovery can also be observed with a much faster quenching rate in

comparison to regular sized MOFs. Complete quenching was achieved within 20 s, which can be attributed to the increased particle surface and shorter diffusion path (Supplementary Figure 85). In the range of 0 nM to 100 nM, the recovery of fluorescence intensity was correlated with the cDNA concentration (Supplementary Figure 86). Nano-sized MOFs were known to exhibit low toxicity and good biocompatibility, thus they are ready to be applied in *in vivo* test[14-15].

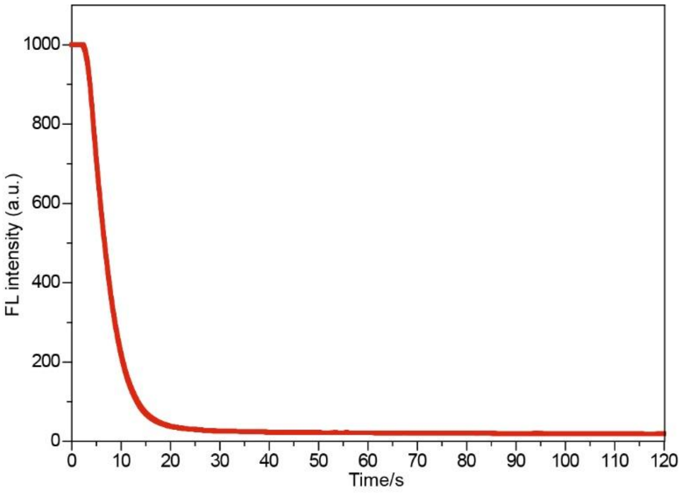

Supplementary Figure 85 Fluorescence quench of FAM labeled ssDNA by nano-MOF.

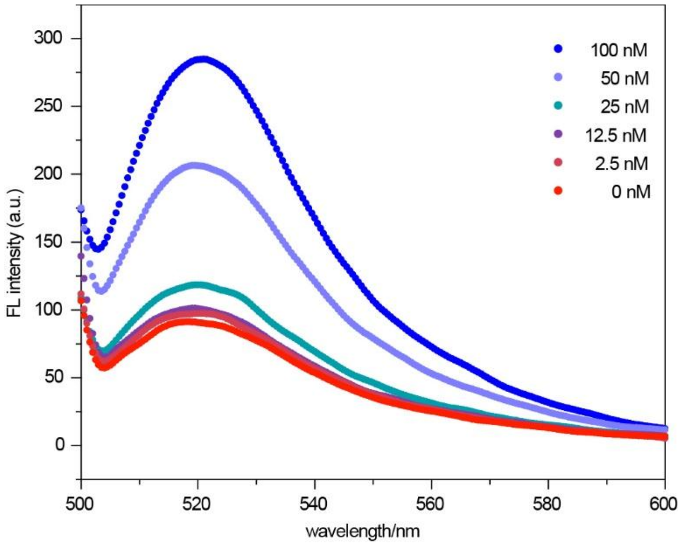

Supplementary Figure 86 Releasing efficiency of FAM-ssDNA from nano-MOF by cDNA (concentrations of cDNA, 0, 2.5, 12.5, 25, 50 and 100 nM).

Supplementary Table 13. Summary of *in vitro* test and performances of Ni-IRMOF-74 series, MIL-101-Cr, UiO-66 and UiO-67.

MOF	Pore style	Pore size/nm	Uptake ssDNA efficiency (%)	Efficiency of DNA released by cDNA (%)	Efficiency of DNA released by heat (%)	FAM Uptake efficiency (%)	TAMRA Uptake efficiency (%)	Main Interaction type
Ni-MOF-74	2D	1.1	41.2	2.6	N.A.	0	0	Surface binding
Ni-IRMOF-74-II	2D	1.8	94.4	55.0	24.5	0	4.0	van der Waals force
Ni-IRMOF-74-III	2D	2.4	96.0	24.7	1.2	0	4.6	van der Waals force, π-π stacking
Ni-IRMOF-74-IV	2D	3.0	94.9	17.8	0	0	29.1	van der Waals force, π-π stacking
Ni-IRMOF-74-V	2D	3.6	94.6	20.3	0.7	2	64.5	van der Waals force, π-π stacking
UiO-66	3D	0.6	59.6	0.5	N.A.	0	0	Surface binding
UiO-67	3D	0.8	41.1	10.8	N.A.	0	0	Surface binding
MIL-101-Cr	3D	3.0~3.4	95.9	9.9	N.A.	53.9	5.9	Electrostatic force

N.A., not applicable

.

Supplementary Method 5.
Comparison of ssDNA Protection Performance using Various Non-viral Vectors by Gel Electrophoresis

Preparation of ssDNA in vectors

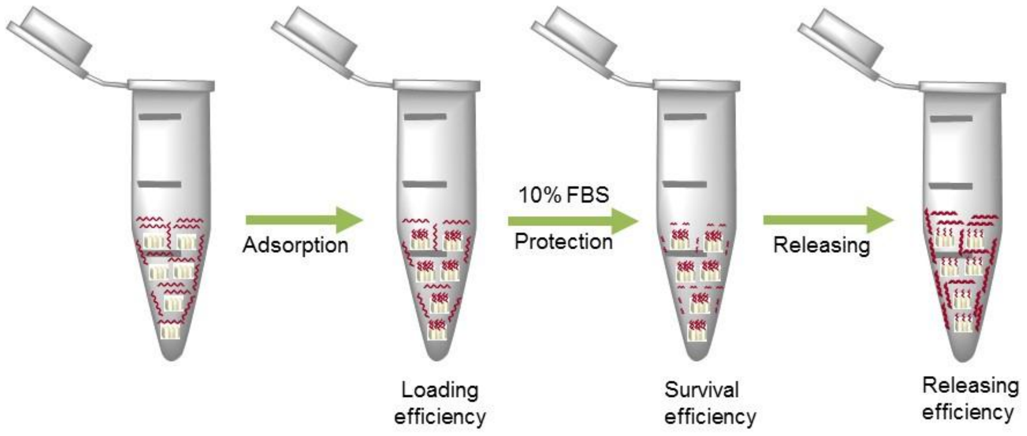

Supplementary Figure 87 Three critical steps for ssDNA delivery, loading, protection and release.

In this experiment, the protocol of ssDNA uptake into Lipo and cationic polymer (Neofect) were described in the method part in main text. The method of ssDNA uptake by Lipo was described as the following. FAM labeled ssDNA (0.272 μg) was diluted to 50 μL solution (50 mM Tris-HCl, pH = 8.0 and 5 mM $MgCl_2$) and Lipo regent (0.4 μL) was diluted to 50 μL solution (50 mM Tris-HCl, pH = 8.0 and 5 mM $MgCl_2$), respectively. Then two solutions were mixed and incubated at room temperature for 20 min. Finally, the mixture was diluted to 200 μL with double-distilled H_2O (ddH_2O).

The method of ssDNA uptake by cationic polymer (Neofect) was described as the following. 0.272 μg FAM labeled ssDNA was diluted to 100 μL solution (50 mM Tris-HCl, pH = 8.0 and 5 mM $MgCl_2$), then 0.27 μL Neofect regent was added. The two solutions were mixed and incubated at room temperature for 15 min. Finally, the mixture was diluted to 200 μL with ddH_2O.

For the other materials, Ni-IRMOF-74-II, UiO-66, UiO-67, MIL-101-Cr, and mesoporous silica (SBA-15, a common mesoporous silica with pore size of 6 nm), FAM labeled ssDNA (0.272 μg) was separately incubated with equal amounts of materials (48 μg/ mL) in 200 μL aqueous solution (50 mM Tris-HCl, pH = 8.0 and 5 mM Mg^{2+}) under 1000 rpm vibration at 37 ℃ for 3 h.

Release of ssDNA from vectors

All above materials loaded with ssDNA were centrifuged under 12,000 rpm for 15 min and 50 μL supernatant (supernatant$_{ssDNA}$) was taken out as reference for polyacrylamide gel electrophoresis (PAGE) test. Then the residual solution was suspended again followed by the addition of 400 nM cDNA, and the solution was incubated under 1000 rpm vibration at 37 °C for 5 h. After being centrifuged under 12000 rpm for 15 min, 50 μL supernatant (supernatant$_{dsDNA}$) was taken out as reference for PAGE test. Finally, 8.3 μL 6x loading buffer was added to 50 μL supernatant$_{absorb}$ and supernatant$_{release}$ separately. 6 μL of the above mixture was loaded into 12% native polyacrylamide gel, and electrophoresis was run at 250 V for 3 hours. The acrylamide concentration of the separating gel was 20 % (19: 1 monomer to bis ratio). Then the polyacrylamide gel was scanned with a Pharos FX Molecular imager (Bio-Rad, USA) operated in the fluorescence mode and FAM labeled ssDNA content was quantified by BIO-RAD quantity software. Naked FAM labeled ssDNA in solution served as control (Supplementary Figure 88).

$$releasing\ efficiency\ by\ cDNA\ (\%) = \left(\frac{FI_{supenantate_{dsDNA}}}{FI_{control}} - \frac{FI_{supenantate_{ssDNA}}}{FI_{control}} \right) \times 100\% \quad (8)$$

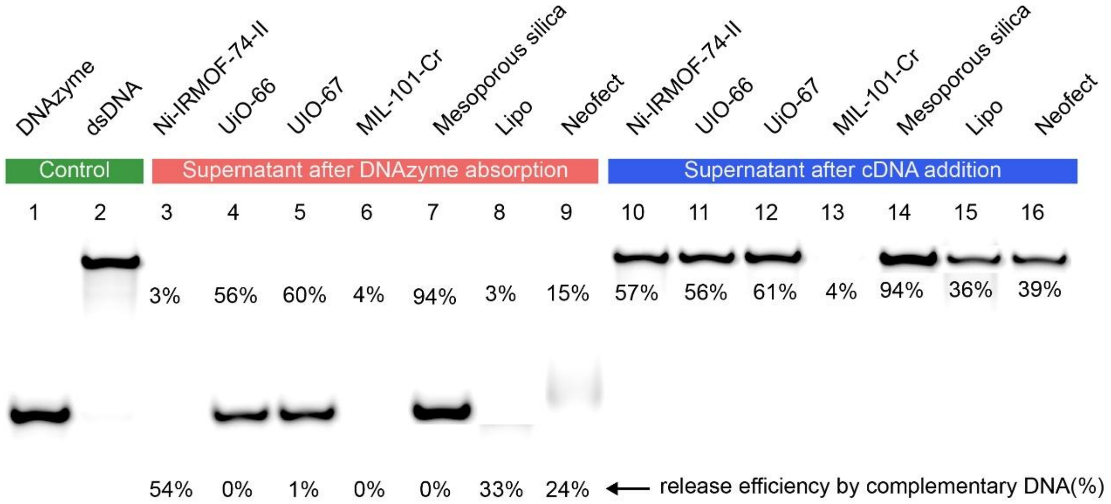

Supplementary Figure 88 Release efficiency of ssDNA by cDNA was detected through 12% native polyacrylamide gel electrophoresis (PAGE)

In the PAGE analysis (Supplementary Figure 88), the fluorescence of supernatant was detected to measure how much ssDNA was not absorbed by vectors. And the number of the first line number means that 3%, 56%, 60%, 4%, 94%, 3% and 15% of ssDNA was still free in solution after adding different vectors (Ni-IRMOF-74-II, UiO-66, UiO-67, MIL-101-Cr, mesoporous silica, Lipo and Neofect, respectively) to the solution of ssDNA respectively. So we could calculate how much ssDNA was taken by each vectors: 97% in Ni-IRMOF-74-II, 44% in UiO-66, 40% in UiO-67, 96% in MIL-

101-Cr, 6% in mesoporous silica, 97% in Lipo and 85% in Neofect. Then cDNA was added to the mixture of vectors with ssDNA with part of the fluorescence recovery, indicating a release of ssDNA from vectors to supernatant. And the fluorescence in supernatant was measured to quantify how much ssDNA was released by minus the residual ssDNA before adding cDNA, that is:

for Ni-IRMOF-74-II, 57%-3%=54%

for UiO-66, 56%-56%=0

for UiO-67, 61%-60%=1%

for MIL-101-Cr, 4%-4%=0

for mesoporous silica, 94%-94%=0

for Lipo, 36%-3%=33%

for Neofect, 39%-15%=24%

The above results are the releasing efficiency= the amount of ssDNA released from the vetors/the whole amount of ssDNA. The above results are in consistent with the results calculated by fluorescence quench and recovery.

Analysis of stability of ssDNA in 10% FBS solution by 20% denaturing polyacrylamide gels electrophoresis

The ssDNA loaded materials were mixed with 10% FBS (volume percentage). After incubation for 24 h at 37 °C, 50 μL was taken from the suspension for each material. Formamide (50 μL) was added into the suspensions. Then the mixtures were heated at 95 °C for 10 min and immediately cooled down to 4 °C. Finally, 6 μL 6x loading buffer solution was added to 100 μL of the mixture and mixed up. 6 μL of the above mixture was loaded to the 20% denaturing polyacrylamide gel, and electrophoresis was run at 300 V for 4 hours. Then the polyacrylamide gel was scanned with a Pharos FX Molecular imager (Bio-Rad, USA) operated in the fluorescence mode and FAM labeled ssDNA content was quantified by BIO-RAD quantity software. Naked FAM labeled ssDNA incubation with 10% FBS was used as control. Neofect, Lipo and Ni-IRMOF-74-II to V provide excellent protection against the degradation. In contrast, SBA-15 showed a poor performance because the large pore size allows for the entrance of degradation enzyme (Supplementary Figure 89).

$$survival\ efficiency\ (\%) = \frac{FI_{suvival}}{FI_{control}} \times 100\% \qquad (9)$$

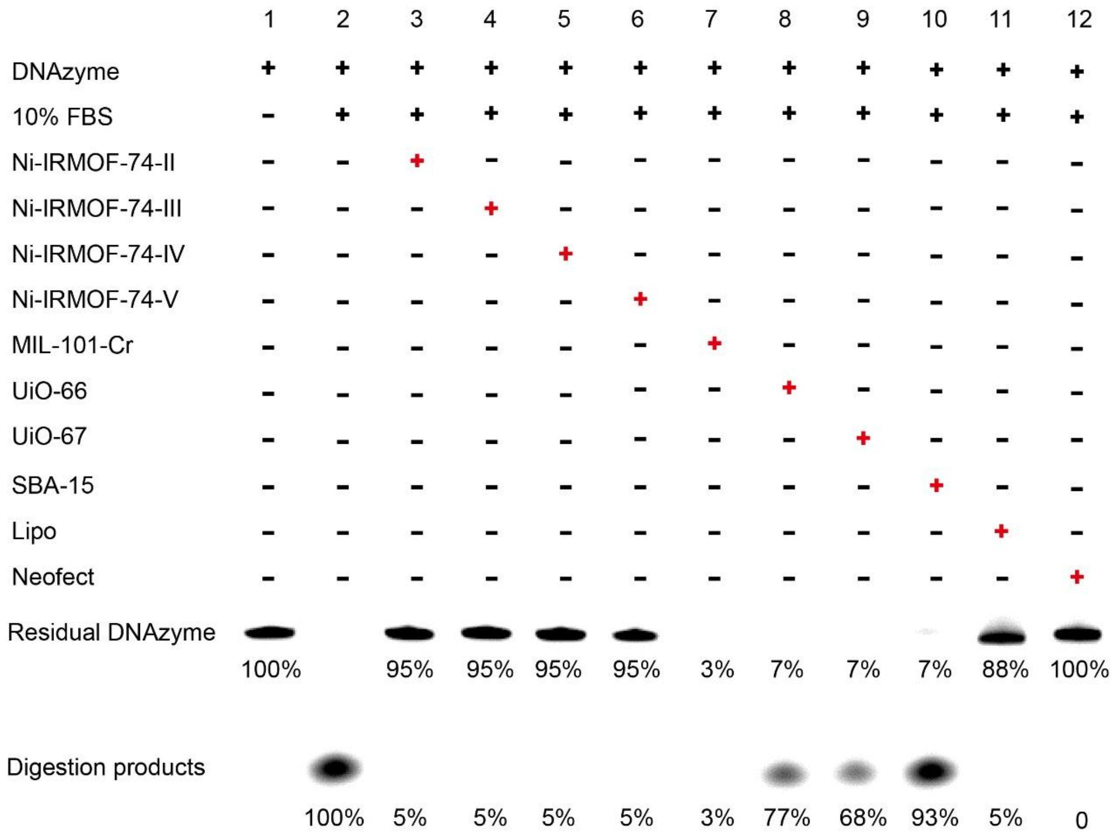

Supplementary Figure 89 Serum stability of ssDNA loaded in different vectors in 10% FBS solution.

Analysis of DNAzyme catalytic efficiency by 20% denaturing PAGE

10-23 DNAzyme is a ssDNA composed of a cation-dependent catalytic core of 15 nucleotides, which is flanked by a substrate binding arm that specifically bind with their complementary sequence and cleave the target RNA. We use the substrate DNA sequence according to the literature[35], where two ribonucleotides present in the middle of the sequence. Mg^{2+} ion could trigger 10-23 DNAzyme to completely cleave the substrate. The substrate DNA (0.25 μM) and DNAzyme (1.25 μM) were incubated with predetermined Mg^{2+} concentration in 10 μL 50 mM Tris-HCl buffer (pH = 8.0) at 37 °C for 5 h. Later, the solution was quenched by adding 10 μL formamide. Then the mixtures were heated at 95 °C for 10 min and immediately cooled down to 4 °C. Finally, 2 μL 6x loading buffer solution was added to 20 μL of the above mixture. The 5 μL of the prepared solution was loaded to the 20% denaturing polyacrylamide gel, and electrophoresis was run at 300 V for 4 hours. Pharos FX Molecular imager (Bio-Rad, USA) was used to analyze the gel after GelRed staining for 30 min. As showed in Supplementary Figure 90, DNAzyme can efficiently digest their substrate under the cofactors of Mg^{2+} (Supplementary Figure 90).

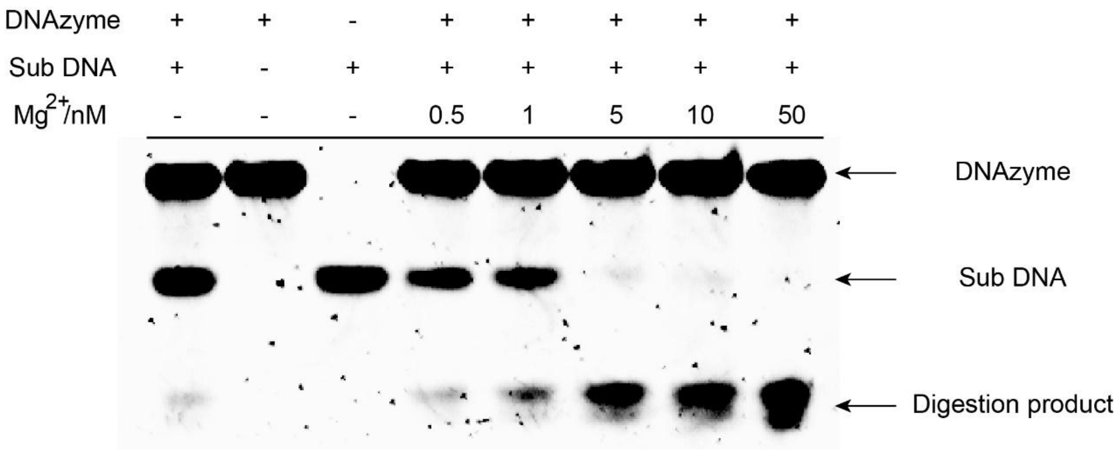

Supplementary Figure 90 Catalytic efficiency of DNAzyme analyzed by 20% PAGE (Mg^{2+} as the cofactors).

Supplementary Method 6.

Investigation of Interactions between ssDNA and MOF Pores

X-ray photoelectron spectroscopy (XPS) was performed on a Thermo Fisher ESCALAB 250Xi using a monochromatic Al Kα X-ray source. All binding energies were referenced to the C 1s peak (284.8 eV) arising from adventitious carbon[16]. The energy scales are aligned by using the Fermi level of the XPS instrument (4.57 eV versus absolute vacuum value) (Supplementary Figure 91-95)[17]. In the case of Ni-IRMOF-74-II, no obvious shift was found in the Ni peaks before and after the loading of ssDNA into MOF pores (Supplementary Figure 91). This indicated no significant interaction exists between metal site and ssDNA in Ni-IRMOF-74-II. In contrast, the Ni peaks in the XPS spectrum of Ni-MOF-74, peaks of Zr in UiO series and those of Cr in MIL-101 shift after the addition of ssDNA. Thus there are interactions between ssDNA and metals in these MOFs. Specifically, the binding energy of 2d3/2 orbital of Ni shifted by 0.2 eV (Supplementary Figure 91). The binding energy of $2d_{5/2}$ orbital of Zr in UiO-66 shifted by 0.5 eV (Supplementary Figure 94). The binding energy of $2p_{3/2}$ orbital of Cr in MIL-101 shifted by 0.2 eV, showing a relatively weaker interaction between Cr and ssDNA (Supplementary Figure 93). On the other hand, no shift for the $2p_{3/2}$ in Ni of Ni-IRMOF-74-II before and after loading ssDNA proved there is little interaction between ssDNA and metal site (Ni) (Supplementary Figure 92).

As illustrated by the XPS spectra of Ni-MOF-74 and Ni-IRMOF-74-II, the binding energy of Ni for the ssDNA absorption in Ni-MOF-74 changed by 0.2 eV, revealing the existence of surface binding due to the coordination effect with the metal site on the surface of Ni-MOF-74. However, there is no shift for the Ni in the XPS spectra of Ni-IRMOF-74-II after the loading of ssDNA, indicating little change in chemical environment which could confirm the ssDNA absorption in MOF pores rather than the surface. These different absorption behaviors could be explained by the difference in zeta potential (Supplementary Table 14) for Ni-MOF-74 (-0.4 mV) and Ni-IRMOF-74-II (-9.4 mV). The electrostatic repulsion on the surface between ssDNA (-13.8 mV) and Ni-IRMOF-74-II are much stronger than that between ssDNA and Ni-MOF-74, thus leading to little absorption on the surface of Ni-IRMOF-74-II. The experiments above have already demonstrated the accuracy for quantification considering the interference of surface binding.

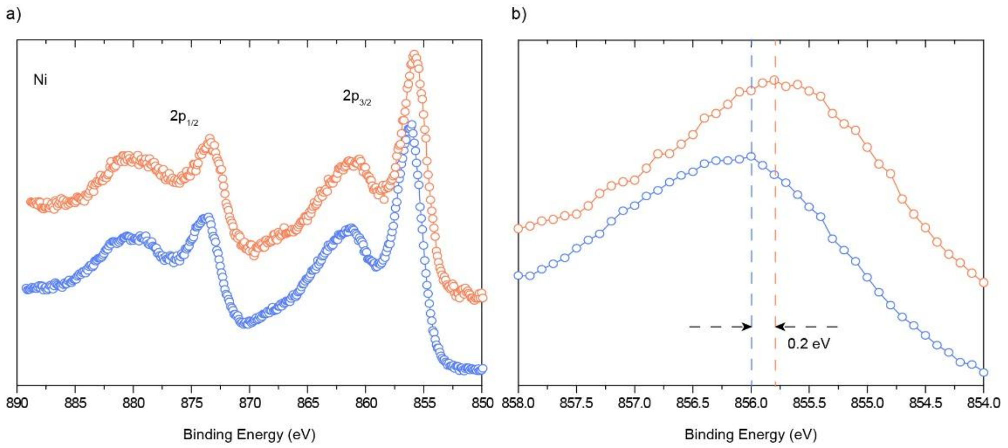

Supplementary Figure 91 XPS measurements to reveal the interaction between ssDNA and Ni in Ni-MOF-74. XPS spectra of Ni-MOF-74 (light blue) and ssDNA loaded Ni-MOF-74 (orange) are overlaid with broad (a) and zoomed in (b) energy width.

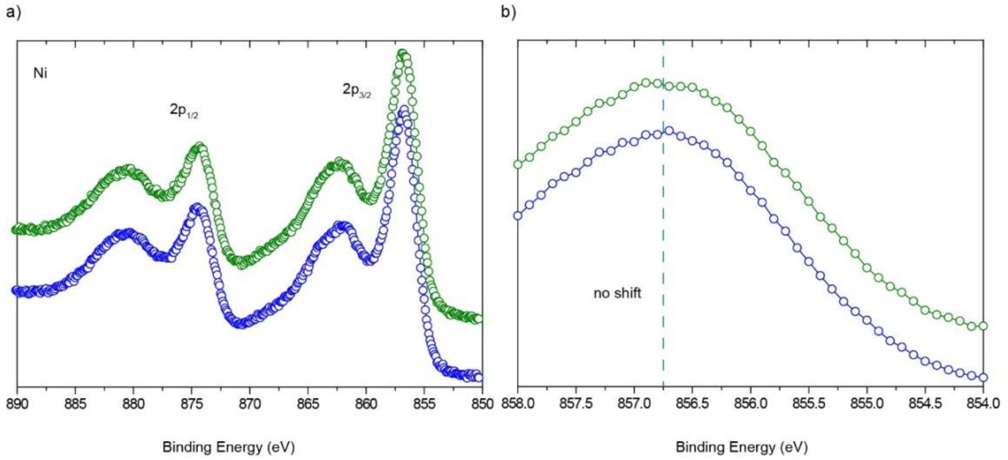

Supplementary Figure 92 XPS measurements to reveal the interaction between ssDNA and Ni in Ni-IRMOF-74-II. XPS spectra of Ni-IRMOF-74-II (blue) and ssDNA loaded Ni-IRMOF-74-II (green) are overlaid with broad (a) and zoomed in (b) energy width.

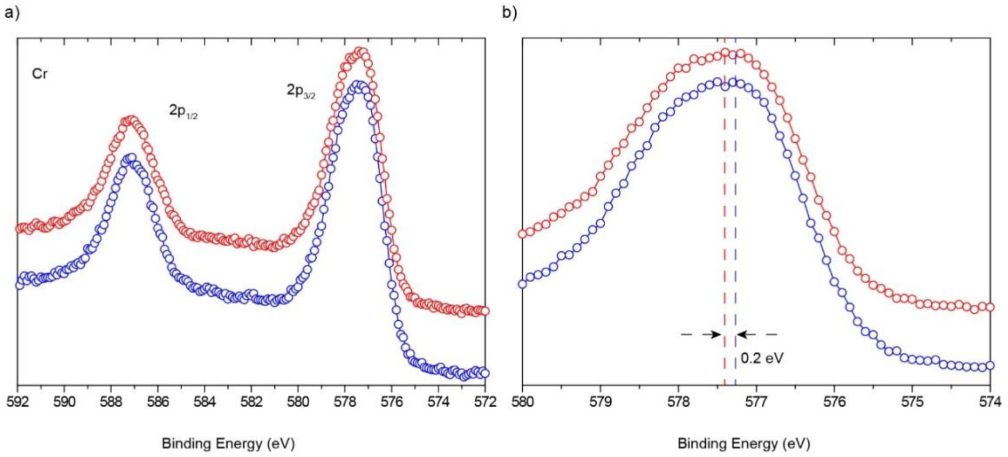

Supplementary Figure 93 XPS measurements to reveal the interaction between ssDNA and Cr in MIL-101. XPS spectra of MIL-101-Cr (blue) and ssDNA loaded MIL-101-Cr (red) are overlaid with broad (a) and zoomed in (b) energy width.

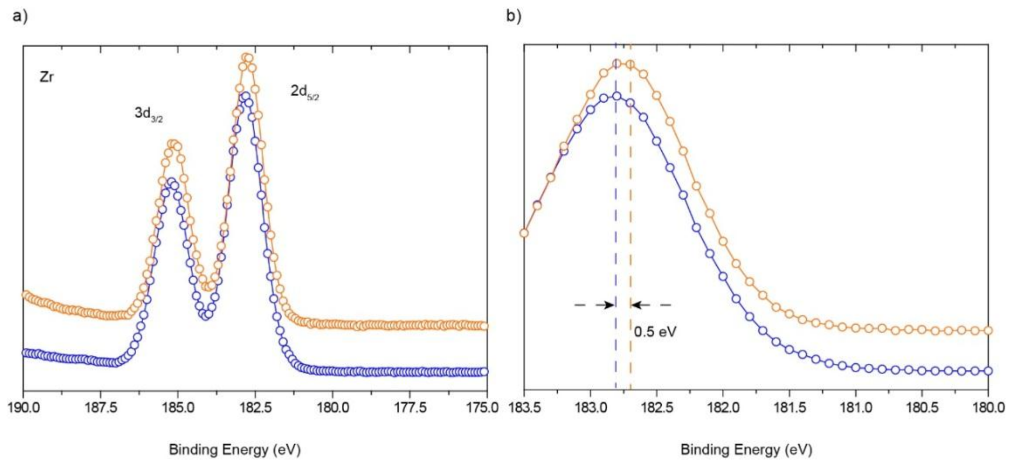

Supplementary Figure 94 XPS measurements to reveal the interaction between ssDNA and Zr in UiO-66. XPS spectra of UiO-66 (blue) and ssDNA loaded UiO-66 (orange) are overlaid with broad (a) and zoomed in (b) energy width.

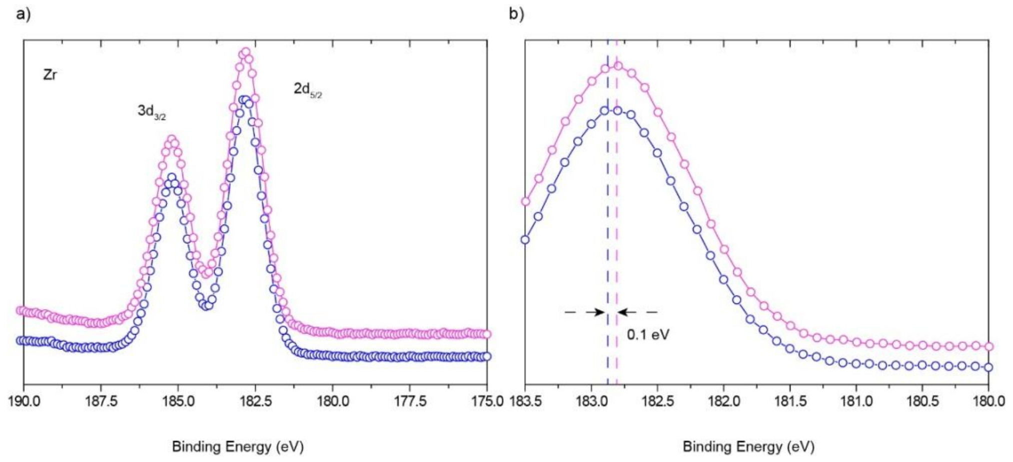

Supplementary Figure 95 XPS measurements to reveal the interaction between ssDNA and Zr in UiO-67. XPS spectra of UiO-67 (blue) and ssDNA loaded UiO-67 (purple) are overlaid with broad (a) and zoomed in (b) energy width.

X-ray adsorption spectra (XAS) of ssDNA loaded Ni-IRMOF-74-II

In order to confirm the interaction between ssDNA molecules and SBU metals in MOFs and further study the coordination environment of metals in MOFs after loading with ssDNA, X-ray absorption spectroscopy (XAS) measurements were performed. Pristine Ni-IRMOF-74-II samples were divided into two parts. Both of them were treated in aqueous solution. One was loaded with excess ssDNA, while the other was used as control. Then both samples were washed with double-distilled water and dried under vacuum with centrifugation for 24 hours (Supplementary Figure 96).

METHODS:

XAS measurements were performed on the bending magnet beam line 14W1 and 15U at Shanghai Synchrotron Radiation Facility (SSRF). Samples were tableted as a 1

cm^2 square disk. Measurements were performed in transmission detection mode in air at room temperature.

XANES and EXAFS analysis was performed using the Athena/Artemis/Hephaestus software package which makes use of IFEFFIT[18]. Athena was used for XAS data processing including conversion of raw data to μ(E) spectra, background subtraction, Fourier transforming and plotting. Artemis was used to analyze EXAFS data, including the range of the Fourier transform from *k*-space and the fitting range in *R*-space. XANES is a sensitive technology to determine the oxidation state of metals in which adsorption edge show significant shifts (binding energy shifts) with oxidation state. K-edge for Ni in Ni-IRMOF-74 and Ni-IRMOF-74-ssDNA were shown in Figure 3.

Adsorption edge of Ni in Ni-IRMOF-74-II is similar to that of ssDNA loaded Ni-IRMOF-74-II samples, suggesting that the chemical environment of Ni was not influenced by ssDNA inclusion into the MOF pores.

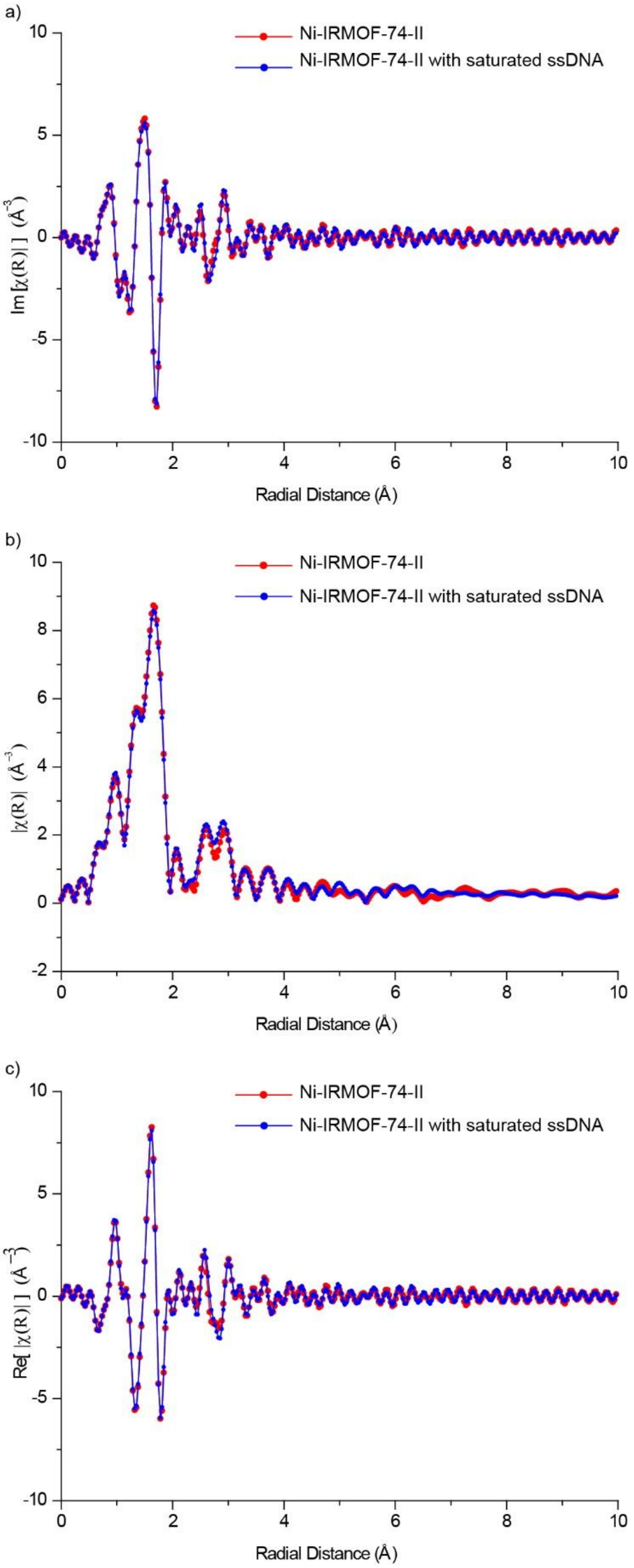

Supplementary Figure 96 a) Imaginary part of R-space, b) Real part of R-space, c) Magnitude of R-space.

Zeta potential analysis

Zeta potential was tested in aqueous solution to determine the charges around the particles of MOFs and ssDNA when dispersed in water[19] (Supplementary Table 14, Supplementary Figure 97). Zeta potential was measured by laser doppler micro-electrophoresis (Zetasizer, Nano-ZS, Malvern, UK). Both of ssDNA and Ni-IRMOF-74-II are negative suggesting that the interaction between them is not electrostatic force (Supplementary Figure 97). In contrast, the strong interaction between ssDNA and MIL-101-Cr may originate from the electrostatic interaction due to their opposite charges (Supplementary Figure 97). When the pH of the aqueous solution changes or the particle size of Ni-IRMOF-74 series varies, the Zeta potential of the materials remains negative (Supplementary Figure 97). All measurements were reproduced for three times.

Supplementary Table 14 Summary of Zeta potential of different materials.

Sample	Zeta potential (mV)	Error range (±mV)
ssDNA	-13.8	1.8
Ni-MOF-74	-0.4	0.2
Ni-IRMOF-74-II	-9.4	0.5
Ni-IRMOF-74-III	-7.6	0.4
Ni-IRMOF-74-IV	-2.9	0.3
Ni-IRMOF-74-V	-3.5	1.2
MIL-101	45.9	0.9
UiO-66	50.5	0.9
UiO-67	7.1	0.5

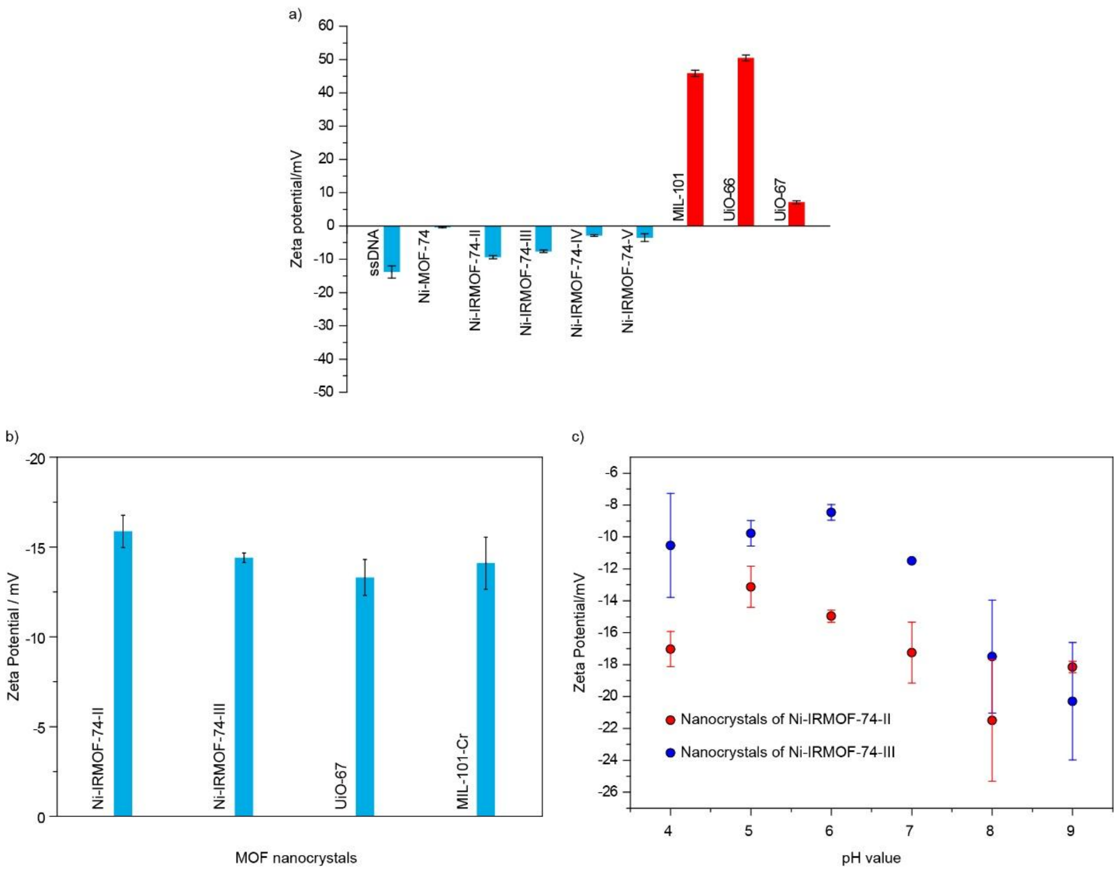

Supplementary Figure 97 Zeta potential in various solution a) Zeta potential distribution of different materials in aqueous solution, b) Zeta potential distribution of different MOF nanocrystals in cell culture medium (DMEM) (n = 3 technical replicates; bars represent mean ± s.d.). c) Zeta potential distribution of nanocrystals Ni-IRMOF-74-II and -III in aqueous solution with pH varying from 4 to 9 (n = 3 technical replicates; bars represent mean ± s.d.).

Supplementary Method 7.

ssDNA Transfection Efficiency Revealed by Confocal Laser Scanning Microscopy and Flow Cytometry

Detection of the intracellular delivery by confocal laser scanning microscopy (CLSM)

The fluorescence in the cells was detected by using a laser scanning confocal microscope (Leica TCS SP2, Germany). MCF-7 cells were incubated with FAM labeled DNAzyme (FAM-DNAzyme, 100 nM) loaded nano-MOF and naked FAM-DNAzyme (100 nM) for 3 h at 37 ℃, respectively. The sequence of FAM-DNAzyme is FAM-CCGCGGCCAGGCTAGCTACAACGACCTGGACGA. The cells were washed with PBS three times and continued to develop 3 h with fresh medium at 37 ℃. Then, they were fixed with 4% paraformaldehyde for 20 min at 4 ℃, and sequentially stained with DAPI (10 μg/mL) for 20 min at room temperature before observation by CLSM. As shown in Figure 4c, FAM-DNAzyme can be successfully delivered into cells by nano-MOF, but naked FAM-DNAzyme cannot be introduced into the cells.

In order to visualize the co-localization of internalized nano-MOF loaded with DNAzyme and endosomal/lysosomal compartments, MCF-7 cells were incubated with FAM-DNAzyme（100 nM) in nano-MOF for 3 h at 37 ℃. The cells were washed with PBS three times and continued to develop 3 h with fresh medium at 37 ℃. Then, they were fixed with 4% paraformaldehyde for 20 min at 4 ℃, and sequentially stained with Lysotracker Red (100 nM) for 2 h at room temperature and then DAPI (10 μg/mL) for 20 min at room temperature before observation by CLSM (Supplementary Figure 98). This endosome/FAM-DNAzyme colocalization showed that FAM-DNAzyme was able to escape from the endo/lysosome entrapment, as demonstrated by the separation of red (lysosome stained by Lysotracker Red) and green (FAM-DNAzyme) fluorescence in the cytoplasm (Supplementary Figure 98). These results show that the DNAzyme can be taken up into the cytoplasm through phagocytosis by using the nano-MOF as vector.

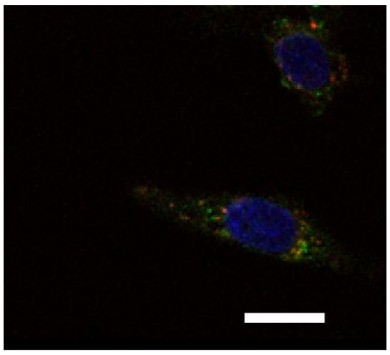

Supplementary Figure 98 CLSM image for colocalization studies, endosome/lysosome and nuclei were stained with Lysotracker Red (red) and DAPI (blue), respectively. DNAzyme was labeled with green fluorescence. scale bar: 20 μm.

Flow cytometry

Method:

Preparation of ssDNA loaded vector mateials

In this experiment, the uptake of ssDNA by Lipo and cationic polymer (Neofect) were described in the method part of main text. The method of uptake FAM-ssDNA by Lipo was shown as the following. 1 μL FAM-ssDNA (100 μM) was diluted to 50 μL culture medium, and Lipo regent (3.6 μL) was diluted to 50 μL culture medium. Finally, the above two components were mixed up and incubated at room temperature for 20 min.

The method of uptake FAM-ssDNA by cationic polymer (Neofect) was shown as the following. 1 μL FAM-ssDNA (100 μM) was diluted to 50 μL culture medium and then 1.5 μL Neofect reagent was added. The solution was mixed up and incubated at room temperature for 20 min.

For the MOF vectors in this study, Ni-IRMOF-74-II, Ni-IRMOF-74-III, UiO-67, and MIL-101-Cr were prepared as the following. 1 μL FAM-ssDNA (100 μM) was incubated with saturated vectors in 100 μL aqueous solution under 1000 rpm vibration at 37 °C for 1 h. Then the solution was centrifuged under 12000 rpm for 15 min and the supernatant was decanted before collecting the precipitation.

Analysis through flow cytometry

Double channel (FITC channel and PI channel) analysis by the Flow cytometry were used in this study to evaluate and compare the cell transfection efficiency and cell cytotoxicity of different MOF vectors as well as other commercial vectors. Propidium iodide (PI) uptake versus exclusion was used to discriminate dead cells from live cells. Plasma membranes of the dead cells become permeable regardless of the mechanism of death, while live cells are not permeable due to the protection by intact membranes. Thus, PI result reflected the cell cytotoxicity of these materials. FITC channel detect the fluorescence of the FAM-ssDNA, and represent the amount of the FAM-ssDNA that was delivered into the cells. Protocol of analysis through flow cytometry was mentioned in the method part in main text.

Comparison of transfection efficiency in MCF-7 cells revealed by flow cytometry

As shown in Supplementary Figure 99a, MCF-7 cells were treated with the fixed amount of FAM-ssDNA (400 nM) carried by vectors (Ni-IRMOF-74-II, Lipo and Neofect) in saturation for 20h. The cells treated with Lipo showed the highest cytotoxicity (~90% dead cells) than the other two vectors (~7% dead cells for Ni-IRMOF-74-II, ~23% dead cells for Neofect). In addition, Ni-IRMOF-74-II showed the

highest transfection efficiency (~32% successfully transfected cells without damage) than the other two vectors (~13% for Lipo and ~14% for Neofect, respectively). Thus, Ni-IRMOF-74-II exhibits the best performance with the lowest cytotoxicity and highest transfection efficiency to delivery 400 nM ssDNA into cells. In another comparison as shown in Supplementary Figure 99b, MCF-7 cells were treated with FAM-ssDNA loaded by vectors of the fixed amount (46 μg) in saturation for 20 h. Ni-IRMOF-74-II showed the highest transfection efficiency (~32% successfully transfected cells without damage). However, in this condition, Lipo and Neofect didn't demonstrate any the cell transfection capability.

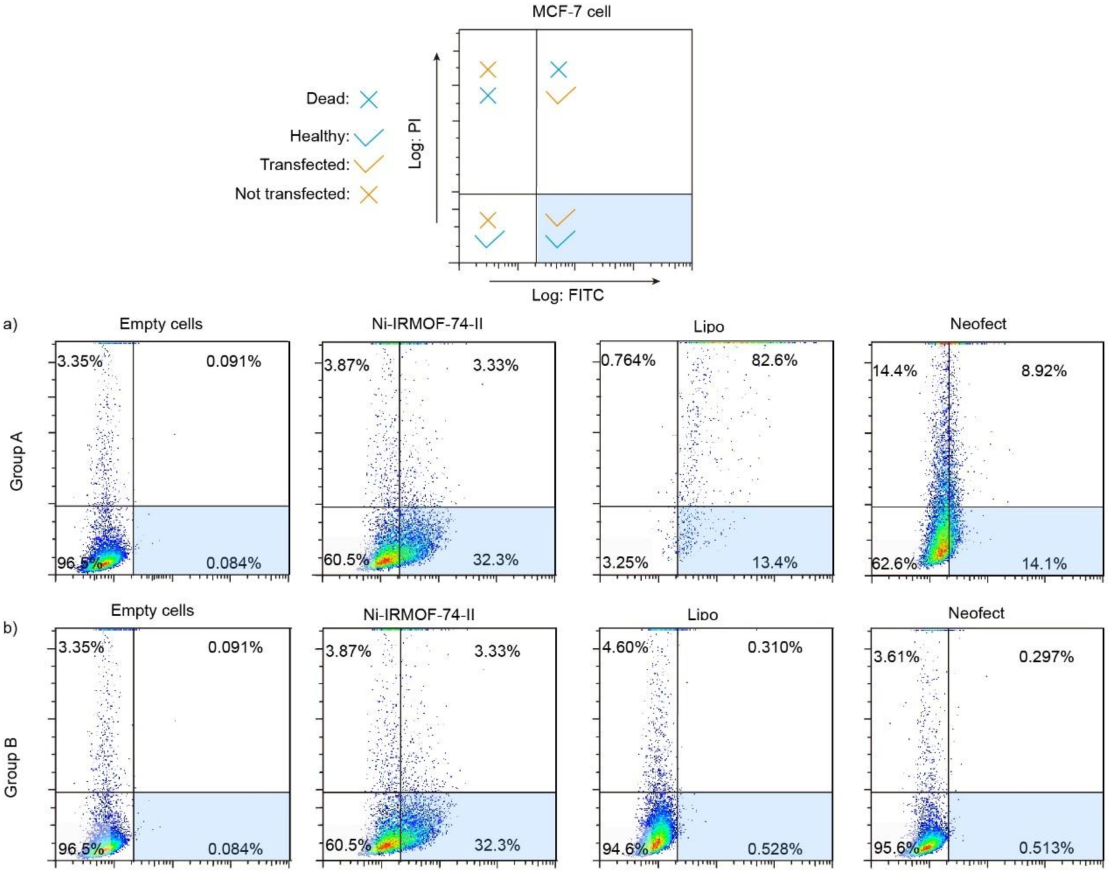

Supplementary Figure 99 Flow cytometry analysis by PI channel and FITC channel of MCF-7 cells recorded in treatments using various vectors. a) Group A, MCF-7 cells treated with saturated vectors carrying fixed amount of FAM-ssDNA (400 nM) for 20h, respectively. b) Group B, MCF-7 cells treated with fixed amount of vectors (46 μg) saturated with FAM-ssDNA for 20 h.

Comparison in loading amount of ssDNA in different vectors

MCF-7 cells were treated with vectors (Ni-IRMOF-74-II, Lipo and Neofect) saturated with various concentrations of FAM-ssDNA for 20 h, in which the amount of vector increases as the total loading of FAM-ssDNA increases. The concentration FAM-ssDNA loaded in Ni-IRMOF-74-II vector varies from 0 nM to 800 nM. Cells treated these samples showed that about 37% cells were successfully transfected without damage and only 8% cells were dead when the FAM-ssDNA concentration is

as much as 800 nM (Supplementary Figure 100a). In contrast, cells treated with Neofect as vector carrying various concentration FAM-ssDNA (from 0 nM to 400 nM), showed only 10% successful cell transfection without damage, while 54% cells were dead (Supplementary Figure 100b). This was observed at an even lower concentration of FAM-ssDNA (400 nM) in comparison to the concentration used in Ni-IRMOF-74. The least loading was found when lipo was used as vector. Cells were treated with FAM-ssDNA loaded Lipo with the concentration of 0 nM to 200 nM (Supplementary Figure 100c). Only 8% cells were successfully transfected without damage and 70% cells were dead when the FAM-ssDNA concentration reached 200 nM. This comparison illustrated the superior loading performance and low cell cytotoxicity featured in Ni-IRMOF-74-II as vector (Supplementary Figure 100). Thus, this MOF shows potential as high efficiency transfection reagent.

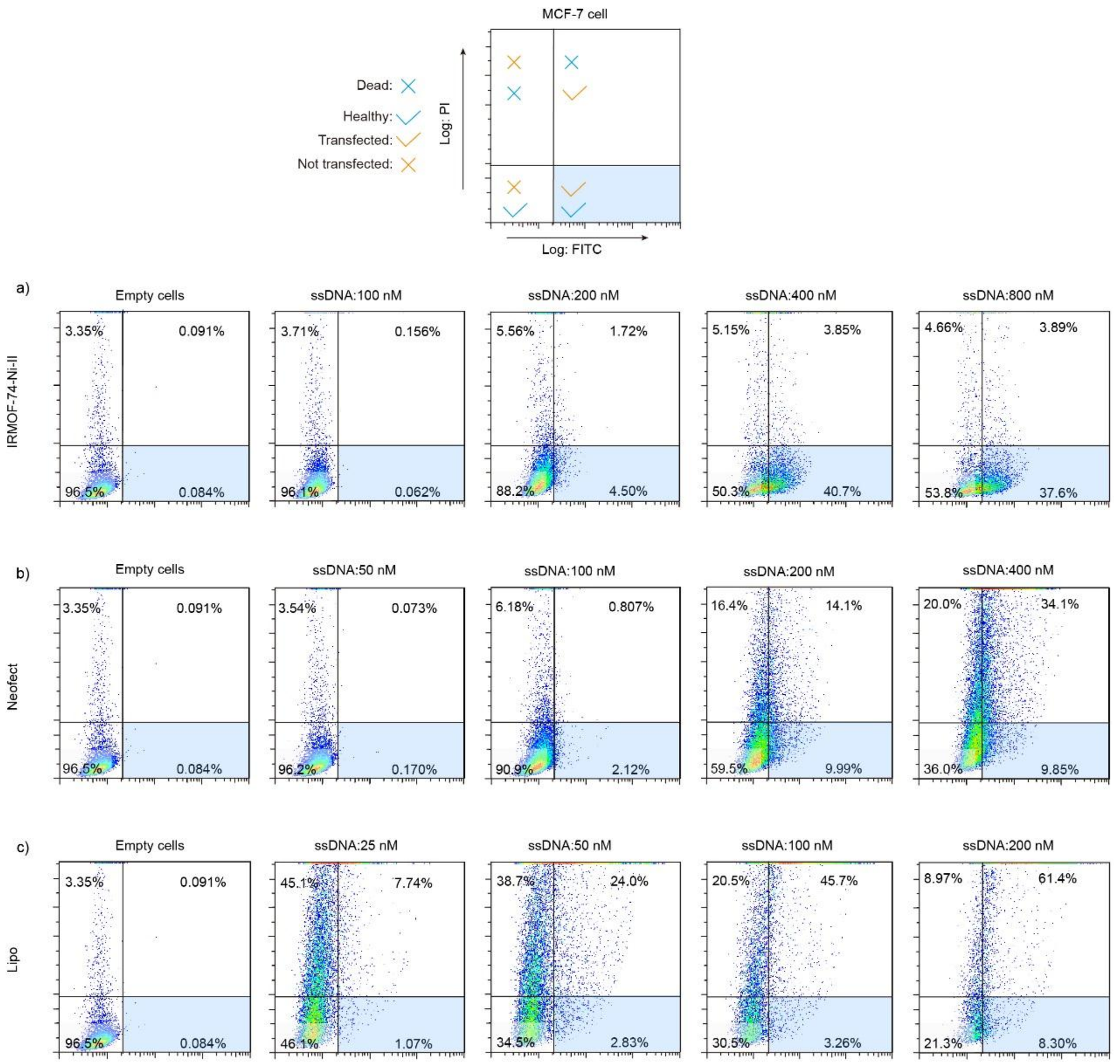

Supplementary Figure 100 Flow cytometry analysis by PI channel and FITC channel of MCF-7 cells treated with different concentration of FAM-ssDNA saturated in various vectors.

Release efficiency of different ssDNA loaded into the nano-Ni-IRMOF-74-II (nano-MOF) in MCF-7cells

The cultured MCF-7 cells were incubated with fixed amount of nano-MOF loaded with FAM-DNAzyme (DNAzyme), nano-MOF loaded with scrambled ssDNA (SC-DNA) with FAM label, pure nano-MOF sample, and naked FAM-DNAzyme, in parallel experiments. Flow cytometry was applied to analyze the fluorescence of these cells. As shown in Supplementary Figure 101, the fluorescence of cells incubated with FAM-DNAzyme loaded nano-MOF was almost 16 times of that with the untreated cells, and 2.4 times of that with SC-DNA loaded nano-MOFs. SC-DNA is a scrambled sequence DNA, with little complementary sequences in cells. This result confirms that DNAzyme was released in a controlled manner from nano-MOF in the presence of its targeting mRNA.

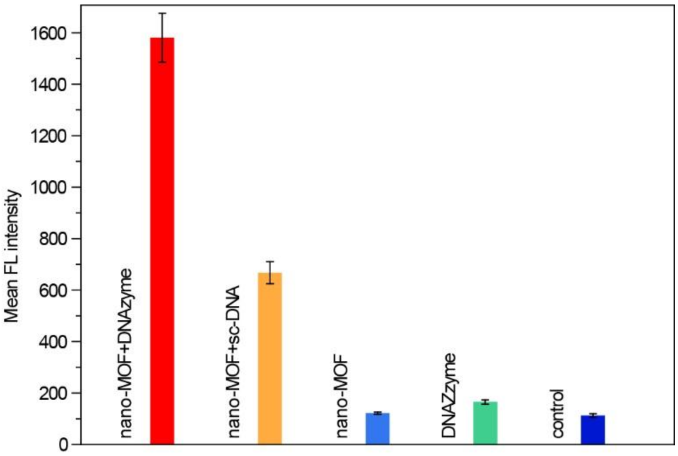

Supplementary Figure 101 Flow cytometry analysis of the fluorescence of MCF-7 cells treated with DNAzyme loaded nano-MOF, SC-DNA loaded nano-MOF, pure nano-MOF, and pure DNAzyme with the control experiment of cells without treatment of any fluorescent dye (n = 3 technical replicates; bars represent mean ± s.d.).

Comparison of the transfection efficiency using various vectors in RAW264.7 and THP-1 cell lines

Transfect efficiencies in RAW264.7 and THP-1 cells were measured by Flow cytometry. RAW264.7 and THP-1 cells were treated with saturated vectors (Ni-IRMOF-74-II and -III, Lipo and Neofect) loaded with 400 nM FAM-ssDNA for 3 h and 5 h, respectively. ssDNA was delivered into the macrophages of RAW264.7 cells lines using various vectors. The FITC signal increased dramatically in the RAW264.7 cells treated with ssDNA loaded in Ni-IRMOF-74-II and -III, exhibiting transfection efficiencies of 90 % and close to 100 %, respectively (Supplementary Figure 102b). However, UiO-67 and MIL-101-Cr did not work in an effective way and their transfection efficiencies were quite low, 13 % and 16 %, respectively (Supplementary Figure 102b). Commercial transfection agents (Lipo and Neofect) were also used as comparison. Both carriers provide about 50 %

transfection efficiency, however, more than 30 % of cells died in the successful transfected portion in Lipo treatment, while 25 % cells died in Neofect treatment. In contrast, there is less than 20% cells died among the 90 % successfully transfected cells using Ni-IRMOF-74-II as vectors. Ni-IRMOF-74-III showed even better performance with less than 10 % cell died among almost 100% successfully transfected cells (Supplementary Figure 102a). These MOFs were further tested for transfection in a more challenging cell line, human macrophage, THP-1. Lipo and Neofect caused death of a large portion of cells (83% and 36%, respectively) and exhibited limited transfection efficiency, 6% and 11%, respectively. In the case of Ni-IRMOF-74-III, it achieved more than 30% transfection efficiency and only gave negligible damage (4% death) to cells (Supplementary Figure 102c). In addition, UiO-67 and MIL-101-Cr were also used as comparison. Neither of them exhibit sufficient transfection efficiencies (14% and 5%, respectively, Supplementary Figure 102d). These comparisons above clearly demonstrated the potential of Ni-IRMOF-74-II and -III to serve as vectors to provide high transfection efficiency and low cytotoxicity. Their performances are way better than those of the commercial vectors (Lipo and Neofect) in transfection for RAW264.7 cell and THP-1 cells.

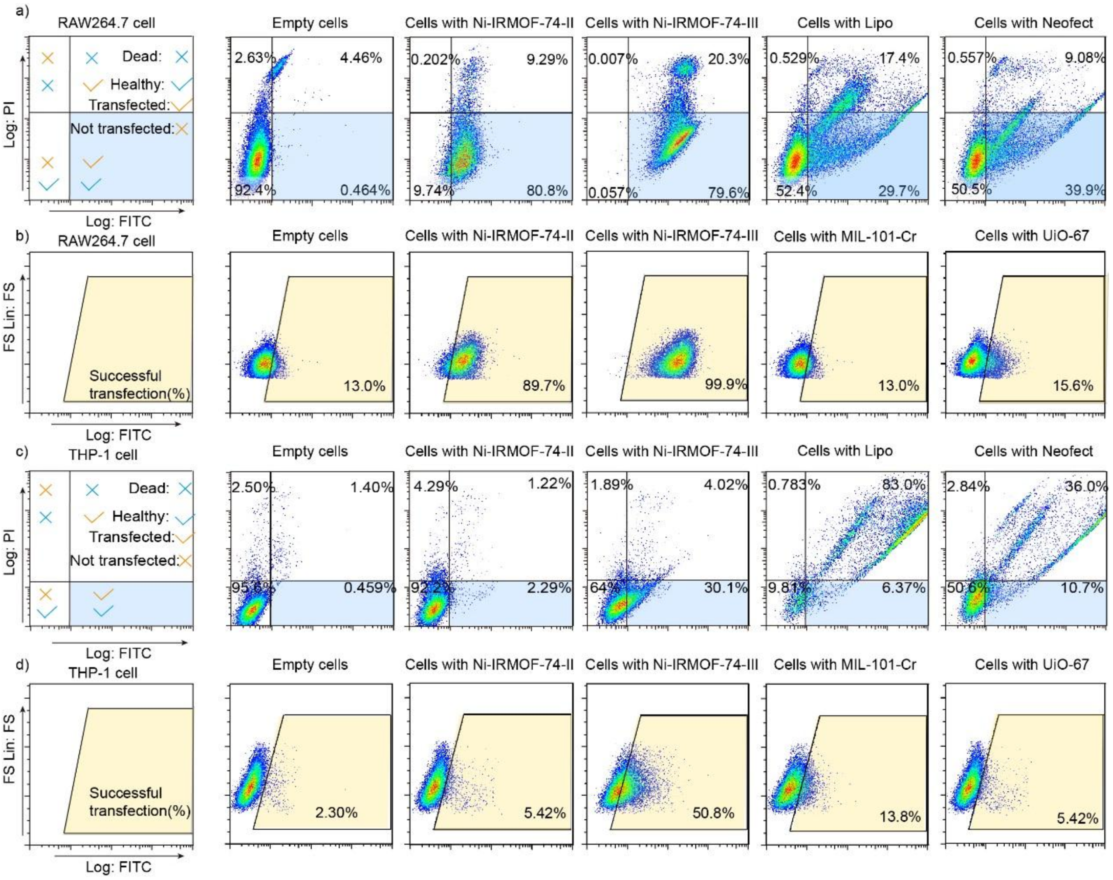

Supplementary Figure 102 Transfect efficiencies in RAW264.7 and THP-1 cells a) Flow cytometry analysis by both FITC channel and PI channel in RAW264.7 cells. b) Flow cytometry analysis by FITC channel in RAW264.7 cells. c) Flow cytometry analysis by both FITC channel

and PI channel in THP-1. d) Flow cytometry analysis by FITC channel in THP-1.

Transfection of CD4+ T cell and B cell

In order to eliminate the phagocytosis of special cell lines and extend the applicability in a wide range of cell types, the primary mouse immune cells (CD4+ T cells and B cells) were tested and the results are shown below (Supplementary Figure 103).

CD4+ T cells were isolated from mouse splenocytes through immunomagnetic negative selection using EasySep™ Mouse CD4+ T Cell Isolation Kit. Untouched B cells were isolated from mouse spleen cell suspensions using the B Cell Isolation Kit, an LS Column, and a MidiMACS™ Separator.

The primary mouse Immune cells (CD4+ T cells and B cells) were treated with saturated vectors (Ni-IRMOF-74-II and -III, Lipo and Neofect) loaded with 400 nM FAM-ssDNA for 24 h, respectively.

In CD4+T cells, Ni-IRMOF-74-II and -III stand out with high transfection efficiency, 61% and 92% respectively. Both of them are better than the commercial agent, Lipo (58%) and Neofect (50%), which is nearly only a half of Ni-IRMOF-74-III. And Ni-IRMOF-74-II and -III also exhibited high efficiency in B cell, 68.4% and 88.8 % respectively. Ni-IRMOF-74-III is still the best among them, nearly 20% higher than the efficiency of commercial agents. Based on these results, nanocrystals of Ni-IRMOF-74-II and -III have a remarkable potential to be universal across cell types.

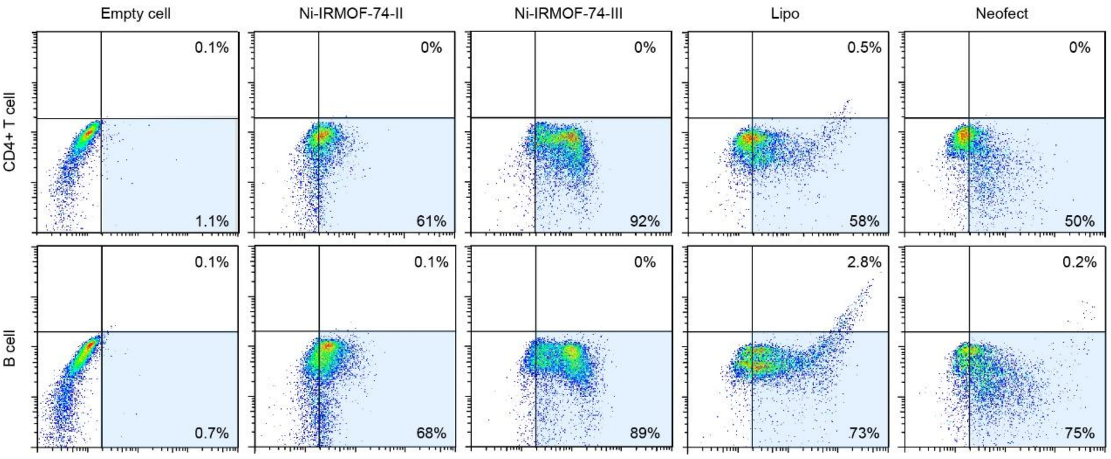

Supplementary Figure 103 Transfection efficiency and toxicity of nanocrystals of Ni-IRMOF-74-II and -III reflected in Flow cytometry in in comparison with Lipo and Neofect in the primary mouse Immune cells (CD4+ T cells and B cells). The primary mouse Immune cells were treated with saturated vectors (Ni-IRMOF-74-II and -III, Lipo and Neofect) loaded with 400 nM FAM-ssDNA for 24 h, respectively.

Supplementary Method 8.

Cytotoxicity Assessment of Ni-IRMOF-74 Series through Cell Proliferation Assays

The cytotoxicity test of nano-MOF in MCF-7 cells by the standard MTT

The cytotoxicity of nano-MOF was evaluated by the standard MTT [3-(4,5-dimethylthiazol-2-yl)-2,5-diphenyltetrazolium bromide] assay. MCF-7 cells were seeded into a 96-well plate with a density of 1×10^4 cells in each well. After regular incubation for 24 h, the DMEM in each well was replaced by a fresh medium containing nano-MOF (concentration varies between 0 and 400 μg mL^{-1}) and incubated at 37 °C for another 48 h. For the MTT assay, the old medium was removed and replaced by a fresh medium containing 5 mg mL^{-1} MTT in each well, which was discarded after 4 h of incubation, followed by adding 200 μL DMSO in each well to dissolve the formazan salt. The plate was gently shaken for 15 min before the absorbance was measured at 492 nm. In this study, nano-MOF of 200 μg/mL concentration showed ~100% cell viability throughout the test (Supplementary Figure 104).

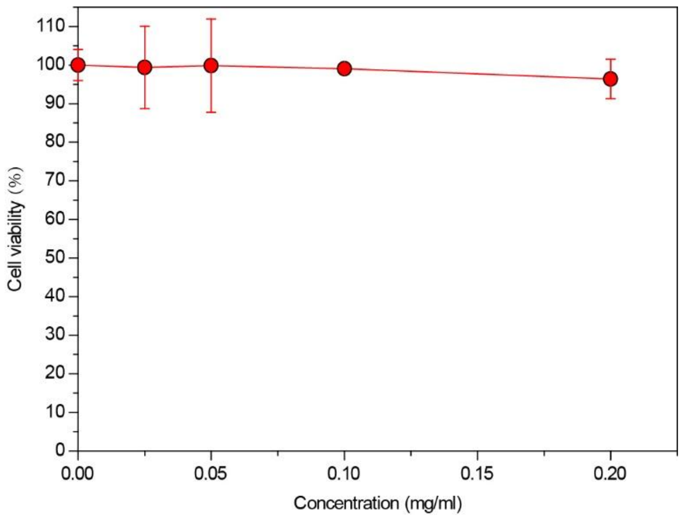

Supplementary Figure 104 Cytotoxicity test of the nano-MOFs in MCF-7 cells. The viabilities of cells incubated with different concentrations of nano-MOF for 48h were measured by MTT assay (n = 5 technical replicates; bars represent mean ± s.d.).

The cytotoxicity test of nano-MOF in MCF-7 cells by the SRB assays

MCF-7 cells were seeded into a 96-well plate (5,000 cells per well), and incubated with 100 μL DMEM containing 10% FBS for 24 h (37 °C, 5% CO_2). After that, the medium was replaced with the material containing medium. The cell viability was measured with the sulforhodamine-B cytotoxicity assay. Specifically, 50 μL of cold 50 % (w/v) trichloroacetic acid (TCA) solution was added to each well. Then, plates were placed at 4 °C for 1 h to anchor the cells. Immediately after the fixation, plates were washed 4 times with PBS and dried under air. After that, 100 μL of 0.4% (w/v) sulforhodamine-B solution (contained 1% acetic acid) was added to each well, and

plates were left at room temperature for 30 min and then quickly washed four times with 1 % acetic acid solution (v/v) to remove the unbound dye. After the drying, 150 μL of 10 mM Tris solution (pH = 10.5) was added to each well and plates were shaken for 20 min to solubilize the dye. The optical density (OD) of each well was predetermined at 540 nm by using a microplate reader (Thermo, Multiskan MK3). The relative cell viability was calculated using the following equation Supplementary Equation 10. $OD_{control}$ was the optical density of wells where cells are without ssDNA loaded vector treatment and OD_{sample} was the optical density of wells where cells are treated with ssDNA loaded vector[20]. Each value was averaged from four independent experiments. As showed in Supplementary Figure 105 and 106, the cytotoxicity of nano-Ni-IRMOF-74-II was significantly lower than commercial transfection regent (Lipo and Neofect).

$$Cell\ viability = OD_{sample}/OD_{control} \times 100\% \qquad (10)$$

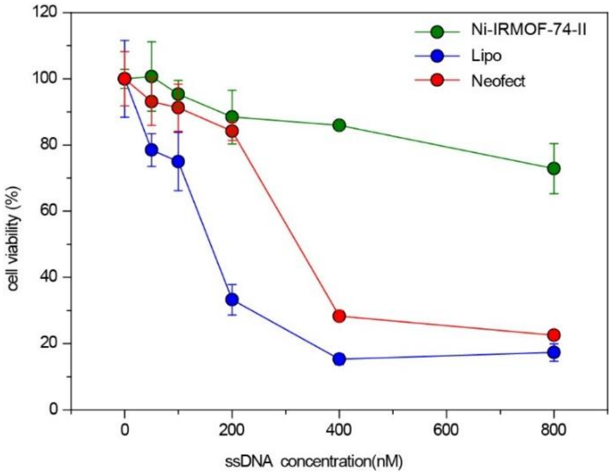

Supplementary Figure 105 Cytotoxicity test of the different concentrations of ssDNA loaded in nano-MOFs, Neofect and Lipo in MCF-7 cells, respectively. The viabilities of cells treated with different concentrations of ssDNA-vector complexes for 48 h were measured by SRB assay ($n = 5$ technical replicates; bars represent mean ± s.d.).

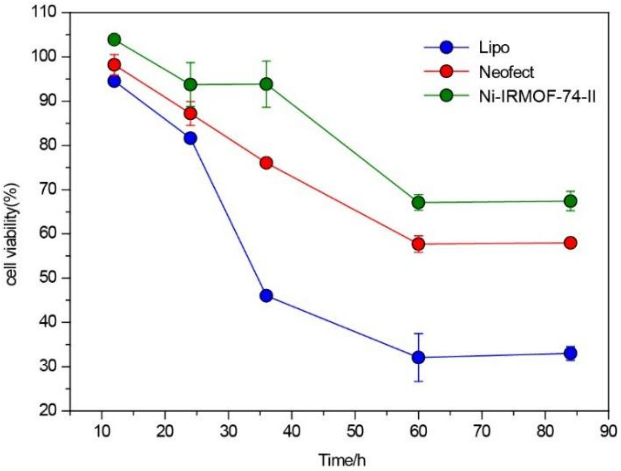

Supplementary Figure 106 Cytotoxicity test of ssDNA (400 nM) loaded in nano-MOFs, Neofect and Lipo in MCF-7 cells at different incubation time (12 h, 24 h, 36 h, 60 h and 84 h, respectively) by SRB assay (n = 5 technical replicates; bars represent mean ± s.d.).

Cytotoxicity test of nano-MOF in RAW264.7 and THP-1 cells by the CCK-8 assays

In each separate experiment, excessive ssDNA was mixed with the vector, and then the mixtures were centrifuged at 12000 rpm for 5 min to remove supernatant. Then, the residual precipitate was re-suspended with DMEM and then introduced to cells (5×10^3 cells per well). CCK-8 assay was applied to assess the cell viability at different incubation time.

The cell viability was evaluated by CCK-8 assay (US Everbright, Inc.). RAW264.7 and THP-1 cells were plated in the 96-well plates (5×10^3 cells per well) and incubated for 24 h. ssDNA loaded vectors were introduced separately to cells with different test concentrations in culture medium. Cells cultured in the medium without adding ssDNA loaded vectors were used as the control. After certain incubation time, the cells were washed with D-Hanks buffer solution. 10 μL of CCK-8 solution was added to each well and incubated for an additional 3 h at 37 °C. The optical density (OD) of each well at 450 nm was recorded on a Microplate Reader (Thermo, Multiskan MK3). The cell viability (% of control) is calculated using equation Supplementary Equation 11, Supplementary Figure 107 and Supplementary Figure 108 illustrated that the cytotoxicity of nano-Ni-IRMOF-74-II and -III was much lower than commercial transfection regent (Lipo and Neofect). Furthermore, cell viability remained more than 90 % in RAW264.7 cells treated with different concentration of nano-Ni-IRMOF-74-II. In the human macrophage, THP-1 cells, cell viability was kept more than 80% when treated with different concentration of ssDNA loaded nano-Ni-IRMOF-74-II.

$$Cell\ viability = (OD_{test} - OD_{blank})/(OD_{control} - OD_{blank}) \quad (11)$$

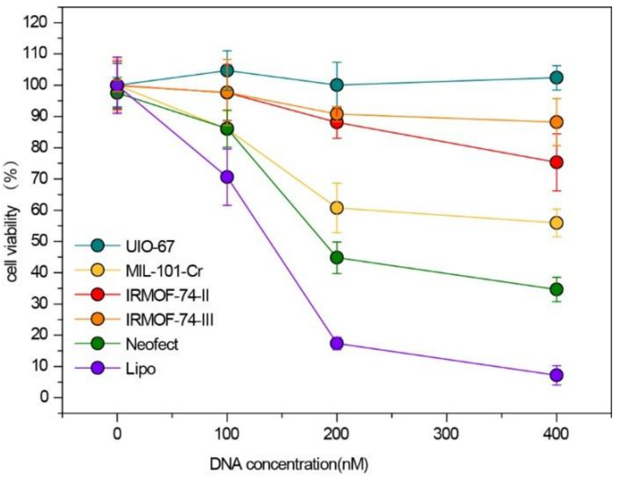

Supplementary Figure 107 The cytotoxicity of vectors with saturated ssDNA in RAW264.7 by the CCK-8 assays. vectors with saturated ssDNA was incubated with RAW264.7 cells for 4 h (n = 5 technical replicates; bars represent mean ± s.d.).

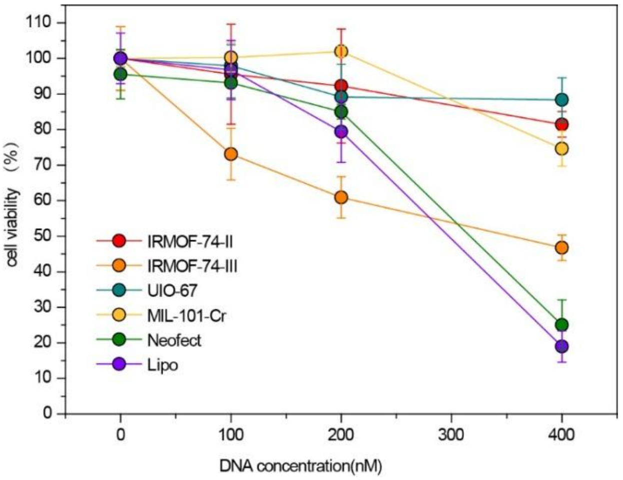

Supplementary Figure 108 The cytotoxicity of vectors with saturated ssDNA in THP-1 by the CCK-8 assays. vectors with saturated ssDNA was incubated with THP-1 cells for 6 h (n = 5 technical replicates; bars represent mean ± s.d.).

Supplementary References

1. Deng, H. *et al.* Large-pore apertures in a series of metal-organic frameworks. *Science* **336**, 1018- 1023 (2012).
2. Zheng. J. *et al.* Pore-engineered metal-organic frameworks with excellent adsorption of water and fluorocarbon refrigerant for cooling applications. *J. Am. Chem. Soc*. **139**, 10601-10604 (2017).
3. Yang. J. *et al.* Tuning the kinetic water stability and adsorption interactions of Mg-MOF-74 by partial substitution with Co or Ni. *Ind. Eng. Chem. Res.* **54**, 12408-12414 (2015).
4. Howe. J. D. *et al.* Understanding structure, metal distribution, and water adsorption in mixed-metal MOF-74. *J. Phys. Chem. C.* **121**, 627-635 (2017).
5. Matzger. A. J. *et al.* Dramatic tuning of carbon dioxide uptake via metal substitution in a coordination polymer with cylinder pores. *J. Am. Chem. Soc*. **130**, 10870-10871 (2008).
6. G. Ferey. *et al.* A chromium terephthalate-based solid with unusually large pore volumes and surface area. *Science* **309**, 2040-2042 (2005).
7. Schaate. A. *et al.* Modulated synthesis of Zr-based metal-organic frameworks: from nano to single crystals. *Chem. Eur. J.* **17**, 6643-6651 (2011).
8. Zhao. D. *et al.* Triblock copolymer syntheses of mesoporous silica with periodic 50-300 angstrom pores. *Science* **279**, 548-552 (1997).
9. Bae, T. & Long. J. R. CO_2/N_2 separation with mixed-matrix membranes containing Mg_2(dobdc) nanocrystals. *Energ. Environ. Sci*. **6**, 3565-3569 (2013).
10. Snurr, R Q. *et al.* Applicability of the BET method for determing surface areas of microporous metal-organic frameworks. *J. Am. Chem. Soc.* **129**, 8552-8556 (2007).
11. Kask, P., Palo, K., Ullmann, D. & Gall, K. Fluorescence-intensity distribution analysis and its application in biomolecular detection technology. *Proc. Natl. Acad. Sci. USA* **96**, 13756-13761 (1999).
12. Yang, S. P. *et al*. Platforms formed from a three-dimensional Cu-based zwitterionic metal-organic framework and probe ss-DNA: selective fluorescent biosensors for human immunodeficiency virus 1 ds-DNA and Sudan virus RNA sequences. *Anal. Chem.* **87,** 12206-12214 (2015).
13. Jinhua, L. *et al*. Combination of *π-π* stacking and electrostatic repulsion between carboxylic carbon nanoparticles and fluorescent oligonucleotides for rapid and sensitive detection of thrombin. *Chem. Commun.* **47**, 11321-11323 (2011).
14. Duan, X. *et al*. Physicochemical characteristics of nanoparticles affect circulation, biodistribution, cellular internalization, and trafficking. *Small* **9**, 1521-1532 (2013).
15. Tamames-Tabar, C. *et al.* Cytotoxicity of nanoscaled metal-organic frameworks. *J. Mater. Chem. B* **2**, 262-271 (2014).

16. Xu H. Q. *et al*. Visible-light photoreduction of CO_2 in a metal-organic framework: boosting electron-hole separation via electron trap states. *J. Am. Chem. Soc.* **137**, 13440-13443 (2015).

17. Li, L. *et al*. Sub-10 nm rutile titanium dioxide nanoparticles for efficient visible-light-driven photocatalytic hydrogen production. *Nat. Commun.* Article 5881, DOI: 10.1038/ncomms6881 (2015).

18. Strawn, D. G *et al.* The use of XAFS to distinguish between inner- and outer-sphere lead adsorption complexes on montmorillonite. *J. Colloid. Interf. Sci***.** **216**, 257-269 (1999).

19. Thielbeer, F., Donaldson, K. & Bradley, M. Zeta potential mediated reaction monitoring on nano and microparticles. *Bioconjugate Chem*. **22**, 144-150 (2011).

20. Vichai, V. & Kirtikara, K. Sulforhodamine B colorimetric assay for cytotoxicity screening. *Nat. Protoc.* **1**, 1112-1116 (2006).
